# The *Salmonella* Effector SteD Mediates MARCH8-Dependent Ubiquitination of MHC II Molecules and Inhibits T Cell Activation

**DOI:** 10.1016/j.chom.2016.10.007

**Published:** 2016-11-09

**Authors:** Ethel Bayer-Santos, Charlotte H. Durkin, Luciano A. Rigano, Andreas Kupz, Eric Alix, Ondrej Cerny, Elliott Jennings, Mei Liu, Aindrias S. Ryan, Nicolas Lapaque, Stefan H.E. Kaufmann, David W. Holden

**Affiliations:** 1 MRC Centre for Molecular Bacteriology and Infection, Imperial College London, Armstrong Road, London SW7 2AZ, UK; 2 Department of Immunology, Max Planck Institute for Infection Biology, 10117 Berlin, Germany; 3 INRA, UMR 1319 Micalis, Domaine de Vilvert, Jouy-en-Josas 78352, France; 4 AgroParisTech, UMR Micalis, Jouy-en-Josas 78350, France; 5 Centre for Biosecurity and Tropical Infectious Diseases, Australian Institute of Tropical Health and Medicine, James Cook University, McGregor Road, Cairns, QLD 4878, Australia

**Keywords:** major histocompatibility complex, dendritic cells, *Salmonella*, effector, ubiquitin, ligase

## Abstract

The SPI-2 type III secretion system (T3SS) of intracellular *Salmonella enterica* translocates effector proteins into mammalian cells. Infection of antigen-presenting cells results in SPI-2 T3SS-dependent ubiquitination and reduction of surface-localized mature MHC class II (mMHCII). We identify the effector SteD as required and sufficient for this process. In Mel Juso cells, SteD localized to the Golgi network and vesicles containing the E3 ubiquitin ligase MARCH8 and mMHCII. SteD caused MARCH8-dependent ubiquitination and depletion of surface mMHCII. One of two transmembrane domains and the C-terminal cytoplasmic region of SteD mediated binding to MARCH8 and mMHCII, respectively. Infection of dendritic cells resulted in SteD-dependent depletion of surface MHCII, the co-stimulatory molecule B7.2, and suppression of T cell activation. SteD also accounted for suppression of T cell activation during *Salmonella* infection of mice. We propose that SteD is an adaptor, forcing inappropriate ubiquitination of mMHCII by MARCH8 and thereby suppressing T cell activation.

## Highlights

• *Salmonella* effector SteD promotes ubiquitination and surface depletion of mature MHCII
• SteD binds both MHCII and the host E3 ubiquitin ligase MARCH8
• SteD uses MARCH8 to ubiquitinate and surface deplete MHCII
• SteD suppresses T cell activation during *Salmonella* infection in vitro and in mice

## Graphical Abstract

**Figure:**
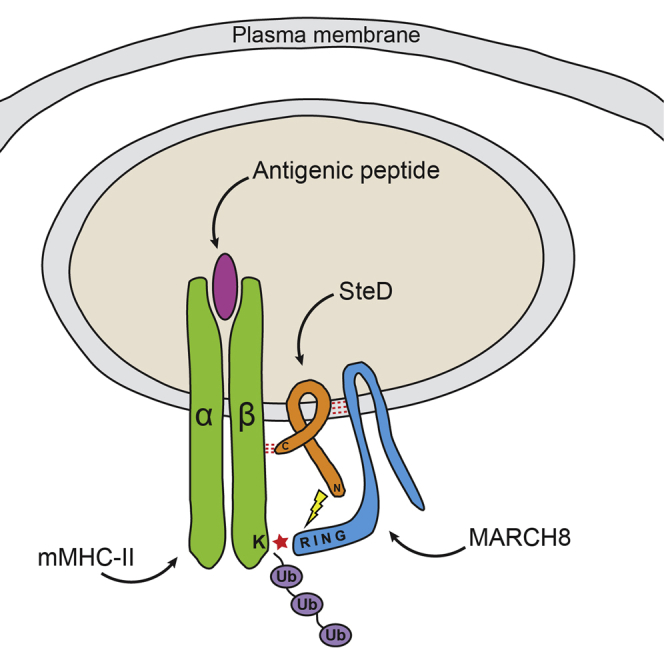

## Teaser

Dendritic cell infection by *Salmonella* depletes mature MHC class II (mMHCII) from the cell surface. Bayer-Santos et al. reveal that the *Salmonella* effector SteD binds the E3 ligase MARCH8 and mMHCII to promote mMHCII ubiquitination and surface depletion. SteD suppressed dendritic cell-mediated T cell activation in vitro and in mice.

## Introduction

Major histocompatibility complex class II (MHCII) molecules have a pivotal function in adaptive immunity by displaying antigenic peptides on the surface of antigen-presenting cells such as dendritic cells (DCs) to CD4-restricted T cells, leading to their activation, proliferation, and differentiation. MHCII complexes consist of heterodimers of α and β chains that assemble in the endoplasmic reticulum (ER) along with the chaperone invariant chain (Ii) to form a larger structure called the immature MHCII complex (Ii-MHCII). This complex migrates through the Golgi network to the plasma membrane and a late endosomal MHCII compartment, where Ii is degraded by lysosomal proteases, leaving the CLIP peptide in the MHCII peptide-binding groove of the α/β heterodimer. Class II related chaperones HLA-DM and HLA-DO exchange CLIP for antigenic peptides to form mature MHCII (mMHCII) complexes. These are transported to the cell surface for antigen presentation (Neefjes et al., 2011). Surface-localized mMHCII can undergo internalization and recycling back to the plasma membrane (Roche and Furuta, 2015). In immature DCs, the E3 ligase MARCH1 ubiquitinates internalized mMHCII, leading to its incorporation into multivesicular bodies and lysosomal degradation (Cho et al., 2015). Activation of DCs is accompanied by reduced expression of MARCH1, enabling internalized mMHCII to recycle back to the plasma membrane (De Gassart et al., 2008).

After oral ingestion, *Salmonella enterica* encounters DCs in Peyer’s patches of the small intestine (Tam et al., 2008). Following uptake by DCs, the majority of bacteria remain within a membrane bound compartment, the *Salmonella*-containing vacuole (SCV), from where they deliver effector proteins to the host cell via the SPI-2 type III secretion system (T3SS) (Jantsch et al., 2003).

It has been known for over a decade that *Salmonella* inhibits the process of antigen presentation by mMHCII molecules in DCs (Cheminay et al., 2005, Halici et al., 2008, Jackson et al., 2013, Lapaque et al., 2009a, Mitchell et al., 2004, Tobar et al., 2004, Tobar et al., 2006). This is dependent on a functional SPI-2 T3SS (Cheminay et al., 2005, Mitchell et al., 2004). Mutant strain analysis showed that several effectors affecting vesicular trafficking disrupt T cell proliferation (Cheminay et al., 2005, Halici et al., 2008). Another study revealed that in *Salmonella-*infected cells, surface levels of mMHCII are reduced through its ubiquitination (Jackson et al., 2013, Lapaque et al., 2009a). However, the effector(s) and mechanisms involved in the regulation of mMHCII ubiquitination are unknown.

Here, we show that SteD is a SPI-2 T3SS effector that is required and sufficient for depletion of mMHCII from the surface of infected cells. Further work showed that SteD is an integral membrane protein that interacts with the E3 ubiquitin ligase MARCH8 and mMHCII. SteD stimulates MARCH8-mediated ubiquitination of the β chain of mMHCII and accounts for the ability of *Salmonella* to inhibit T cell responses.

## Results

### SteD Reduces Surface Levels of mMHCII

**Figure 1:**
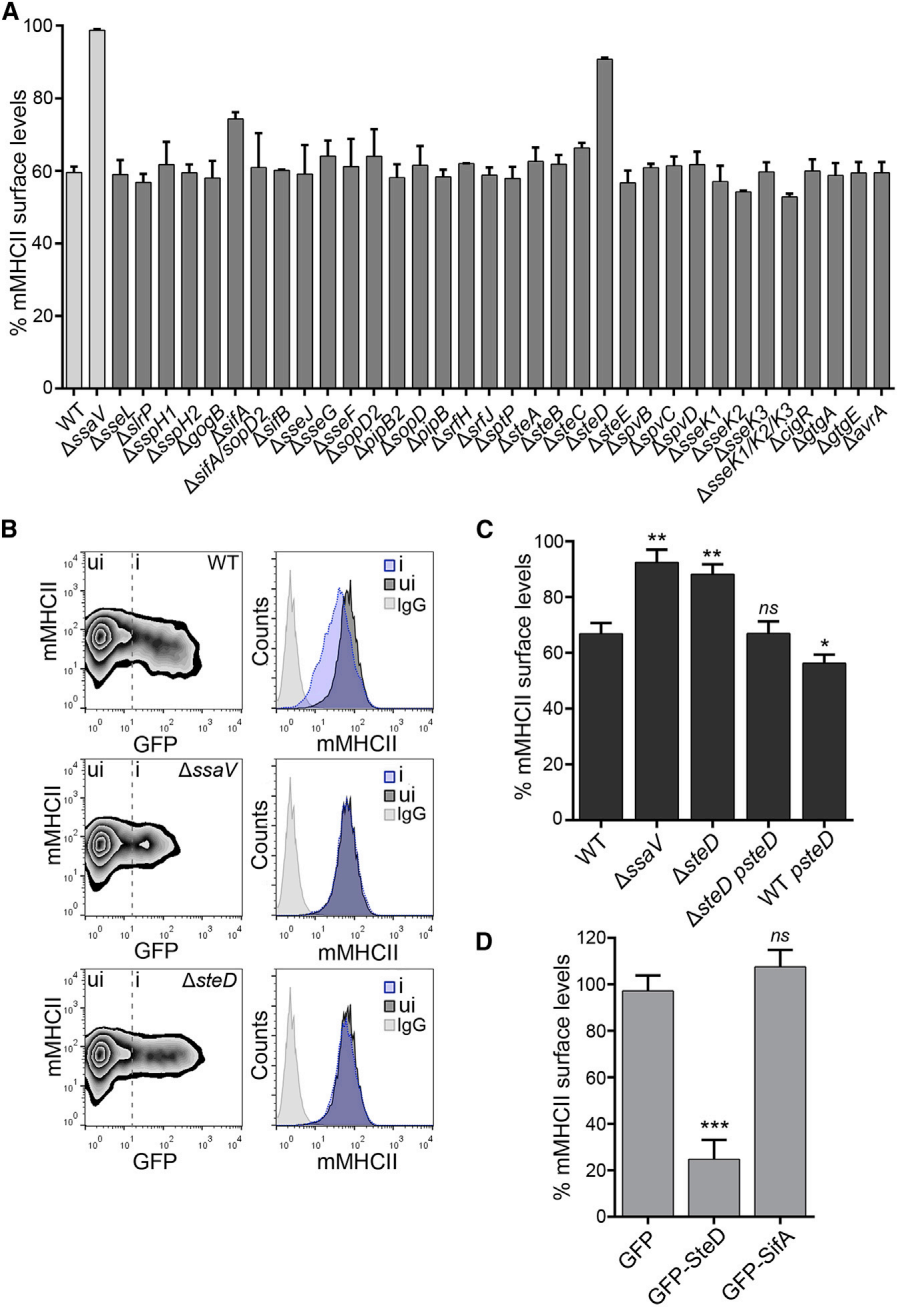
*Salmonella* SPI-2 T3SS Effector SteD Reduces Surface Levels of Mature MHCII Molecules (A) Mel Juso cells were infected with WT or mutant *Salmonella* strains for 16 hr and surface levels of mMHCII were measured by flow cytometry using mAb L243 (that specifically recognizes mature HLA-DR). The error bars represent SD of the geometric mean fluorescence of two independent experiments performed in duplicate. (B) Representative FACS plots showing surface levels of mMHCII in infected cells (i) compared to uninfected cells (ui). The histograms show surface levels of mMHCII in infected (i, blue) and uninfected (ui, dark gray) cells. The cells labeled with isotype control antibody are shown in light gray. (C) Mel Juso cells were infected with Δ*steD* strain carrying pWSK29, expressing SteD-2HA regulated by its endogenous promoter (*psteD*). The infected and uninfected cells were discriminated using anti-*Salmonella* CSA-1 antibody after fixation. The data represent surface levels of mMHCII in infected cells as a percentage of those in uninfected cells from the same sample. (D) Mel Juso cells were transfected with vectors encoding GFP-tagged effectors, and mMHCII was analyzed by flow cytometry. The data represent surface levels of mMHCII in transfected cells as a percentage of those in untransfected cells from the same sample. (C and D) Error bars represent SD of the geometric mean fluorescence of three experiments done in duplicate and were analyzed through comparison with WT *Salmonella* (C) or GFP-only transfected cells (D) by one-way ANOVA followed by Dunnett’s multiple comparison test. ^∗∗∗^p < 0.001, ^∗∗^p < 0.01, ^∗^p < 0.05, and not significant, *ns*.

To identify *Salmonella* SPI-2 T3SS effector(s) involved in the removal of mMHCII molecules from the surface of infected cells, we used a collection of mCherry-expressing mutant strains lacking individual SPI-2 T3SS effectors to infect human Mel Juso cells. This cell line is widely used to study MHC class II trafficking and presentation. Three human MHCII isotypes exist: HLA-DR, HLA-DQ, and HLA-DP. mMHCII surface levels were measured by flow cytometry using mAb L243, which recognizes mature HLA-DR (Bijlmakers et al., 1994). Of the panel of 33 single mutants, a double, and a triple mutant, all strains reduced surface mMHCII to approximately the same degree as the wild-type (WT) strain, with the exception of Δ*ssaV*, Δ*sifA*, and Δ*steD* strains (Figure 1A). SsaV is an essential component of the SPI-2 secretion apparatus, and its absence prevents bacteria from translocating all T3SS effectors. Vacuoles harboring Δ*sifA* bacteria are unstable, whereas the majority of vacuoles containing Δ*sifA/*Δ*sopD2* bacteria remain intact (Schroeder et al., 2010). The surface levels of mMHCII in cells infected with the Δ*sifA/*Δ*sopD2* mutant were similar to those caused by the WT strain, suggesting that the effect of the Δ*sifA* mutant is likely to be indirect, resulting from loss of the vacuolar membrane. We created a second *steD* deletion mutant expressing GFP and tested its effect on surface levels of mMHCII in infected Mel Juso cells. There was a reduction of mMHCII in cells infected with GFP-expressing WT bacteria (Figure 1B, i) compared to uninfected cells (Figure 1B, ui), but no difference was detected in Δ*ssaV* or Δ*steD* infected cells (Figure 1B, i) compared to uninfected cells in the same sample (Figure 1B, ui). To establish if the lack of effect of Δ*steD* on mMHCII was due to the absence of *steD* and not to an adventitious mutation or polar effect, the mutant strain was transformed with a low copy number plasmid (pWSK29) encoding SteD-2HA under the control of its endogenous promoter. This strain (Δ*steD psteD*) removed surface mMHCII to the same extent as the WT strain (Figures 1C and S5A). The WT strain carrying *psteD-2HA* further reduced mMHCII surface levels (Figure 1C). The similar phenotypes of the Δ*steD* and Δ*ssaV* mutants suggest that SteD accounts for all of the SPI-2 T3SS-mediated effect. Furthermore, ectopic expression of GFP-tagged SteD or SifA in Mel Juso cells showed that SteD specifically reduced mMHCII from the cell surface in the absence of other SPI-2 effectors (Figures 1D and S5B). From these experiments, we conclude that SteD is required and sufficient for the reduction of surface levels of mMHCII in Mel Juso cells.

### Localization and Topology of SteD

SPI-2 T3SS-dependent translocation of SteD was shown using CyaA reporter fusions (Niemann et al., 2011). *steD* is present in the genome of several serovars of *S. enterica*, including *S.* Typhimurium, *S.* Enteritidis, *S.* Gallinarum, *S.* Paratyphi, and *S.* Typhi (Figure S1A). No similarity of predicted amino acid sequences to proteins of any other bacterial genera was found by BLAST analysis.

To determine the subcellular localization of SteD after its translocation or ectopic expression, we infected Mel Juso cells with *Salmonella* Δ*steD psteD-2HA* or transfected cells with a plasmid encoding GFP-SteD. Immunofluorescence microscopy revealed that SteD accumulated in the region of the Golgi network, as shown by co-localization with the *trans*-Golgi network (TGN) protein TGN46 (Figure S1B). Translocation of SteD-2HA from bacteria depended on a functional SPI-2 T3SS (Figure S1C). To test if SteD localizes to Golgi membranes, Mel Juso cells were exposed to Brefeldin A (BFA) to disassemble the Golgi network, and the localization of SteD was examined by immunofluorescence microscopy together with *cis*- and TGN proteins GM130 and TGN46, respectively (Figure S1D). Along with TGN46 and GM130, SteD dispersed after exposure of cells to BFA. Furthermore, SteD relocalized to a reformed Golgi network 90 min after BFA washout (Figure S1D). There was significantly greater co-localization between SteD and TGN46 than with GM130 (Figure S1B, right), indicating that it accumulates predominantly in the TGN. Live cell imaging analysis of Mel Juso cells expressing GFP-SteD revealed that the majority of effector remained clustered and relatively immobile (consistent with TGN localization). However, a second pool of SteD was also observed in tubular/vesicular structures that were highly dynamic, moving extensively throughout cells in anterograde and retrograde directions (Movie S1).

**Figure 2:**
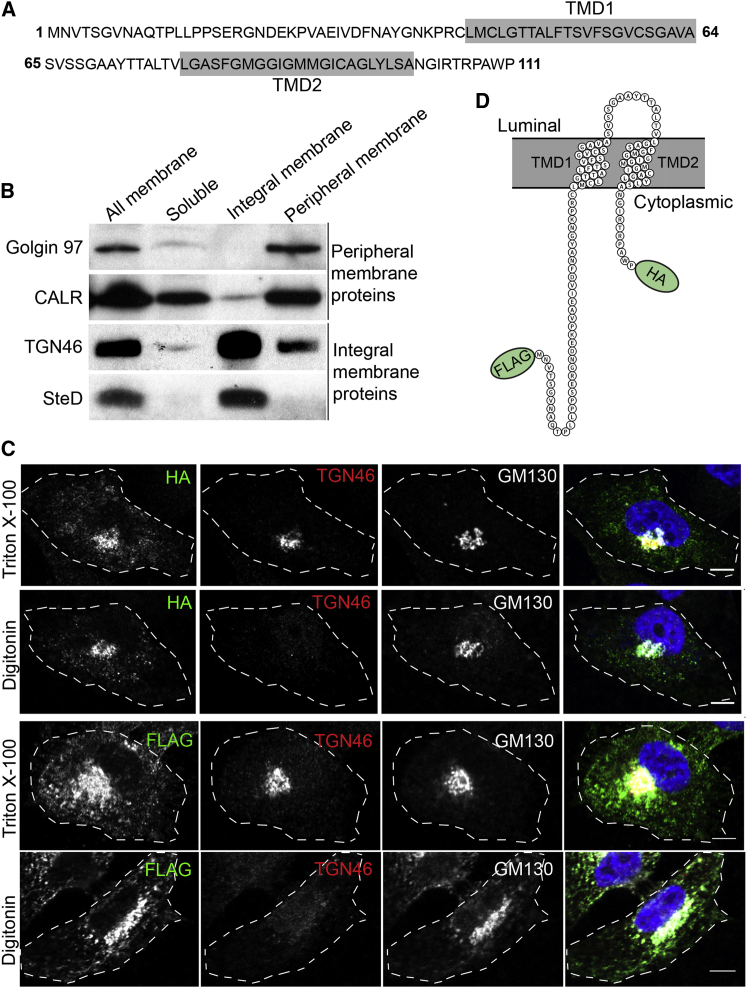
SteD Is an Integral Membrane Protein and Both the N- and C-termini Are Exposed to the Host Cell Cytosol (A) Amino acid sequence of SteD showing transmembrane domains (TMD) predicted by TMHMM 2.0 software shaded in gray. (B) Membrane fractionation of Mel Juso cells infected for 20 hr with Δ*steD psteD*-*2HA*. The soluble proteins were separated from total membrane proteins, which were later treated with 2.5 M urea to discriminate between the integral membrane and membrane-associated proteins by ultracentrifugation. Calreticulin (CALR) and Golgin 97 are membrane-associated proteins and TGN46 is an integral Golgi membrane protein. (C) Mel Juso cells were infected with Δ*steD psteD*-*2*HA (top two panels) or transfected with vector encoding SteD fused at its N terminus to FLAG epitope (FLAG-SteD) (bottom two panels). The cells were semi- or completely permeabilized with digitonin or Triton X-100 to discriminate between cytoplasmic and Golgi luminal antigens, respectively. The antibodies recognizing the luminal portion of TGN46 or cytoplasmic GM130 were used as controls. The scale bar represents 5 μm. (D) Schematic representation of SteD topology in the membrane (Protter 1.0 software; Omasits et al., 2014).

SteD is a small protein comprising 111 amino acids (∼12 kDa) and is predicted by TMHMM v2.0 software (Krogh et al., 2001) to be an integral membrane protein (Figure 2A). To test this experimentally, Mel Juso cells were infected with Δ*steD psteD-2HA Salmonella* and subjected to membrane fractionation. Using calreticulin (CALR) and Golgin 97 as controls for peripheral membrane proteins and TGN46 as an integral membrane protein, western blot analysis revealed that SteD was highly enriched in the fraction comprising transmembrane proteins (Figure 2B). To establish the topology of SteD, we used C-terminal HA-tagged effector translocated by the bacteria (Δ*steD psteD-2HA*) and N-terminal FLAG-tagged effector (FLAG-SteD) ectopically expressed in Mel Juso cells in membrane permeabilization experiments. Both N- and C-terminal epitope tags were detected after selective permeabilization of the plasma membrane with digitonin (Figure 2C), showing that SteD has two transmembrane regions and that both the N- and C-termini are exposed to the host cell cytosol (Figure 2D).

### SteD Reduces Surface Levels of mMHCII by Increasing Ubiquitination of Residue K225 on the Cytoplasmic Tail of the β Chain

**Figure 3:**
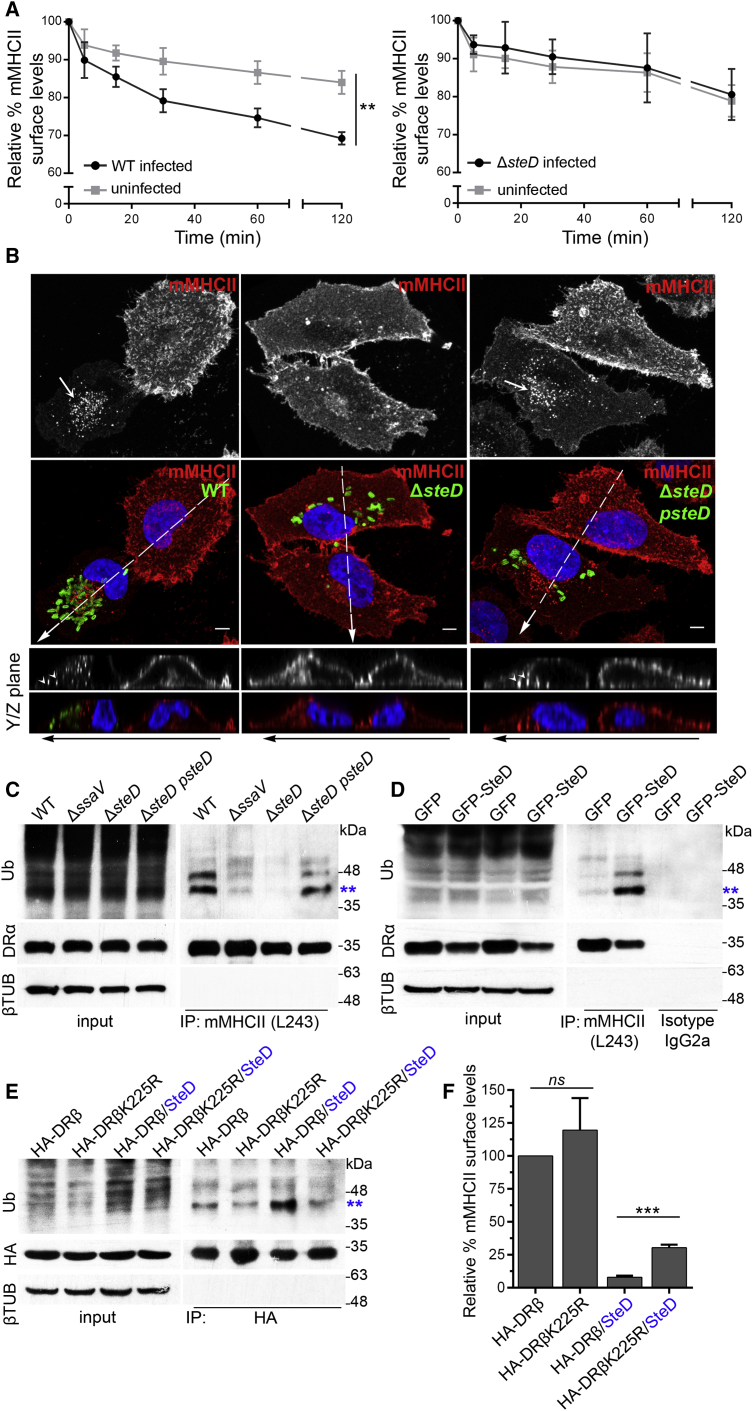
SteD Depletes Surface Levels of mMHCII and Increases Its Ubiquitination (A) Flow cytometry analysis showing surface mMHCII in Mel Juso cells infected with WT-GFP or Δ*steD-*GFP strains compared to uninfected cells. The cells were infected for 16 hr and then labeled with mAb L243 on ice for 30 min to block internalization. The cells were exposed to 37°C to enable resumption of internalization. The surface levels of mMHCII at various time points were normalized to those at the time of transfer to 37°C (0 min), which represents 100%. (B) Cells at 16 hr post invasion were labeled with mAb L243 on ice and incubated for another 4 hr in complete medium at 37°C. The cells were fixed and processed for immunofluorescence microscopy with DAPI nuclear stain (blue), anti-*Salmonella* CSA-1 (green), and anti-mouse secondary antibody against L243 (red). The images are maximum intensity Z projections showing the difference in mMHCII surface levels between cells infected with WT or Δ*steD psteD* and its intracellular accumulation (arrows). The scale bar represents 5 μm. The regions indicated by white dashed lines of Z projections are shown below in the YZ plane. The internalized mMHCII in cells infected with WT or Δ*steD psteD* strains are indicated by arrowheads. (C) Mel Juso cells were infected at an MOI of 300:1 and at 16 hr post invasion lysed for immunoprecipitation with mAb L243 followed by western blot analysis with anti-ubiquitin (P4D1-HRP), anti-DRα, and anti-β tubulin antibodies. (D) Stable Mel Juso cell lines expressing GFP or GFP-SteD were lysed and mAb L243 or IgG2a isotype control was used for immunoprecipitation. The immunoprecipitates were analyzed by western blot with the same antibodies as in (C). (E) HA-tagged DRβ constructs (either wild-type [HA-DRβ] or with an arginine substitution of lysine 225 on DRβ cytoplasmic tail [HA-DRβ K225R]) were used to transduce Mel Juso cells or stable cells expressing GFP-SteD. The cells were lysed and immunoprecipitated with anti-HA antibody coupled to agarose beads followed by western blot analysis with anti-HA, anti-ubiquitin (P4D1-HRP), and anti-β tubulin antibodies. The blot shown is representative of three separate experiments in which band intensities, normalized to corresponding HA bands, are 1.00 ± 0.02 (DRβ), 0.59 ± 0.06 (DRβK225), 1.42 ± 0.44 (DRβ/SteD), and 0.50 ± 0.13 (DRβK225/SteD) (Student’s t test p < 0.03, comparison between DRβ/SteD and DRβK225/SteD). Double asterisks in (C)–(E) denote di-ubiquitinated DRβ. (F) Surface levels of mMHCII on stable cells described in (E), measured by mAb L243 labeling and flow cytometry. The data shown are surface levels of mMHCII relative to that in cells expressing HA-DRβ. ^∗∗∗^p < 0.001, ^∗∗^p < 0.01, and not significant, *ns* (Student’s t test). The data shown are surface levels of mMHCII relative to that in cells expressing HA-DRβ.

To determine if SteD affects the internalization and recycling process of surface-localized mMHCII, Mel Juso cells were infected with WT or Δ*steD* mutant bacteria and then incubated with unconjugated mAb L243 on ice for 30 min to label surface mMHCII. Subsequently, cells were washed and transferred to 37°C to reinitiate internalization/recycling and the amount of mMHCII remaining on the surface at different time points was analyzed by incubating cells with an Alexa Fluor-conjugated secondary antibody to mAb L243 and flow cytometry. In agreement with previous results (Lapaque et al., 2009a), WT *Salmonella*-infected cells underwent a greater rate of loss of surface mMHCII compared to uninfected cells; however, this difference was not observed when cells were infected with the Δ*steD* strain (Figure 3A). Confocal immunofluorescence microscopy of cells at the 4 hr time point revealed intracellular mMHCII in cells infected by WT *Salmonella* and Δ*steD psteD* strains, but not in cells infected by the Δ*steD* mutant (Figure 3B, Y-Z planes). These results show that SteD affects cell surface internalization and/or recycling of mMHCII.

*Salmonella* induces ubiquitination of a lysine residue (K225) in the cytosolic tail of the DRβ chain, and K225 is required for reduction of surface levels of mMHCII by *Salmonella* (Lapaque et al., 2009a). To investigate the involvement of SteD in this process, ubiquitination of mMHCII in infected cells and in cells stably expressing GFP or GFP-SteD was examined by immunoprecipitation of mMHCII and immunoblotting. Immunoprecipitates from cells infected by WT or Δ*steD psteD* strains contained ubiquitinated protein(s) whose levels were reduced or absent in cells infected by Δ*ssaV* and Δ*steD* strains (Figure 3C). Stable expression of GFP-SteD in Mel Juso cells also led to ubiquitination of immunoprecipitated protein(s) (Figure 3D). An anti-HA antibody was then employed to immunoprecipitate HA-tagged DRβ (either wild-type [HA-DRβ] or a mutant in which K225 was substituted with arginine [HA-DRβ K225R]) from Mel Juso cells stably expressing these constructs. There was an increase in ubiquitination of immunoprecipitated protein(s) in cells expressing GFP-SteD and HA-DRβ (DRβ/SteD) compared to cells lacking SteD (DRβ) (Figure 3E). The size of the most prominent band corresponds to di-ubiquitinated DRβ. No such SteD-dependent increase in ubiquitinated proteins was detected in immunoprecipitates from cells expressing HA-DRβ K225R, indicating that K225 is the target of SteD-induced ubiquitination (Figure 3E). The total surface content of mMHCII in these cells could comprise both endogenous and HA-tagged proteins. There was no detectable difference in surface mMHCII (using mAb L243) between cells expressing endogenous proteins together with HA-DRβ or HA-DRβ K225R in the absence of SteD (Figure 3F), presumably reflecting a low turnover in these cells. SteD reduced surface mMHCII levels by approximately 90% in cells expressing HA-DRβ, but only by approximately 70% in cells expressing HA-DRβ K225R (Figures 3F and S5C). This shows that HA-DRβ K225R is at least partially resistant to SteD-dependent modification and suggests that the reduction of surface mMHCII in HA-DRβ K225R-expressing cells is due to the action of SteD on endogenous mMHCII.

### SteD Interacts with MARCH8 and mMHCII

**Figure 4:**
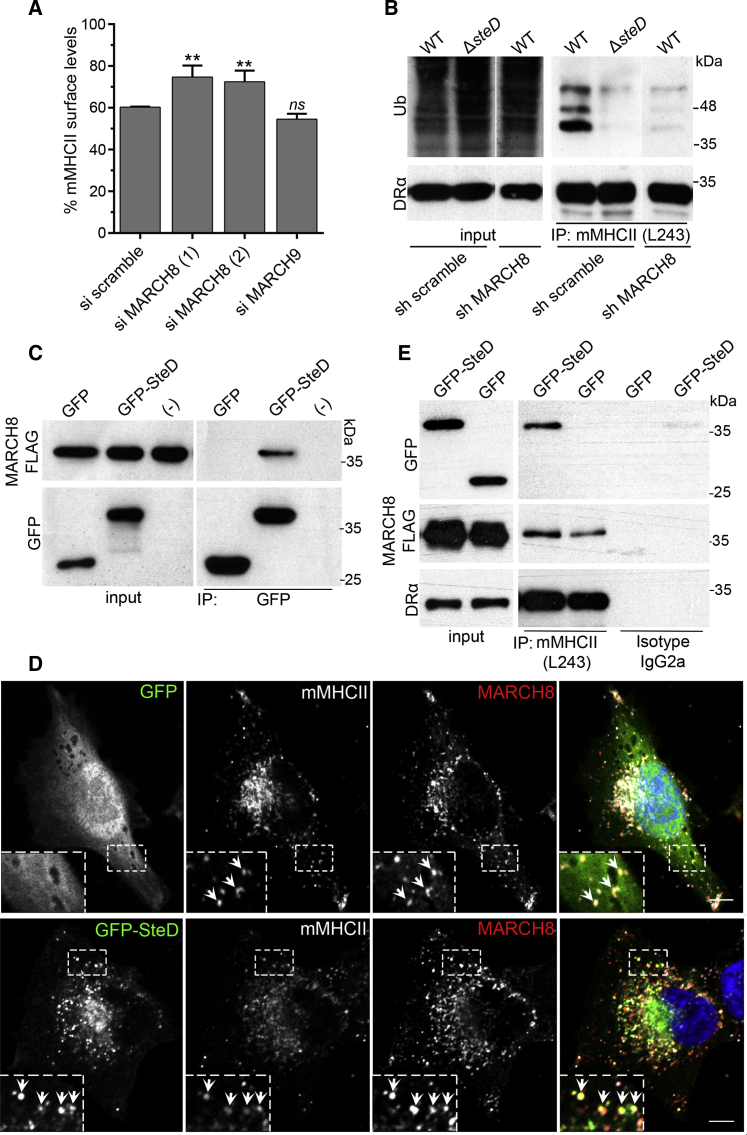
Interactions between SteD, MARCH8, and mMHCII (A) Flow cytometry analysis of Mel Juso cells that were treated with siRNA prior to infection with WT-GFP *Salmonella*. The data represent surface levels of mMHCII in infected cells as a percentage of those in uninfected cells from the same sample and are the means ± SD from three independent experiments. (B) Mel Juso cells transduced with shRNA to knockdown MARCH8 or a scramble control were infected at an MOI of 300:1 and at 16 hr post invasion lysed for immunoprecipitation with mAb L243 followed by western blot analysis with anti-ubiquitin (P4D1-HRP) and anti-DRα. sh MARCH8 and sh scramble images after immunoprecipitation (IP) are from the same blot and exposure. (C) HEK293T cells transfected with vectors expressing MARCH8-FLAG and GFP-SteD or GFP alone were used for immunoprecipitation with GFP-Trap beads. (−) indicates MARCH8-FLAG alone. The immunoprecipitates were analyzed by western blot using anti-GFP and anti-FLAG antibodies. (D) Stable Mel Juso cells expressing GFP-SteD and MARCH8-FLAG were analyzed by immunofluorescence with anti-FLAG (red) and mAb L243 against mMHCII (gray), the arrows indicate vesicles in which the three proteins co-localized. The scale bar represents 5 μm. (E) Stable Mel Juso cell lines expressing both MARCH8-FLAG and GFP-SteD or MARCH8-FLAG and GFP alone were used for immunoprecipitation with mAb L243 (for mMHCII) or isotype control. The immunoprecipitates were analyzed by western blot using anti-GFP, anti-FLAG, and anti-DRα antibodies. The samples in (A) were compared to siRNA scramble by one-way ANOVA followed by Dunnett’s multiple comparison test. ^∗∗^p < 0.01 and not significant, *ns*.

The amino acid sequence of SteD does not suggest an enzymatic activity or other function that would explain the ubiquitination of mMHCII. Therefore, we hypothesized that the enzyme causing SteD-dependent mMHCII ubiquitination was likely to be derived from the host cell. Two highly related members of the MARCH family of E3 integral membrane ubiquitin ligases (MARCH1 and MARCH8, which share 79.4% similarity; Fujita et al., 2013) reduce surface levels of mMHCII when overexpressed in Mel Juso cells (Lapaque et al., 2009b). MARCH1 is expressed in immature DCs and its expression is downregulated upon DC activation, leading to stabilization of mMHCII on the cell surface (De Gassart et al., 2008). In contrast, MARCH8 is expressed in a broad range of cell types, including Mel Juso cells (van de Kooij et al., 2013). However, Mel Juso cells do not express MARCH1 (Lapaque et al., 2009a). Therefore, we used small interfering (si)RNA to deplete MARCH8 and analyzed the ability of *Salmonella* to reduce surface levels of mMHCII in these cells (Figures 4A and S5D). Depletion of MARCH8, but not MARCH9 (Figure S2A), impaired the capacity of WT *Salmonella* to reduce surface levels of mMHCII (Figure 4A). In addition, infection of Mel Juso cells in which MARCH8 was depleted by small hairpin (sh)RNA (Figure S2B) revealed that SteD-dependent mMHCII ubiquitination requires MARCH8 (Figure 4B).

To investigate if SteD interacts with MARCH8, HEK293T cells (which do not express mMHCII) were transfected with plasmids encoding MARCH8-FLAG and GFP-SteD, then subjected to immunoprecipitation with GFP-Trap beads. MARCH8-FLAG co-immunoprecipitated with GFP-SteD, but not with GFP alone (Figure 4C). Immunofluorescence microscopy of transfected Mel Juso cells revealed that MARCH8-FLAG frequently co-localized with mMHCII in distinct vesicles (Figure 4D). These vesicles also contained GFP-SteD, but not the GFP control (Figure 4D). To investigate physical interactions between SteD, MARCH8, and mMHCII in these cells, mMHCII was immunoprecipitated with mAb L243. mMHCII interacted with GFP-SteD, but not with GFP (Figure 4E). It also interacted with MARCH8-FLAG in the presence or absence of SteD (Figure 4E). Live cell imaging analysis of Mel Juso cells expressing mCherry-MARCH8 and GFP-SteD revealed that both proteins were often present in the same vesicles, moving in both anterograde and retrograde directions (Movie S2; Figures S2C and S2D).

**Figure 5:**
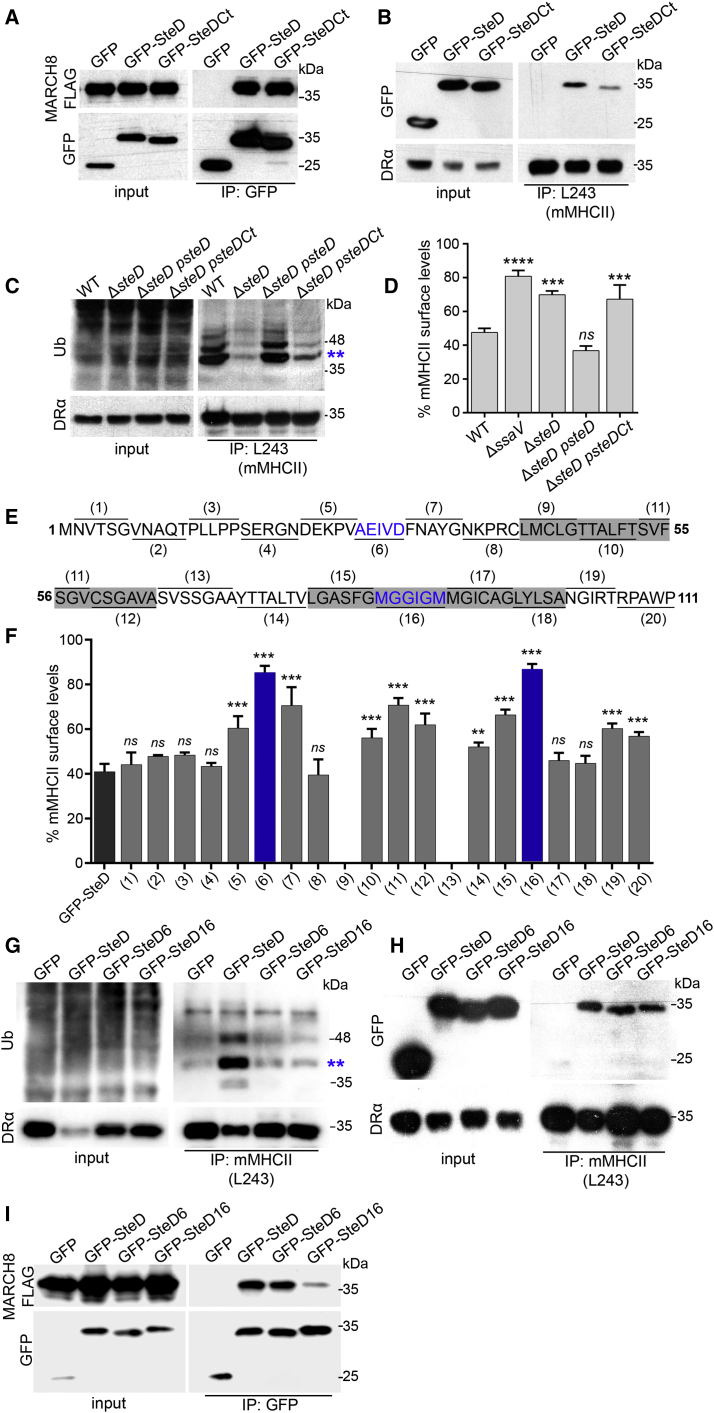
Mutational Analysis of SteD (A) HEK293T cells transfected with vectors expressing MARCH8-FLAG and GFP, GFP-SteD, or GFP-SteD^Ct^ were used for immunoprecipitation with GFP-Trap beads and analyzed by western blot with anti-GFP and anti-FLAG antibodies. (B) Stable Mel Juso cell lines expressing GFP, GFP-SteD, and GFP-SteD^Ct^ were used for immunoprecipitation with mAb L243 (for mMHCII). The immunoprecipitates were analyzed by western blot with anti-GFP and anti-DRα antibodies. The blot shown is representative of three separate experiments in which the mean intensity of GFP-SteD^Ct^ compared to GFP-SteD after immunoprecipitation was 0.21 ± 0.14 (p < 0.02). (C) Mel Juso cells were infected at an MOI of 300:1 and at 16 hr post invasion lysed for immunoprecipitation with mAb L243 followed by western blot analysis with anti-ubiquitin (P4D1-HRP) and anti-DRα antibodies. (D) Mel Juso cells were transfected with vectors encoding GFP, GFP-SteD, and GFP-SteD^Ct^, and mMHCII surface levels were analyzed by flow cytometry. The data represent surface levels of mMHCII in transfected cells as a percentage of those in untransfected cells from the same sample and are the means ± SD from three independent experiments. (E) Schematic representation of SteD amino acids substituted with alanine. The transmembrane regions are shaded in gray. (F) Mel Juso cells were transfected with vectors encoding mutated versions of SteD fused to GFP, and mMHCII surface levels were analyzed by flow cytometry. The data were analyzed as for (D) above. (G) Stable Mel Juso cell lines expressing GFP, GFP-SteD, GFP-SteD^6^, or GFP-SteD^16^ were lysed and mAb L243 was used for immunoprecipitation and analysis as in (C). (H) The stable cells used in (G) were used for immunoprecipitation with mAb L243 (for mMHCII) and analyzed as in (B). (I) HEK293T cells transfected with vectors expressing MARCH8-FLAG and GFP, GFP-SteD, GFP-SteD^6^, or GFP-SteD^16^ were used for immunoprecipitation with GFP-Trap beads and analyzed as in (A). The blot shown is representative of three separate experiments in which the mean intensity of GFP-SteD^16^ compared to GFP-SteD after immunoprecipitation was 0.12 ± 0.07 (p < 0.02). The data in (D) and (F) were compared to GFP-SteD by one-way ANOVA followed by Dunnett’s multiple comparison test. ^∗∗∗∗^p < 0.0001, ^∗∗∗^p < 0.001, ^∗∗^p < 0.01, and not significant, *ns*.

To identify regions of SteD that are important for interacting with mMHCII and MARCH8, we first constructed truncated versions lacking either the N-terminal or C-terminal cytoplasmic domains. The amount of the N-terminal SteD mutant fused to GFP was much lower than that of the wild-type fusion protein, preventing analysis of its properties. However, a version lacking the C-terminal 11 amino acids (GFP-SteD^Ct^) was expressed normally. GFP-SteD^Ct^ also integrated into host cell membranes (Figure S3A), interacted with MARCH8-FLAG (Figure 5A), but had reduced binding to mMHCII (Figure 5B). In infected cells, SteD^Ct^ induced weak ubiquitination of mMHCII (Figure 5C), and after transfection it caused significantly less reduction of mMHCII surface levels (Figure 5D). A more detailed mutational analysis was carried out by making a series of 20 small alanine substitutions (SteD^1^–SteD^20^) in blocks of five or six amino acids, to cover the entire sequence of SteD (Figure 5E). These variants were tagged with GFP and analyzed after transfection to circumvent any defects in their translocation from bacteria. There were two mutants (SteD^9^ and SteD^13^) that were unstable and did not localize at the Golgi network (Figures S4A and S4B). Expression and localization of the remaining 18 GFP-SteD mutants were similar to wild-type SteD (Figures S4A and S4B). Measurement of mMHCII surface levels in cells producing these mutants revealed several regions that are important for SteD function (Figure 5F). The two mutants having the weakest effect on mMHCII surface levels were SteD^6^ and SteD^16^; in both cases this was accompanied by reduced ubiquitination of mMHCII (Figure 5G). SteD^6^ and SteD^16^ integrated into host cell membranes (Figures S3B and S3C) and were not impaired in their ability to interact with mMHCII (Figure 5H). By contrast, in HEK293T cells, SteD^16^, but not SteD^6^, failed to interact efficiently with MARCH8-FLAG (Figure 5I). Together, the mutational analysis of SteD shows that the C-terminal 11 amino acids and second transmembrane domain are important for interactions with mMHCII and MARCH8, respectively. Furthermore, although SteD^6^ interacted with both mMHCII and MARCH8, it failed to induce ubiquitination and removal of surface mMHCII, which suggests that SteD has additional function(s) in regulating MARCH8-dependent ubiquitination of mMHCII.

### SteD-Mediated Reduction of Surface mMHCII Affects T Cell Proliferation

**Figure 6:**
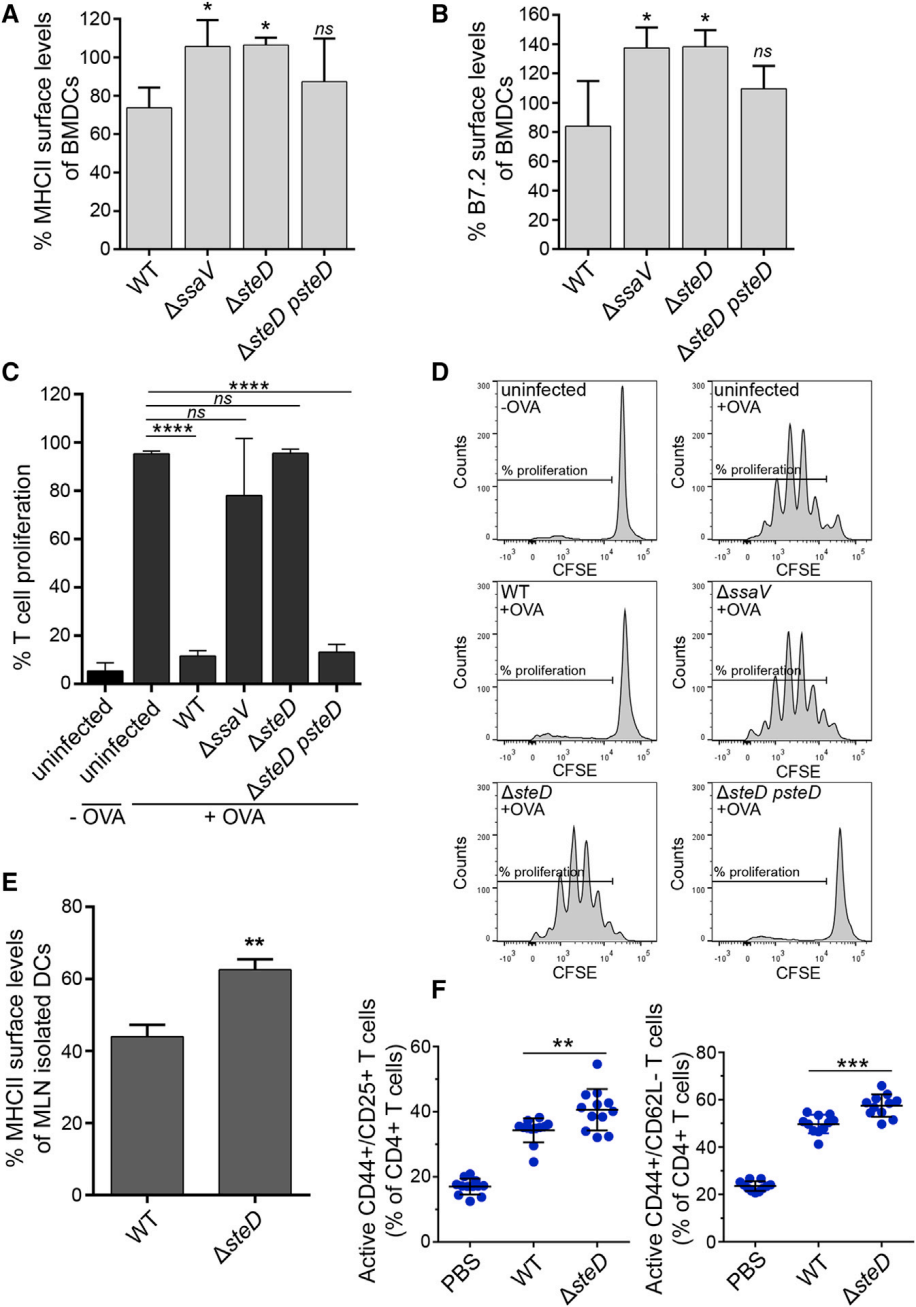
SteD Suppresses T Cell Proliferation (A) Mouse BMDCs were infected with the indicated bacterial strains and total MHCII surface levels (I-A/I-E haplotypes) were quantified by flow cytometry at 20 hr post uptake. (B) BMDCs were infected as in (A), and B7.2 surface levels were quantified by flow cytometry at 20 hr post uptake. (A and B) Surface levels of proteins in infected cells are represented as a percentage of those in uninfected cells from the same sample and are the means ± SD from four independent experiments. (C) Infected BMDCs were incubated with OVA peptide and co-cultured with T cells labeled with CFSE for 3 days. T cell proliferation was analyzed by flow cytometry after labeling cells with anti-CD3, anti-V alpha2, and anti-CD4 antibodies. The uninfected BMDCs incubated or not with OVA-peptide were used as controls. The results shown represent the % of T cells that proliferated and are the means ± SD from quadruplicate samples in three independent experiments. (D) Representative FACS histograms showing T cell proliferation measured by CFSE levels in different conditions. (E) Dendritic cells from mesenteric lymph nodes of mice infected with WT-GFP or Δ*steD*-GFP *Salmonella* by oral gavage were isolated at 48 hr post inoculation and total MHCII surface levels were measured by flow cytometry. The data represent surface levels of MHCII in infected BMDCs as a percentage of those in uninfected cells from the same sample and are the means ± SD from three independent experiments. (F) Activated T cells (CD25+CD44+ and CD62L−CD44+) as a percentage of total CD4^+^ T cells isolated from the spleens of mice infected by *Salmonella* WT or Δ*steD* strains at day 17 post inoculation. The data in (A) and (B) were analyzed by comparison with WT and in (C) by comparison with uninfected +OVA by one-way ANOVA followed by Dunnett’s multiple comparison test. The data in (E) were analyzed by Student’s t test. The data in (F) were analyzed by two-tailed pairwise t test using pairs shown in Table S2. ^∗∗∗∗^p < 0.0001, ^∗∗^p < 0.01, ^∗^p < 0.02, and not significant, *ns*.

Efficient MHCII-dependent T cell activation requires recognition of both peptide-loaded mMHCII and the co-stimulatory molecule CD86/B7.2, whose surface level is also regulated by MARCH8/1 ubiquitination (Bartee et al., 2004, Goto et al., 2003). We confirmed that WT *Salmonella* reduced overall mouse MHCII (I-A/I-E haplotypes) surface levels in bone marrow-derived DCs (BMDCs) and showed that the reduction did not occur in BMDCs infected with Δ*ssaV* or Δ*steD* mutants (Figure 6A). BMDCs infected with WT *Salmonella* also displayed reduced surface levels of B7.2, and this effect was lost following infection with the Δ*ssaV* or Δ*steD* mutants (Figure 6B). To assess the effect of SteD on T cell proliferation, infected BMDCs were incubated with Ovalbumin (OVA)-peptide at 16 hr post uptake, co-cultured for 3 days with T cells expressing a T cell receptor specific for OVA, and labeled with carboxyfluorescein diacetate succinimidyl ester (CFSE) to determine T cell proliferation by flow cytometry. WT *Salmonella* induced a strong SteD-dependent inhibition of T cell proliferation (Figures 6C and 6D), consistent with its effect on both mMHCII and CD86/B7.2. To determine if SteD influences MHCII surface levels during infection of mice, DCs were isolated at 48 hr post oral inoculation from mesenteric lymph nodes. Quantification of total surface MHCII (I-A/I-E) by flow cytometry revealed that DCs infected by the WT-GFP strain had significantly reduced levels of MHCII compared to DCs infected with the Δ*steD*-GFP strain (Figures 6E and S5E). Finally, to assess the effect of SteD on T cell activation in vivo, mice were infected for 17 days with WT or *steD* mutant *Salmonella*, then spleen cells were recovered and T cells analyzed for activation markers by flow cytometry. Analysis of CD4^+^ T cells from mice with similar bacterial loads revealed that there were significantly more activated T cells in spleens carrying the *steD* mutant compared to spleens from mice infected with WT bacteria (Figures 6F and S5F).

## Discussion

Numerous bacterial effectors have been characterized that interfere with distinct aspects of the mammalian innate immune system (McGuire and Arthur, 2015), but much less is known about effectors that inhibit the development of adaptive immunity. Several groups have shown that *Salmonella* inhibits surface presentation of MHCII (Cheminay et al., 2005, Halici et al., 2008, Jackson et al., 2013, Lapaque et al., 2009a, Mitchell et al., 2004). This was attributed to the SPI-2 T3SS (Cheminay et al., 2005, Mitchell et al., 2004) and the actions of several effectors that interfere with vesicular trafficking (Cheminay et al., 2005, Halici et al., 2008). Lapaque et al. (2009a) showed that *Salmonella* specifically reduces the surface levels of mMHCII through its ubiquitination. Several possible mechanisms were proposed to account for this, including delivery of a *Salmonella* ubiquitinating enzyme and the relocalization of mMHCII or host ubiquitin enzymes to facilitate their interactions (Lapaque et al., 2009a). The results presented here, showing that SteD interacts directly or indirectly with both MARCH8 and mMHCII, are consistent with the latter proposal. These interactions might occur in a binary manner, with some SteD molecules interacting with mMHCII and others with MARCH8. However, mutational analysis revealed two distinct regions of SteD that are important for suppressing surface levels of mMHCII: the cytoplasmic C-terminal 11 amino acids are involved in binding mMHCII and part of the second transmembrane domain is important for binding to MARCH8. Both regions are also required for ubiquitination and reduced surface load of mMHCII. This suggests that SteD functions as an adaptor, facilitating interactions between enzyme and substrate. The finding that a transmembrane region of SteD is required for efficient binding to MARCH8 is consistent with other work showing transmembrane domain-dependent recognition of proteins by MARCH1 and MARCH8 (Fujita et al., 2013, Goto et al., 2003) and, more generally, the existence of several integral membrane ubiquitin ligase adaptors that regulate E3 ligase/substrate interactions (Léon and Haguenauer-Tsapis, 2009).

Two results indicate that SteD has additional function(s). First, in Mel Juso cells, similar amounts of MARCH8 interacted with mMHCII in the presence or absence of SteD. Second, the SteD^6^ mutant was defective in ubiquitinating and reducing surface levels of mMHCII, yet retained binding to MARCH8 and mMHCII. This indicates that SteD might also activate MARCH8, which is interesting in light of other evidence suggesting the existence of an endogenous cofactor for MARCH8 (Fujita et al., 2013). Thus, SteD might mimic the activity of an as yet unidentified mammalian membrane adaptor/cofactor that functions with MARCH1/8 to provide substrate specificity and regulate E3 ligase activity.

The finding that the majority of SteD locates to the TGN raises interesting questions regarding its transport from the SCV, Golgi membrane insertion, and the significance of Golgi localization. A substantial amount of MARCH8 co-localized with non-Golgi associated SteD, and vesicles containing both proteins were observed moving throughout the host cell (Figures S2C and S2D). Since mMHCII does not traffic to the Golgi network (Neefjes et al., 2011), the simplest hypothesis is that SteD interacts with MARCH8 in Golgi membranes or Golgi-derived vesicles, and the proteins are then transported to endosomes and possibly to the plasma membrane to interact with mMHCII. Cho et al. (2015) showed that in immature mouse DCs, MARCH1 does not affect the rate of mMHCII endocytosis, but instead prevents its recycling to the plasma membrane from intracellular vesicles. Ubiquitination of mMHCII by MARCH1 in early endosomes promotes its sorting into multivesicular bodies and lysosomal degradation (Cho et al., 2015, Furuta et al., 2013). The presence of SteD and MARCH8 on intracellular vesicles suggests that its effect on mMHCII is to prevent recycling to the plasma membrane, but further work is needed to establish if SteD also acts on the plasma membrane pool of mMHCII.

Whereas the effect of SteD on DC surface MHCII (Figure 6A) appears relatively modest, the inhibition of T cell proliferation was very dramatic (Figure 6C). However, the antibody used to detect surface MHCII on DCs does not discriminate between mMHCII and Ii-MHCII forms and therefore is likely to underestimate the effect of SteD on mMHCII in this assay. Furthermore, we found that SteD also reduces surface levels of the co-stimulatory molecule CD86/B7.2, which is also regulated by MARCH1 (Bartee et al., 2004) and required for full T cell activation. Whether this results from a similar mechanism to that of SteD on mMHCII remains to be determined, but the combined effect of SteD on both mMHCII and CD86/B7.2 provides an explanation for its potent effect on T cell activation (Figure 6C).

MARCH1 expression was reported to be restricted to secondary lymphoid tissues, while MARCH8 is ubiquitously expressed (Bartee et al., 2004). In immature DCs, the expression of MARCH1 is relatively high, enabling it to prevent mMHCII from recycling to the cell surface, but its expression is reduced by over 90% following DC activation (Cho et al., 2015, De Gassart et al., 2008). Conversely, MARCH8 expression remains unchanged during the maturation process (De Gassart et al., 2008) and functions in transferrin receptor recycling (Fujita et al., 2013), but does not seem to contribute to recycling of mMHCII. It is possible that SteD diverts MARCH8 from its normal function or activity to increase ubiquitination of mMHCII in mature DCs after *Salmonella* infection. However, we did not detect a difference in overall surface levels of transferrin receptor in DCs infected with WT or *steD* mutant bacteria (Figure S5G), so the identity of the MARCH ligase targeted by SteD in DCs remains to be established.

SteD accounted for potent inhibition of T cell proliferation by *Salmonella* in vitro. In vivo, DCs from mouse mesenteric lymph nodes infected with the WT bacteria displayed less cell surface MHCII than DCs infected with the *steD* mutant. Furthermore, we detected a significant SteD-dependent suppression of T cell activation in spleens of mice infected with *Salmonella.* Therefore, the in vitro effects of SteD on mMHCII and T cells are likely to be physiologically important. Several bacterial pathogens, including *Salmonella*, encode ubiquitin ligases that target host cell proteins (Ashida et al., 2014). We show here the existence of a distinct mechanism, by which a bacterial effector binds and possibly activates a host E3 ligase to force the inappropriate ubiquitination of a substrate. SteD appears to be the first example of a bacterial protein that targets mMHCII to suppress adaptive immune responses. In view of the critical role of MHCII molecules in the development of adaptive immunity to many bacteria, it is possible that other pathogens might use similar mechanisms to promote their virulence.

## Experimental Procedures

### Bacterial Strains, Plasmids, and Antibodies

All bacterial strains, plasmids, and antibodies used in this study are described in Table S1. Isogenic mutants of *S. enterica* serovar Typhimurium were constructed using one-step chromosomal inactivation system (Datsenko and Wanner, 2000). Wild-type and mutant strains were transformed with plasmids pFPV25.1 and pFCcGi for GFP and mCherry expression, respectively, when required.

### Cell Culture and Infection

Mel Juso and HEK293T cells were maintained in DMEM containing 10% fetal calf serum (FCS) at 37°C in 5% CO_2_. L243-producing hybridoma cells were maintained in DMEM containing 10% FCS and reduced to 5% FCS when cell density was high to harvest supernatant containing antibodies. Primary BMDCs were extracted from C57BL/6 mice (Charles River). Cells recovered from tibias and femurs were grown at 37°C in 5% CO_2_ in RPMI-1640 supplemented with 10% FCS, 2 mM glutamine, 1 mM sodium pyruvate, 10 mM HEPES, 0.05 M β-mercaptoethanol, and 20 ng/mL granulocyte-macrophage colony-stimulating factor (GM-CSF) (PeproTech). After 3 days of culture, fresh complete medium was added to the growing cells. On day 6, medium was replaced by fresh complete medium, and cells were harvested on day 8 and seeded 6 hr prior to infection. Bacteria were grown in Luria Bertani (LB) medium with shaking at 37°C, supplemented with carbenicillin or kanamycin as required (50 μg/mL). Mel Juso cells were infected for 30 min at MOI of 100:1 with late log-phase *Salmonella*. BMDCs were infected for 30 min at MOI 10:1 with stationary-phase *Salmonella* opsonized in 20% mouse serum. Cells were washed with PBS twice and incubated in fresh medium containing gentamicin (100 μg/mL) for 1 hr to kill extracellular bacteria. After 1–2 hr, the antibiotic concentration was reduced to 20 μg/mL, and the cells were processed 16–20 hr post uptake. Approximately 70% of Mel Juso cells and BMDCs became infected using these conditions.

### siRNA and DNA Transfection and Retroviral Transduction

Details of siRNA and shRNA transfection are in the Supplemental Information. Knock down of MARCH8 and MARCH9 mRNA was assessed by qPCR using SYBR GREEN and conditions described previously (Lapaque et al., 2009a). DNA transfection procedures were carried out using Lipofectamine 2000 according to the manufacturer’s protocol (Life Technologies). Samples were prepared for analysis 20 hr after transfection. For retrovirus production, HEK293T cells were transfected with proviral plasmid M5P together with helper plasmids using Lipofectamine 2000 as described previously (Randow and Sale, 2006). Mel Juso cells were transduced with virus in culture supernatant containing 8 μg/mL polybrene (Sigma-Aldrich) by centrifugation at 650 *g* for 1 hr at 37°C. Transduced cells were selected in 2 μg/mL puromycin or GFP-positive cells were sorted by fluorescence-activated cell sorting (FACS) as required.

### Flow Cytometry

To measure surface levels of mMHCII, Mel Juso cells that had been either transfected or infected with *Salmonella* were collected using Cell Dissociation Buffer (Sigma-Aldrich) and incubated first with mAb L243 and then anti-mouse secondary antibody diluted in FACS buffer (5% FCS and 2 mM EDTA in PBS) at 4°C for 30 min. After washings with cold PBS, cells were fixed in 3% paraformaldehyde and analyzed using a FACS Calibur or Fortessa flow cytometer (BD Biosciences) and FlowJo v10 software. mMHCII surface levels were calculated as geometric mean fluorescence of infected cells (GFP-positive)/geometric mean fluorescence of uninfected cells (GFP-negative) × 100. For complementation analysis of bacterial mutants, Mel Juso cells were infected with non-fluorescent bacterial strains and, after labeling of surface mMHCII, cells were fixed and incubated with anti-*Salmonella* CSA-1 antibody diluted in 10% FCS and 0.1% saponin in PBS for 1 hr at room temperature, followed by anti-goat secondary antibody labeling under the same conditions. Surface levels of MHCII or B7.2 in BMDCs were calculated as median fluorescence of infected cells (CSA1-positive)/median fluorescence of uninfected cells (CSA1-negative) × 100. For the internalization assay, Mel Juso cells were infected with WT-GFP or Δ*steD*-GFP *Salmonella* strains and harvested 16 hr post invasion. The surface mMHCII molecules were labeled with mAb L243 for 30 min at 4°C. Cells were washed in cold medium, resuspended, and split into 1.5 mL tubes containing pre-warmed medium in a water bath at 37°C. Aliquots were removed at various time points, diluted in ice-cold FACS buffer, and kept on ice. At the last time point, cells were centrifuged at 4°C and resuspended in FACS buffer containing Alexa 647-conjugated goat anti-mouse antibody. After 30 min at 4°C, cells were washed, fixed in 3% paraformaldehyde, washed, and analyzed by flow cytometry to quantify mMHCII/L243 complexes remaining at the cell surface. GFP-positive cells were considered infected and GFP-negative uninfected. mMHCII surface levels were normalized to those detected at the beginning of the experiment (100%).

### Immunofluorescence Microscopy

Cells were fixed and immunolabeled with antibodies listed in Table S1. Samples were mounted and analyzed using a confocal laser-scanning microscope LSM 710 (Zeiss). Co-localization analysis was done with Zeiss Zen 710 software, with thresholds set using individual controls lacking each analyzed primary antibody. Further details are in Supplemental Information.

### Membrane Fractionation

Approximately 5 × 10^7^ Mel Juso cells were infected as described above. At 20 hr post invasion, cells were collected and lysed by mechanical disruption using a Dounce homogenizer in 600 μL of homogenization buffer containing 250 mM sucrose, 3 mM imidazole (pH 7.4), and 1 mM PMSF. Cell extracts were centrifuged at 1,800 *g* for 15 min and the post nuclear supernatant was centrifuged at 100,000 *g* for 1 hr at 4°C, giving rise to a pellet containing total membrane proteins and supernatant containing soluble proteins. The pellet was resuspended in 600 μL of 2.5 M urea and incubated for 15 min on ice. This suspension was centrifuged again at 100,000 *g* for 1 hr at 4°C, resulting in a second pellet containing integral membrane proteins and a supernatant containing membrane-associated proteins. The volume of all fractions was made to 600 μL with homogenization buffer, and proteins were analyzed by western blot.

### Immunoprecipitation and Western Blot

Analysis of mMHCII ubiquitination was done as described previously with minor modifications (Lapaque et al., 2009a). Further details are in Supplemental Information.

### Live Imaging

For live cell imaging, Mel Juso cells stably expressing GFP-SteD were seeded at a density of approximately 5 × 10^4^ cells per dish onto 35 mm glass-bottom culture dishes (MatTek). Cells were transfected for 20 hr with a vector expressing mCherry-MARCH8 using Lipofectamine 2000 (Life Technologies) following manufacturer’s instructions. Before imaging, cells were washed and incubated in imaging medium (Opti-MEM containing 10% FCS and 25 mM HEPES pH 7.2). Culture dishes were sealed with vaseline and analyzed using a LSM 710 microscope (Zeiss) at 37°C. Samples were imaged with a 63 × oil objective.

### T Cell Proliferation Assay

BMDCs were prepared from C57BL/6 mice as described above. The CD11c-positive cell population was enriched using MAC sorting (Miltenyi Biotec) to a purity of 95%. Cells were infected in 15 mL tubes at an MOI 10:1 for 30 min in RPMI 10% FCS, washed, and treated with gentamicin as described above. After 16 hr of infection, cells were harvested with cold PBS and incubated with OVA peptide (ISQAVHAAHAEINEAGR) at 5 μM in RPMI containing 10% FCS for 1 hr. Cells were washed, counted, and incubated with T cells in 96-well plates. T cells expressing OVA-specific T cell receptor (TCR) were isolated from cell suspensions of spleens and lymph nodes of OT-II mice by magnetic sorting of CD4^+^ cells (Miltenyi Biotec) and labeled with CFSE as described previously (Quah and Parish, 2010). CD4 T cells were incubated with DCs at a ratio of 3:1 in a final volume of 200 μL of medium containing 20 μg/mL gentamicin. At 3 days later, cells were centrifuged and resuspended in 150 μL of FACS buffer containing anti-CD3, anti-V alpha2, and anti-CD4 antibody and incubated for 30 min on ice. Cells were washed and resuspended in 150 μL of FACS buffer containing 6 μm blank calibration particles (BD Biosciences) as an internal control for normalization of T cell numbers.

### Mouse Infection and Isolation of DCs

Female C57BL/6 mice, 6–8 weeks old, were infected by oral gavage with 1 × 10^10^ colony forming units (CFU) of late log-phase GFP-*Salmonella* in 200 μL of PBS containing 3% NaHCO_3_. Mesenteric lymph nodes were isolated 48 hr after inoculation and cells were collected through a 70 μm cell strainer following tissue disruption. DCs were purified from single-cell suspensions using anti-CD11c antibody-coupled magnetic beads (Miltenyi Biotec) according to the manufacturer’s instructions. Purified DCs were labeled on ice with anti-I-A/I-E antibody (recognizing mouse MHC class II molecules), diluted in FACS buffer for 20 min on ice. Purity was assessed by anti-CD11c antibody labeling. Discrimination between infected and uninfected cells was based on GFP fluorescence and MHCII geometric mean fluorescence calculated as described above. For analysis of T cell activation, mice were inoculated intraperitoneally with 5 × 10^5^ CFU of virulence-attenuated *S*. Typhimurium strain SL3261 or SL3261 *ΔsteD* that had been grown in LB broth to late exponential phase. Spleens were harvested 17 days later and homogenized in Hanks balanced salt solution (HBSS) supplemented with 10 mM HEPES and 2% FCS. A portion of spleen homogenate was plated to enumerate bacterial CFU and spleens with similar bacterial loads (Table S2) were analyzed further. Erythrocytes were lysed using ACK buffer (150 mM NH_4_Cl, 10 mM KHCO_3_, and 0.1 mM EDTA). After blocking surface Fc receptors using FcR Blocking Reagent (Miltenyi Biotec), the remaining splenocytes were labeled using anti-CD3ε, anti-CD4, anti-CD25, anti-CD44, and anti-CD62L antibodies. Activation of gated CD4^+^CD3ε^+^ cells was determined on the basis of surface-localized CD25, CD62L, and CD44.

### Ethics Statement

Mice experiments were conducted in accordance to European Directive 2010/63/EU regulations with approval from Imperial College, London Animal Welfare and Ethical Review Body (ICL AWERB) under the Personal Project license of David Holden.

## Author Contributions

E.B.-S., C.H.D., A.K., E.A., O.C., L.A.R., E.J., M.L., and A.S.R. performed research; E.B.-S., C.H.D., A.K., E.A., O.C., and D.W.H. analyzed data; N.L. and S.H.E.K. contributed with discussions; and E.B.-S. and D.W.H. wrote the paper.

## Supplemental Information

Document S1. Supplemental Experimental Procedures, Figures S1–S5, and Tables S1 and S2

Movie S1. Stable Mel Juso Cell Line Expressing GFP-SteD, Related to Figure 2

Movie S2. Stable Mel Juso Cell Line Expressing GFP-SteD Was Transfected with Vector Expressing mCherry-MARCH8 and Movie Was Acquired 20 hr after Transfection, Related to Figure 4

Document S2. Article plus Supplemental Information
