# Supplementary material for: The Salmonella Effector SteD Mediates MARCH8-Dependent Ubiquitination of MHC II Molecules and Inhibits T Cell Activation
Source: Cell Host Microbe. 2016 Nov 9;20(5):584–95. doi: 10.1016/j.chom.2016.10.007 (PMC5104694; doi:10.1016/j.chom.2016.10.007)
Supplement: Document S1. Supplemental Experimental Procedures, Figures S1–S5, and Tables S1 and S2 [file mmc1.pdf]

Cell Host & Microbe, Volume *20*

Supplemental Information

The *Salmonella* Effector SteD Mediates MARCH8-Dependent Ubiquitination of MHC II Molecules and Inhibits T Cell Activation

Ethel Bayer-Santos, Charlotte H. Durkin, Luciano A. Rigano, Andreas Kupz, Eric Alix, Ondrej Cerny, Elliott Jennings, Mei Liu, Aindrias S. Ryan, Nicolas Lapaque, Stefan H.E. Kaufmann, and David W. Holden

Figure S1, related to Figure 2

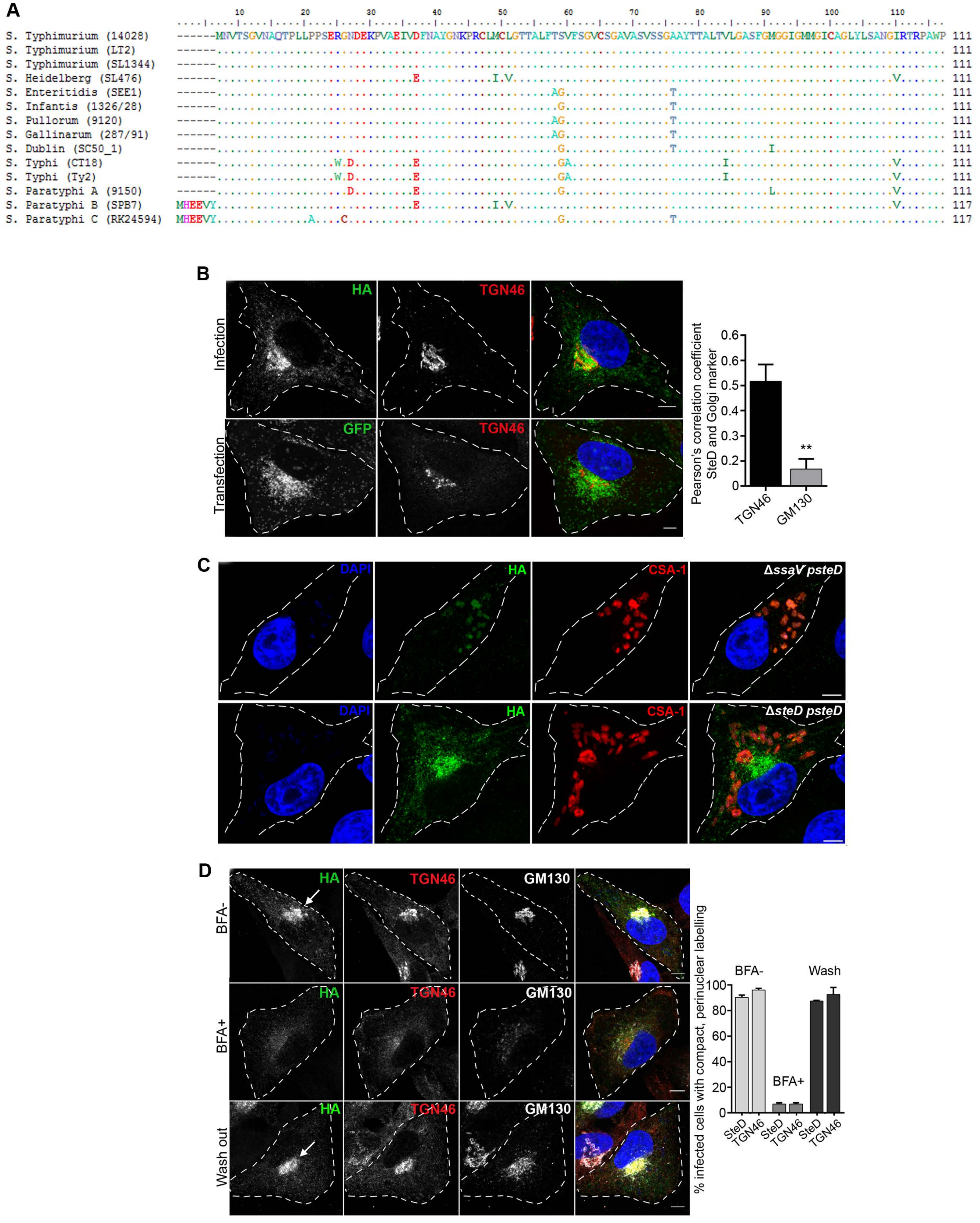

Figure S2, related to Figure 4

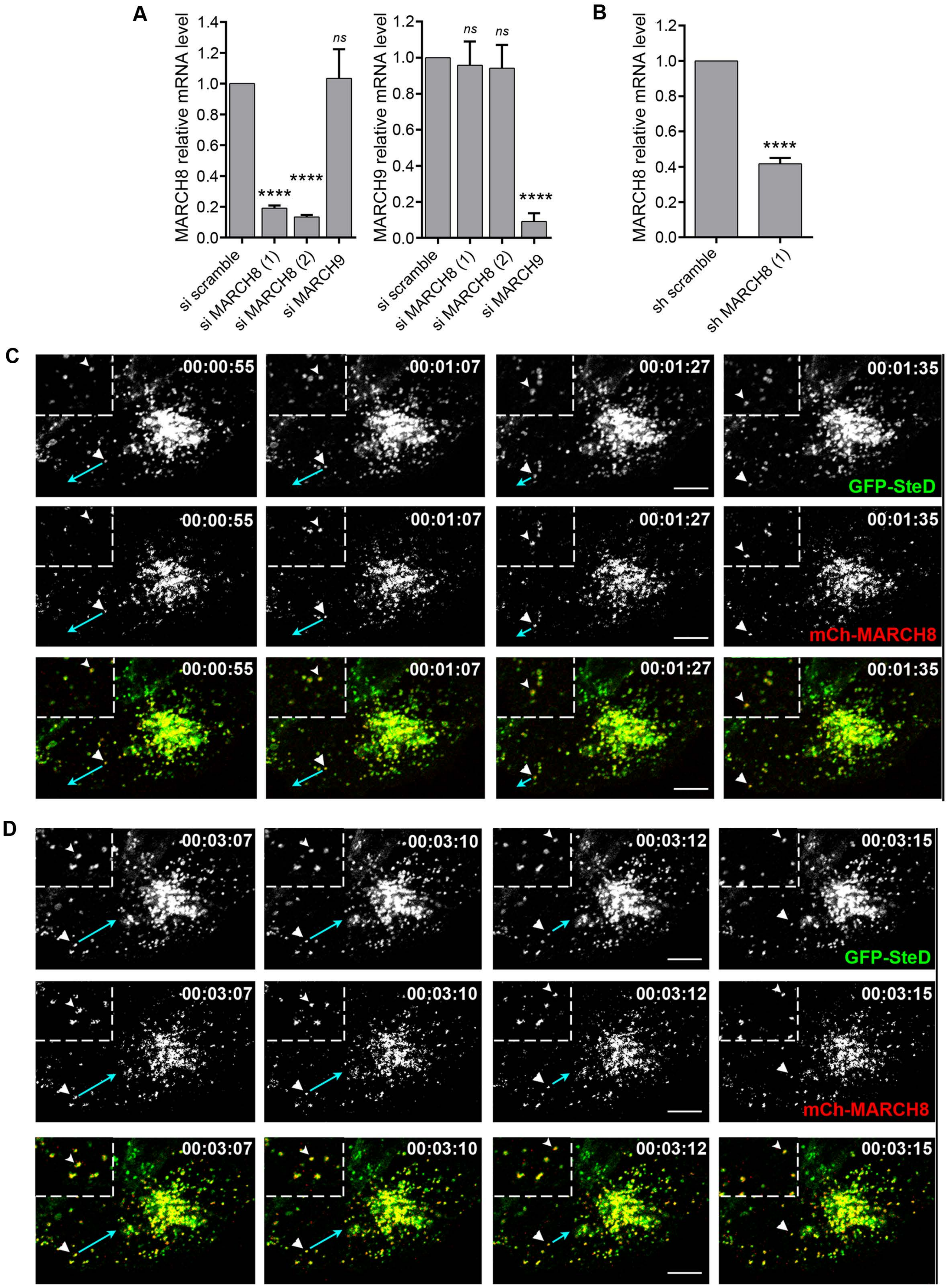

Figure S3, related to Figure 5

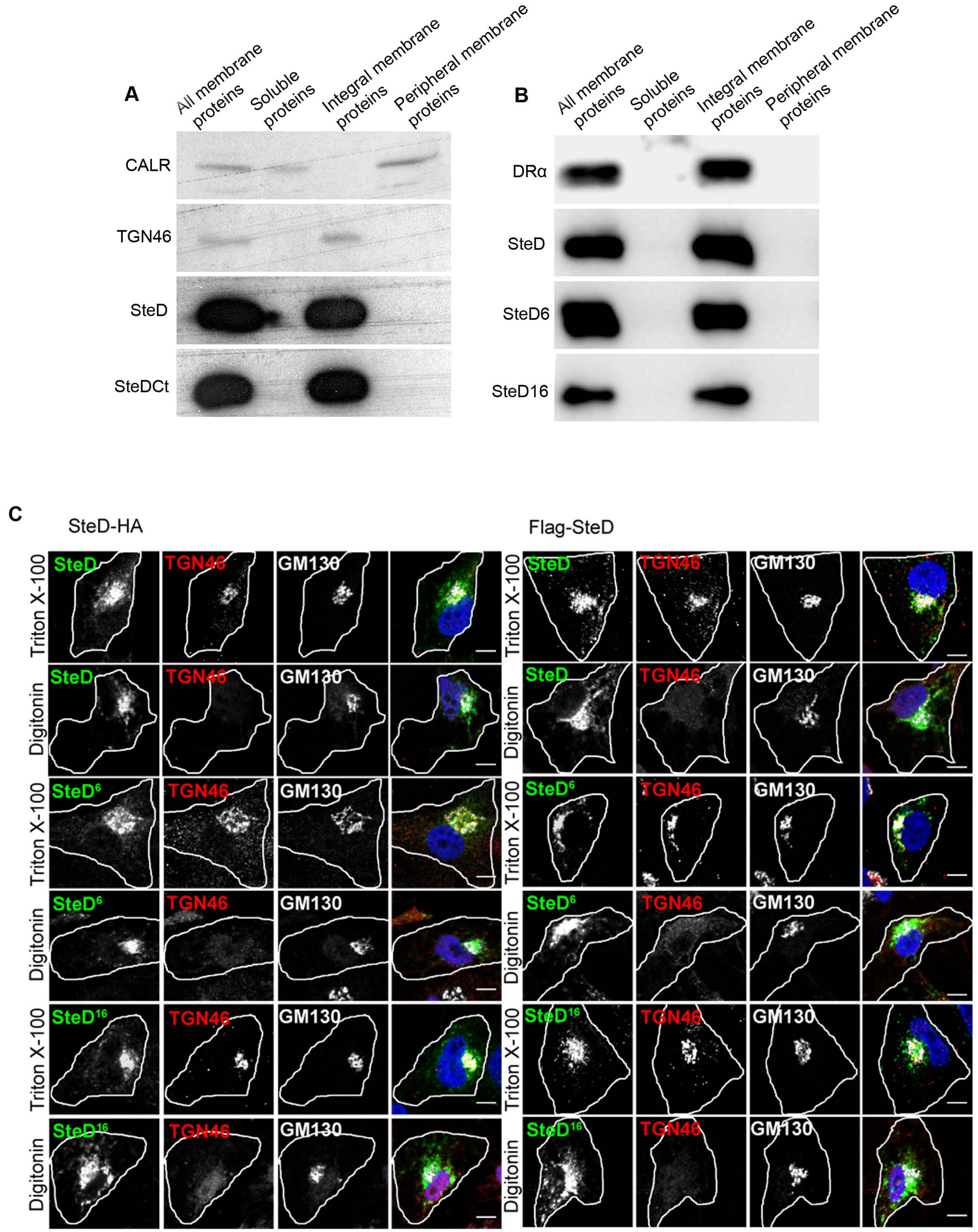

Figure S4, related to Figure 5

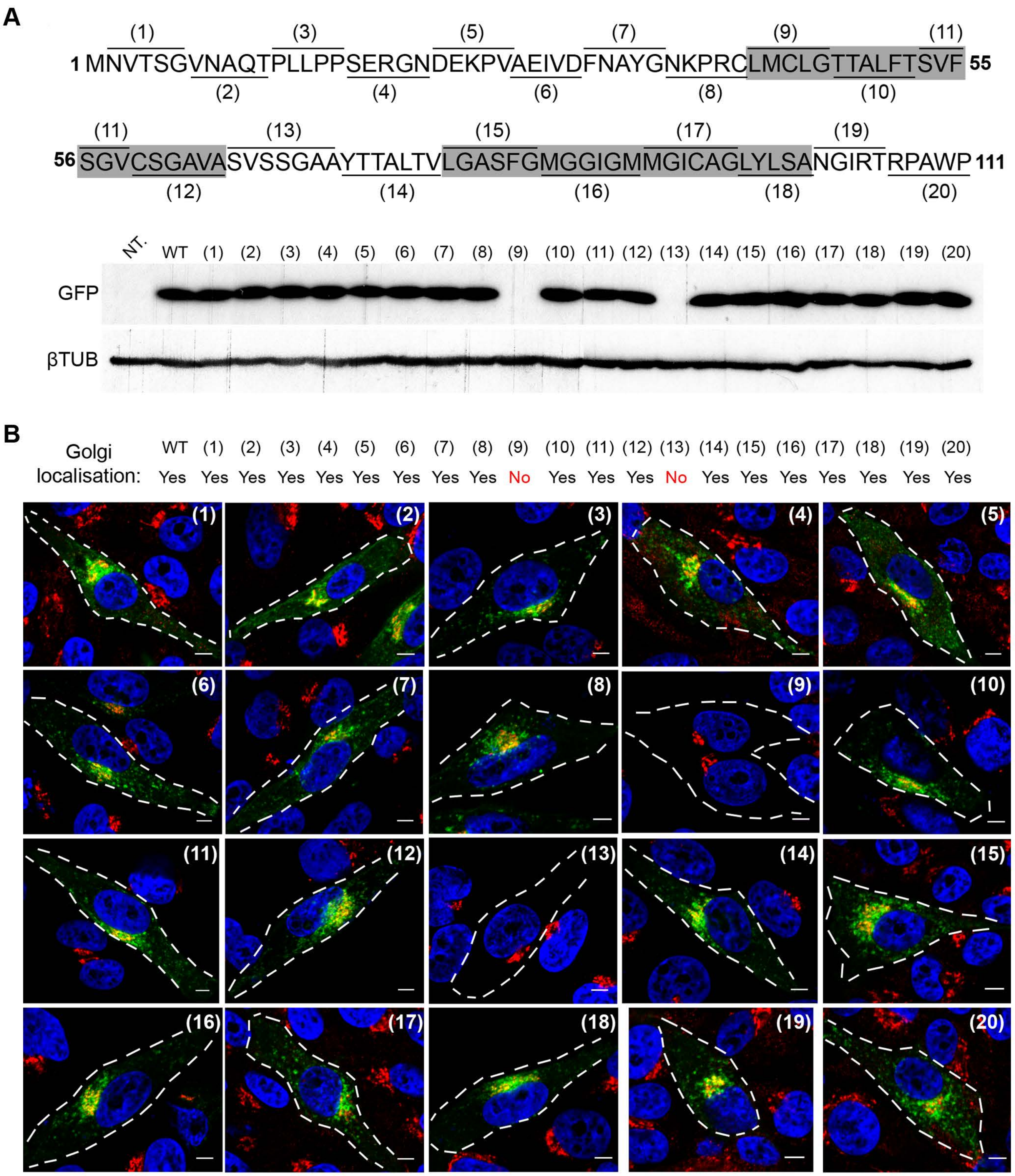

Figure S5, related to Figures 1, 3, 4 and 6

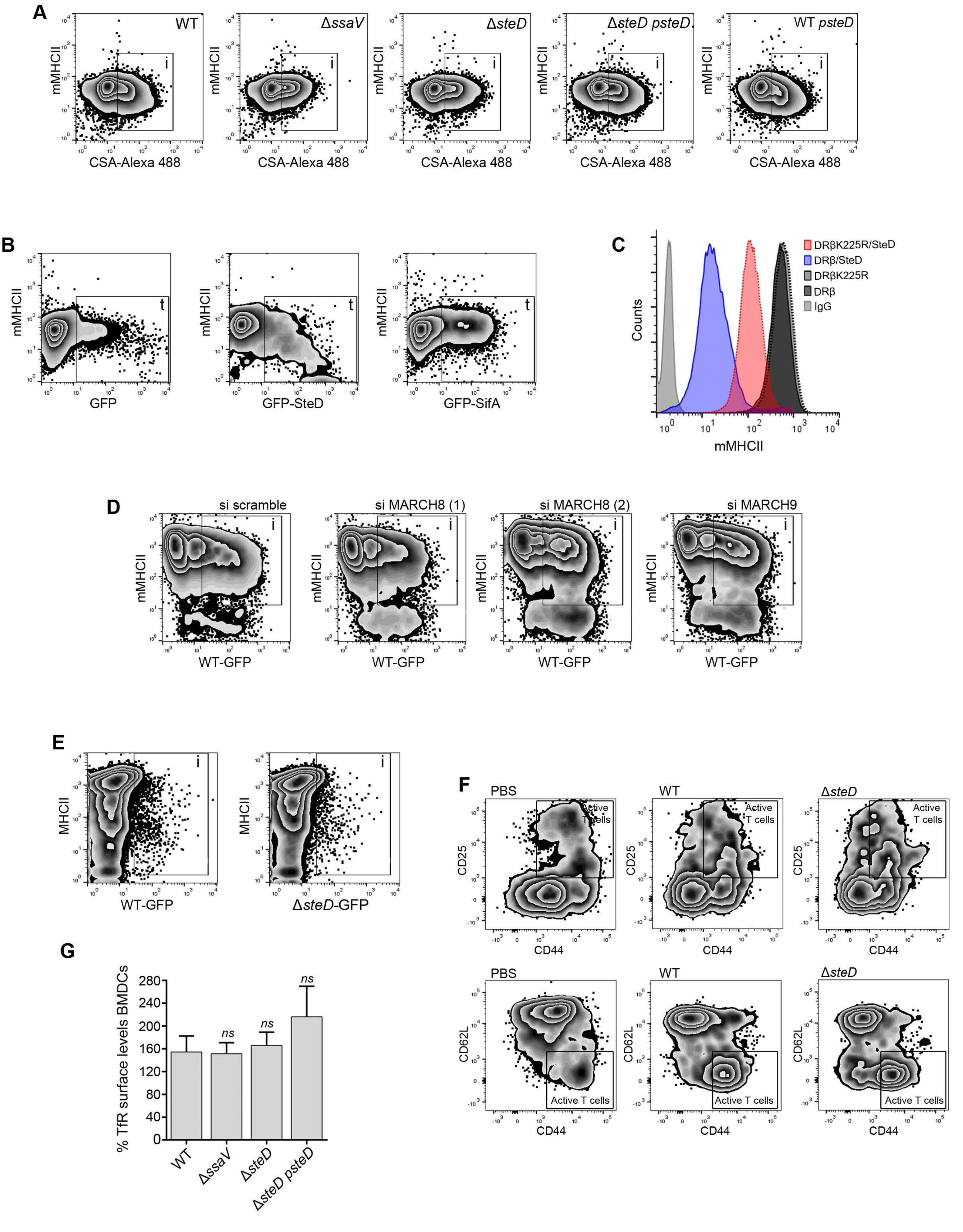

Legends to Supplemental Figures

Figure S1, related to Figure 2: *(A)* ClustalW alignment of amino acid sequence of SteD from different *Salmonella enterica* strains and serovars. Colours differentiate between amino acids with the same properties. *(B)* Confocal immunofluorescence microscopy of Mel JuSo cells at 20 h post-invasion with Δ*steD psteD* or post-transfection with M4P plasmid expressing GFP-SteD. Effector is shown in green (anti-HA or GFP) and TGN46 *trans*-Golgi marker is in red. Pearson's correlation coefficient of the Golgi region is shown on the right. **P<0.01 (Student's *t* test). *(C)* Confocal immunofluorescence microscopy of Mel JuSo cells at 20 h post-invasion by Δ*ssaV psteD-HA* or Δ*steD psteD-HA* strains of *S*. Typhimurium. Effector is shown in green (anti-HA) and *Salmonella* in red (anti-CSA-1). (D) Infected cells at 20 h post-invasion were incubated with Brefeldin A (BFA) for 90 min. Then, BFA was washed out and cells were incubated for another 90 min. Cells were fixed at different times and analyzed by immunofluorescence microscopy with anti-HA, anti-TGN46 and anti-GM130 antibodies. Scale bar - 5 µm. Quantification of the percentage of infected cells with a compact, perinuclear accumulation of SteD-2HA and TGN46 after exposure to BFA and wash out is shown on the right.

Figure S2, related to Figure 4: *(A)* Quantitative real-time PCR analysis showing mRNA levels of MARCH8 and MARCH9 in Mel JuSo cells after exposure to a scramble siRNA oligo, two oligos specific to MARCH8, or one oligo specific to MARCH9. *(B)* Quantitative real-time PCR analysis showing mRNA levels of MARCH8 in Mel JuSo cells after transduction with scramble or MARCH8 shRNAs. *(C,D)* Stable Mel JuSo cell lines expressing GFP-SteD were transfected with a vector expressing mCherry-MARCH8. After 20 h, cells were subjected to live imaging analysis by time lapse microscopy. Time points were extracted from Movie S2 showing vesicles positive for both GFP-SteD and mCherry-MARCH8 (arrowheads) displaying anterograde *(C)* and retrograde movement *(D)*. Light blue arrows indicate the direction of movement. Scale bar - 5 µm. Data in *(A)* were compared to si scramble by one-way ANOVA followed by Dunnett's multiple comparison test. ****P<0.0001, *ns* (not significant). Data in *(B)* were analysed by Student's *t* test. ****P<0.0001.

Figure S3, related to Figure 5: *(A)* Membrane fractionation of Mel JuSo cells infected for 20 h with Δ*steD psteD*^{Ct} or *(B)* stable cell lines expressing GFP-SteD^{6} or GFP-SteD^{16}. Soluble proteins were separated from total membrane proteins, which were later treated with 2.5 M urea to discriminate between integral membrane and peripheral membrane proteins by ultracentrifugation. Calreticulin (CALR) is a peripheral membrane protein, TGN46 is an integral *trans*-Golgi membrane protein and DRα is an integral membrane protein. *(C)* Mel JuSo cells were transfected with vector encoding SteD fused at its N-terminus to FLAG epitope (FLAG-SteD) (right panel) or to HA-tag at its C-terminus (left panel). Cells were semi- or completely permeabilised with digitonin or Triton X-100 to discriminate between cytoplasmic and Golgi luminal antigens, respectively. Antibodies recognising the luminal portion of TGN46 or cytoplasmic GM130 were used as controls. Scale bar - 5 µm.

Figure S4, related to Figure 5: *(A)* Schematic representation of blocks of SteD amino acids (over and underlined) that were substituted with alanines (top) and western blot analysis of Mel JuSo cells transfected with vectors expressing GFP-tagged mutated versions of SteD (bottom). WT, wild-type SteD. NT, non-transfected. Anti-GFP and anti-β tubulin antibodies were used. *(B)* Immunofluorescence microscopy of Mel JuSo cells 20 h after transfection with vectors expressing mutant versions of SteD. GFP-fusion proteins are in green and TGN46 labelling is in red. Nucleus is stained by DAPI in blue. Scale bar - 5 µm.

Figure S5, related to Figures 1, 3, 4 and 6: Representative flow cytometry zebra plots and histogram corresponding to fig. 1C. *(A)*, fig. 1D *(B)*, fig. 3F *(C)*, fig. 4A *(D)*, fig. 6E *(E)* and fig. 6F *(F)*. Boxed areas: i – infected; t – transfected. Mouse bone marrow-derived dendritic cells (BMDCs) were infected with the indicated bacterial strains and transferrin receptor (TfR) surface levels were quantified by flow cytometry at 20 h post-uptake. Surface levels of TfR in infected cells are represented as a percentage of those in uninfected cells from the same sample and are the means ± SD from three independent experiments. Data were compared to WT by one-way ANOVA followed by Dunnett's multiple comparison test, *ns* (not significant).

Movie S1, related to Figure 2: Stable Mel JuSo cell line expressing GFP-SteD.

Movie S2, related to Figure 4: Stable Mel JuSo cell line expressing GFP-SteD was transfected with vector expressing mCherry-MARCH8 and movie was acquired 20 h after transfection.

Table S1, related to Figures 1, 2, 3, 4, 5 and 6. Strains, plasmids and antibodies used in this study.

***S.* Typhimurium strains**

Name	Description	Reference
wild-type	12023 *S.* Typhimurium wild-type	NTCC
Δ*ssaV*	Δ*ssaV* ::km	Beuzon et al., 1999
Δ*sseL*	Δ*sseL* ::km	Mesquita et al., 2012
Δ*slrP*	Δ*slrP* ::km	Andreas Bäumler
Δ*sspH1*	Δ*sspH1* ::km	Figueira et al., 2013
Δ*sspH2*	Δ*sspH2* ::km	Figueira et al., 2013
Δ*gogB*	Δ*gogB* ::km	Figueira et al., 2013
Δ*sifA*	Δ*sifA* ::km	Beuzon et al., 2002
Δ*sifA* /Δ*sopD2*	Δ*sifA* ::km/Δ*sopD2*	Stéphane Méresse
Δ*sifB*	Δ*sifB* ::km	Figueira et al., 2013
Δ*sseJ*	Δ*sseJ* ::cm	Lossi et al., 2008
Δ*sseF*	Δ*sseF* ::km	Brumell et al., 2003
Δ*sseG*	Δ*sseG* ::km	Brumell et al., 2003
Δ*sopD2*	Δ*sopD2* ::cm	Figueira et al., 2013
Δ*pipB*	Δ*pipB* ::km	Figueira et al., 2013
Δ*pipB2*	Δ*pipB2* ::km	Figueira et al., 2013
Δ*sopD*	Δ*sopD* ::km	Figueira et al., 2013
Δ*srfJ*	Δ*srfJ* ::km	Figueira et al., 2013
Δ*srfH*	Δ*srfH* ::km	Figueira et al., 2013
Δ*sptP*	Δ*sptP* ::km	Figueira et al., 2013
Δ*steA*	Δ*steA* ::km	Figueira et al., 2013
Δ*steB*	Δ*steB* ::km	Figueira et al., 2013
Δ*steC*	Δ*steC* ::km	Mesquita et al., 2012
Δ*steD*	Δ*steD* ::km	This study
Δ*steE*	Δ*steE* ::km	This study
Δ*spvB*	Δ*spvB* ::km	Figueira et al., 2013
Δ*spvC*	Δ*spvC*	Figueira et al., 2013
Δ*spvD*	Δ*spvD* ::km	Figueira et al., 2013
Δ*sseK1*	Δ*sseK1* ::km	Figueira et al., 2013
Δ*sseK2*	Δ*sseK2* ::km	Figueira et al., 2013
Δ*sseK3*	Δ*sseK3* ::km	Figueira et al., 2013
Δ*sseK1* /Δ*sseK2* /Δ*sseK3*	Δ*sseK1* /Δ*sseK2* /Δ*sseK3* ::km	Figueira et al., 2013
Δ*cigR*	Δ*cigR* ::km	Figueira et al., 2013
Δ*gtgA*	Δ*gtgA* ::km	Figueira et al., 2013
Δ*gtgE*	Δ*gtgE* ::km	Figueira et al., 2013
Δ*avrA*	Δ*avrA* ::km	Figueira et al., 2013
wild-type SL3261	SL3261 (*aroA*)	Hoiseth et al., 1981
Δ*steD* SL3261	SL3261 (*aroA* /Δ*steD*)	This study.

Plasmids

Name	Description	Reference
pKD4	PCR template plasmid with Km resistance cassette (KmR)	Datsenko et al., 2000
pKD46	Plasmid encoding arabinose-inducible λ-Red recombinase system (CarbR)	Datsenko et al., 2000
pCP20	Plasmid encoding arabinose-inducible FLP recombinase (CarbR)	Datsenko et al., 2000
pFCcGi	rpsM::mCherry and PBAD::gfpmut3a promoter fusions in pFPV25.1 (CarbR)	Figueira et al., 2013
pFPV25.1	rpsM::gfpmut3a promoter fusion in pFPV25	Valdivia et al., 1996

pMD.GAGPOL	Retroviral helper plasmid encoding gag/pol proteins	Randow and Sale, 2006
pMD.VSVG	Retroviral helper plasmid encoding vesicular stomatitis virus glycoproteins	Randow and Sale, 2006
psteD	pWSK29 containing C-terminus 2HA-tagged steD-promoter and open reading frame (CarbR)	This study
GFP	M5P retroviral vector containing GFP open reading frame (CarbR/PuroR)	Teresa Thurston
GFP-SteD	M5P retroviral vector containing N-terminus GFP-tagged steD open reading frame (CarbR)	This study
GFP-SifA	M5P retroviral vector containing N-terminus GFP-tagged sifA open reading frame (CarbR)	McGourty et al., 2013
GFP-SseF	M5P retroviral vector containing N-terminus GFP-tagged sseF open reading frame (CarbR)	Teresa Thurston
GFP-SseG	M5P retroviral vector containing N-terminus GFP-tagged sseG open reading frame (CarbR)	Teresa Thurston
HA-DRβ	M5P retroviral vector containing N-terminus HA-tagged HLA-DR beta chain (CarbR/PuroR)	This study
HA-DRβ-K225R	M5P retroviral vector containing N-terminus HA-tagged HLA-DR beta chain with K225 mutated (CarbR/PuroR)	This study
MARCH8-FLAG	M5P retroviral vector containing C-terminus FLAG-tagged MARCH8 (CarbR/PuroR)	This study

Antibodies

Specificity	Clones	Use	Source
HLA-DR	L243	FACS/IP	Sigma-Aldrich
HLA-DRα	TAL.1B5	WB	Dako
HLA-DRβ	DA2	WB	Abcam
HA	3F10	IF	Roche
FLAG	M2	IF	Sigma-Aldrich
TGN46	Rabbit Polyclonal	IF/WB	LifeSpan Biosciences
GM130	35	IF	BD Biosciences
Golgin 97	CDF4	WB	Invitrogen
Calreticulin	Rabbit Polyclonal	WB	Thermo Scientific
CSA-1	Goat polyclonal	IF	KPL
Ubiquitin-HRP	P4D1	WB	Santa Cruz
Tubulin β	Rabbit monoclonal	WB	Abcam
HA	Rabbit Polyclonal	WB	Sigma-Aldrich
FLAG	Rabbit Polyclonal	WB	Sigma-Aldrich
GFP	Rabbit monoclonal	WB	Thermo Scientific
CD11c	N418	FACS	MiltenyiBiotec
TfR	H68.4	FACS	Zymed Laboratories
CD3ε	145-2C11	FACS	MiltenyiBiotec
CD4	RM4-5	FACS	BD Pharmingen
CD25	7D4	FACS	MiltenyiBiotec
CD44	IM7.8.1	FACS	MiltenyiBiotec
CD62L	MEL-14	FACS	MiltenyiBiotec
B7.2	PO3.3	FACS	MiltenyiBiotec
I-A/I-E	M5/114.15.2	FACS	Thermo Scientific

WB: western blot; FACS: flow cytometer; IP: immunoprecipitation; IF: immunofluorescence.

Table S2, related to Figure 6. Bacterial load (c.f.u.) per spleen of infected mice.

Group compared	WT	Δ*steD*
1	112000	124000
2	98000	96250
3	87500	94750
4	86000	84500
5	68750	70000
6	67300	64550
7	57000	60450
8	54275	54900
9	53150	54566
10	44700	45475
11	44825	47800
12	39000	36733

Supplemental Experimental Procedures

siRNA and shRNA transfection

Mel JuSo cells were seeded in 6-well plates and transfected on the following day with siRNA oligonucleotides using Lipofectamine RNAiMAX^{TM} (Life technologies) according to the manufacturer's instructions with a final concentration of 50 nM. Cells were reseeded 24 h after the first transfection and 24 h later a second round of transfection was done. Cells were infected 24 h after the second transfection and analysed 16-20 h post-invasion. siRNA oligonucleotides for MARCH8 (5'-TCCAGCGGGATTGACACTCAA, CTGCTAGAGTCTACAGAAGTA-3') and MARCH9 (5'-CAGGTTGGATGCCGTTGCAGA, CAGCACTCCGAGGTATCTAAA-3') were from Qiagen. A scrambled sequence (5'-AAACTTGTCGACGAGAAGCAA-3') was included in all experiments as a negative control. For shRNA construction, siRNA sequences for MARCH8 (5'-TCCAGCGGGATTGACACTCAA) and scramble (5'-AAACTTGTCGACGAGAAGCAA-3') were cloned into pSUPER vector.

Immunofluorescence microscopy

Cells were seeded onto coverslips and infected as described in the Experimental Procedures. For general procedures, cells were collected at 20 h post-invasion, washed with PBS and fixed in 3% paraformaldehyde in PBS for 10 min at room temperature. Cells were incubated with 50 mM NH_4Cl for 10 min, washed and labelled with appropriate antibodies diluted in 10% FCS and 0.1% saponin in PBS. Antibodies used for IF are listed in Table S1. For Brefeldin A (BFA) treatment, at 20 h post-invasion new medium containing 5 µg/ml of BFA (Sigma-Aldrich) was added to infected cells and incubated for 90 min. The washout was done by washing cells 5 times in PBS and incubating them in 10% DMEM for another 90 min prior to final wash and fixation for labelling. For selective permealisation, digitonin treatment was done using live cells. At 20 h post-invasion, cells were placed on ice, washed with KHM buffer (110 mM KOAc, 20 mM HEPES, 2 mM $MgCl_2$, pH 7.3) and incubated for 5 min with 40 µg/ml digitonin diluted in KHM. Cells were washed and incubated with primary antibodies diluted in 10% FCS in PBS for 30 min on ice. After washes, cells were fixed and incubated with secondary antibodies at room temperature under standard procedures. After fixation, cells were incubated with 0.1% Triton X-100 for 5 min at room temperature prior to labelling for 1 h with primary and then secondary antibodies diluted in 10% FCS and 0.1% saponin in PBS. For visualization of internalised mMHCII complexes, cells were placed on ice and incubated with mAb L243

diluted in DMEM containing 10% FCS for 30 min. Cells were washed, incubated with warm medium and returned to the incubator at 37 °C for another 4 h. After incubation, cells were washed, fixed and labelled with secondary antibody diluted in 10% FCS and 0.1% saponin in PBS.

Immunoprecipitation and immunoblots

For immunoprecipitation and immunoblotting, approximately 1 x 10^7 cells were harvested using Cell Dissociation Buffer (Sigma) and lysed in buffer containing 1% Nonidet P-40, 50 mM Tris-Cl (pH 7.4), 5 mM EDTA, 150 mM NaCl, protease inhibitors (Roche) and 10 mM iodoacetamide (IAA) for 30 min at 4°C. Lysate was centrifuged at 16,000 *g* for 15 min and post-nuclear supernatant was incubated with 30 µl of slurry solution containing CNBr sepharose-coupled mAb L243 or isotype control for 2 h at 4°C. Immunoprecipitates were washed with lysis buffer and eluted with 100 mM glycine (pH 3.0). Eluates were precipitated with four volumes of cold acetone, resuspended in SDS-PAGE loading buffer and analysed by western blot. For infected cells, the MOI was increased to 300:1 and infection time to 45 min. For co-immunoprecipitations, cells were lysed with 50 mM Tris-Cl (pH 7.4), 150 mM NaCl, 5 mM EDTA, 5% glycerol, 10 mM IAA and 0.5% Triton X-100 containing protease inhibitors for 15 min at 4°C. Lysate was centrifuged at 16,000 *g* for 15 min and post-nuclear supernatant was incubated with GFP-Trap (ChromoTek) or CNBr sepharose-coupled mAb L243 or isotype control for 2 h at 4°C. Immunoprecipitates were washed with lysis buffer and eluted with glycine as described above. Protein samples were separated by SDS-PAGE and transferred to Immobilon-P™ membrane (Millipore). Membranes were blocked in 5% skimmed milk and 0.1% Tween-20 in PBS and incubated with primary and secondary antibodies in the same solution, which were detected using ECL Plus™ Western Blotting Detection Reagents (Thermo Fisher). Band intensities were calculated using Image J software.

Supplemental References

Beuzon, C.R., Meresse, S., Unsworth, K.E., Ruiz-Albert, J., Garvis, S., Waterman, S.R., Ryder, T.A., Boucrot, E., and Holden, D.W. (2000). *Salmonella* maintains the integrity of its intracellular vacuole through the action of SifA. The EMBO journal *19*, 3235-3249.

Brumell, J.H., Kujat-Choy, S., Brown, N.F., Vallance, B.A., Knodler, L.A., and Finlay, B.B. (2003). SopD2 is a novel type III secreted effector of *Salmonella typhimurium* that targets late endocytic compartments upon delivery into host cells. Traffic *4*, 36-48.

Figueira, R., Watson, K.G., Holden, D.W., and Helaine, S. (2013). Identification of salmonella pathogenicity island-2 type III secretion system effectors involved in intramacrophage replication of *S. enterica* serovar typhimurium: implications for rational vaccine design. mBio *4*, e00065.

Hoiseth, S.K. and Stocker, B.A. (1981). Aromatic-dependent *Salmonella typhimurium* are non-virulent and effective as live vaccines. Nature 291, 238-239.

Lossi, N.S., Rolhion, N., Magee, A.I., Boyle, C., and Holden, D.W. (2008). The *Salmonella* SPI-2 effector SseJ exhibits eukaryotic activator-dependent phospholipase A and glycerophospholipid: cholesterol acyltransferase activity. Microbiology *154*, 2680-2688.

McGourty, K., Thurston, T.L., Matthews, S.A., Pinaud, L., Mota, L.J., and Holden, D.W. (2012). *Salmonella* inhibits retrograde trafficking of mannose-6-phosphate receptors and lysosome function. Science *338*, 963-967.

Mesquita, F.S., Thomas, M., Sachse, M., Santos, A.J., Figueira, R., and Holden, D.W. (2012). The *Salmonella* deubiquitinase SseL inhibits selective autophagy of cytosolic aggregates. PLoS pathogens *8*, e1002743.

Valdivia, R.H., and Falkow, S. (1996). Bacterial genetics by flow cytometry: rapid isolation of *Salmonella typhimurium* acid-inducible promoters by differential fluorescence induction. Molecular microbiology *22*, 367-378.
