# Supplementary figures and images for: The Salmonella Effector SteD Mediates MARCH8-Dependent Ubiquitination of MHC II Molecules and Inhibits T Cell Activation
Source: Cell Host Microbe. 2016 Nov 9;20(5):584–95. doi: 10.1016/j.chom.2016.10.007 (PMC5104694; doi:10.1016/j.chom.2016.10.007)

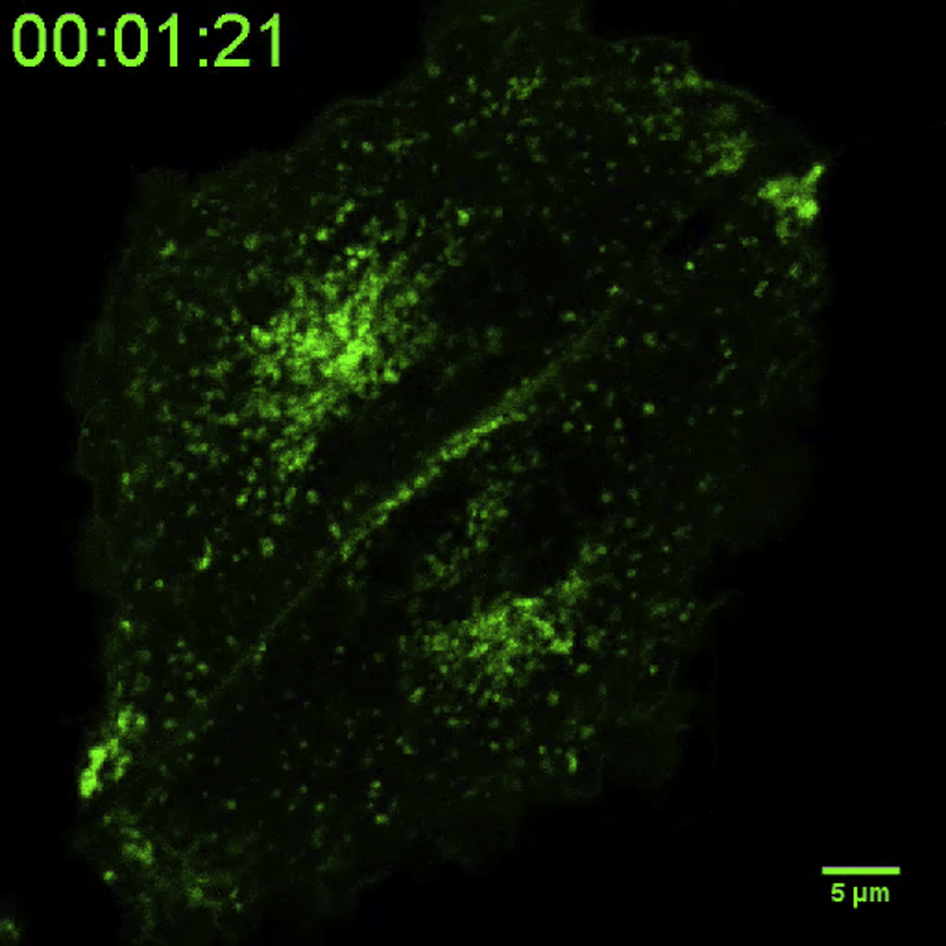

Supplement: Movie S1. Stable Mel Juso Cell Line Expressing GFP-SteD, Related to Figure 2 [file mmc2.jpg]

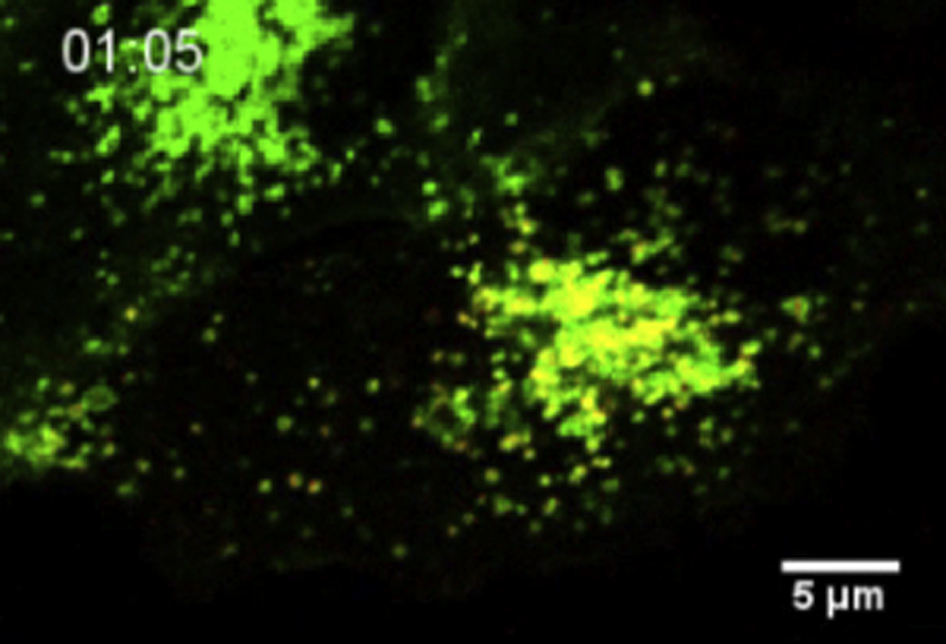

Supplement: Movie S2. Stable Mel Juso Cell Line Expressing GFP-SteD Was Transfected with Vector Expressing mCherry-MARCH8 and Movie Was Acquired 20 hr after Transfection, Related to Figure 4 [file mmc3.jpg]
