# Supplementary material for: The Salmonella Effector SteD Mediates MARCH8-Dependent Ubiquitination of MHC II Molecules and Inhibits T Cell Activation
Source: Cell Host Microbe. 2016 Nov 9;20(5):584–95. doi: 10.1016/j.chom.2016.10.007 (PMC5104694; doi:10.1016/j.chom.2016.10.007)
Supplement: Document S2. Article plus Supplemental Information [file mmc4.pdf]

Article

Cell Host & Microbe

The *Salmonella* Effector SteD Mediates MARCH8-Dependent Ubiquitination of MHC II Molecules and Inhibits T Cell Activation

Graphical Abstract

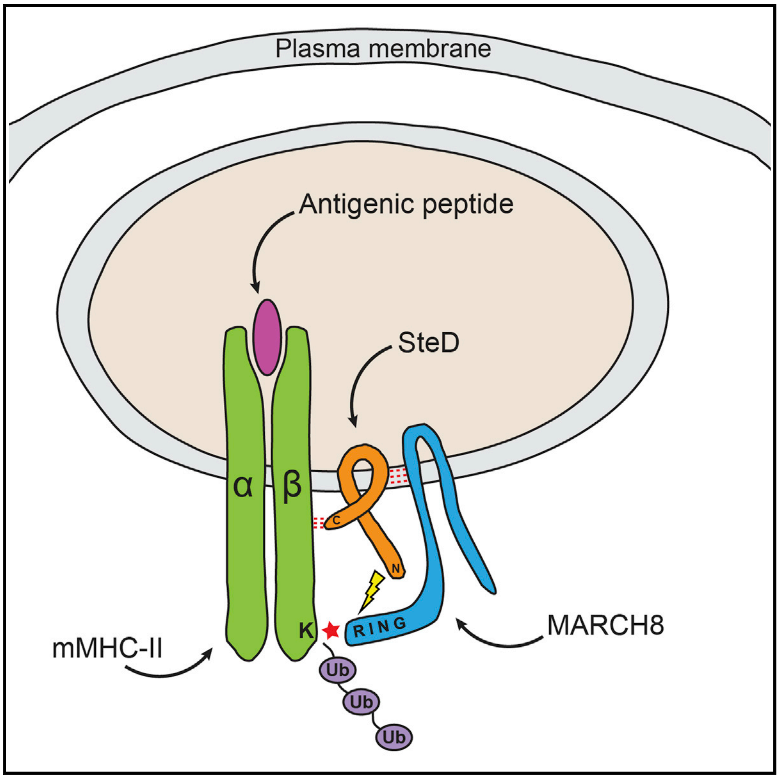

Authors

Ethel Bayer-Santos,
Charlotte H. Durkin,
Luciano A. Rigano, ...,
Nicolas Lapaque,
Stefan H.E. Kaufmann,
David W. Holden

Correspondence

d.holden@imperial.ac.uk

In Brief

Dendritic cell infection by *Salmonella* depletes mature MHC class II (mMHCII) from the cell surface. Bayer-Santos et al. reveal that the *Salmonella* effector SteD binds the E3 ligase MARCH8 and mMHCII to promote mMHCII ubiquitination and surface depletion. SteD suppressed dendritic cell-mediated T cell activation in vitro and in mice.

Highlights

- *Salmonella* effector SteD promotes ubiquitination and surface depletion of mature MHCII
- SteD binds both MHCII and the host E3 ubiquitin ligase MARCH8
- SteD uses MARCH8 to ubiquitinate and surface deplete MHCII
- SteD suppresses T cell activation during *Salmonella* infection in vitro and in mice

Bayer-Santos et al., 2016, Cell Host & Microbe *20*, 584–595
November 9, 2016 © 2016 The Authors. Published by Elsevier Inc.
http://dx.doi.org/10.1016/j.chom.2016.10.007

Cell Host & Microbe
Article

The *Salmonella* Effector SteD Mediates MARCH8-Dependent Ubiquitination of MHC II Molecules and Inhibits T Cell Activation

Ethel Bayer-Santos,[1,6] Charlotte H. Durkin,[1,6] Luciano A. Rigano,[1] Andreas Kupz,[2,5] Eric Alix,[1] Ondrej Cerny,[1] Elliott Jennings,[1] Mei Liu,[1] Aindrias S. Ryan,[1] Nicolas Lapaque,[3,4] Stefan H.E. Kaufmann,[2] and David W. Holden[1,7,*]
[1]MRC Centre for Molecular Bacteriology and Infection, Imperial College London, Armstrong Road, London SW7 2AZ, UK
[2]Department of Immunology, Max Planck Institute for Infection Biology, 10117 Berlin, Germany
[3]INRA, UMR 1319 Micalis, Domaine de Vilvert, Jouy-en-Josas 78352, France
[4]AgroParisTech, UMR Micalis, Jouy-en-Josas 78350, France
[5]Centre for Biosecurity and Tropical Infectious Diseases, Australian Institute of Tropical Health and Medicine, James Cook University, McGregor Road, Cairns, QLD 4878, Australia
[6]Co-first author
[7]Lead Contact
*Correspondence: d.holden@imperial.ac.uk
http://dx.doi.org/10.1016/j.chom.2016.10.007

SUMMARY

The SPI-2 type III secretion system (T3SS) of intracellular *Salmonella enterica* translocates effector proteins into mammalian cells. Infection of antigen-presenting cells results in SPI-2 T3SS-dependent ubiquitination and reduction of surface-localized mature MHC class II (mMHCII). We identify the effector SteD as required and sufficient for this process. In Mel Juso cells, SteD localized to the Golgi network and vesicles containing the E3 ubiquitin ligase MARCH8 and mMHCII. SteD caused MARCH8-dependent ubiquitination and depletion of surface mMHCII. One of two transmembrane domains and the C-terminal cytoplasmic region of SteD mediated binding to MARCH8 and mMHCII, respectively. Infection of dendritic cells resulted in SteD-dependent depletion of surface MHCII, the co-stimulatory molecule B7.2, and suppression of T cell activation. SteD also accounted for suppression of T cell activation during *Salmonella* infection of mice. We propose that SteD is an adaptor, forcing inappropriate ubiquitination of mMHCII by MARCH8 and thereby suppressing T cell activation.

INTRODUCTION

Major histocompatibility complex class II (MHCII) molecules have a pivotal function in adaptive immunity by displaying antigenic peptides on the surface of antigen-presenting cells such as dendritic cells (DCs) to CD4-restricted T cells, leading to their activation, proliferation, and differentiation. MHCII complexes consist of heterodimers of α and β chains that assemble in the endoplasmic reticulum (ER) along with the chaperone invariant chain (Ii) to form a larger structure called the immature MHCII complex (Ii-MHCII). This complex migrates through the Golgi network to the plasma membrane and a late endosomal MHCII compartment, where Ii is degraded by lysosomal proteases, leaving the CLIP peptide in the MHCII peptide-binding groove of the α/β heterodimer. Class II related chaperones HLA-DM and HLA-DO exchange CLIP for antigenic peptides to form mature MHCII (mMHCII) complexes. These are transported to the cell surface for antigen presentation (Neefjes et al., 2011). Surface-localized mMHCII can undergo internalization and recycling back to the plasma membrane (Roche and Furuta, 2015). In immature DCs, the E3 ligase MARCH1 ubiquitinates internalized mMHCII, leading to its incorporation into multivesicular bodies and lysosomal degradation (Cho et al., 2015). Activation of DCs is accompanied by reduced expression of MARCH1, enabling internalized mMHCII to recycle back to the plasma membrane (De Gassart et al., 2008).

After oral ingestion, *Salmonella enterica* encounters DCs in Peyer's patches of the small intestine (Tam et al., 2008). Following uptake by DCs, the majority of bacteria remain within a membrane bound compartment, the *Salmonella*-containing vacuole (SCV), from where they deliver effector proteins to the host cell via the SPI-2 type III secretion system (T3SS) (Jantsch et al., 2003).

It has been known for over a decade that *Salmonella* inhibits the process of antigen presentation by mMHCII molecules in DCs (Cheminay et al., 2005; Halici et al., 2008; Jackson et al., 2013; Lapaque et al., 2009a; Mitchell et al., 2004; Tobar et al., 2004, 2006). This is dependent on a functional SPI-2 T3SS (Cheminay et al., 2005; Mitchell et al., 2004). Mutant strain analysis showed that several effectors affecting vesicular trafficking disrupt T cell proliferation (Cheminay et al., 2005; Halici et al., 2008). Another study revealed that in *Salmonella*-infected cells, surface levels of mMHCII are reduced through its ubiquitination (Jackson et al., 2013; Lapaque et al., 2009a). However, the effector(s) and mechanisms involved in the regulation of mMHCII ubiquitination are unknown.

Here, we show that SteD is a SPI-2 T3SS effector that is required and sufficient for depletion of mMHCII from the surface of infected cells. Further work showed that SteD

 © 2016 The Authors. Published by Elsevier Inc.
This is an open access article under the CC BY license (http://creativecommons.org/licenses/by/4.0/).

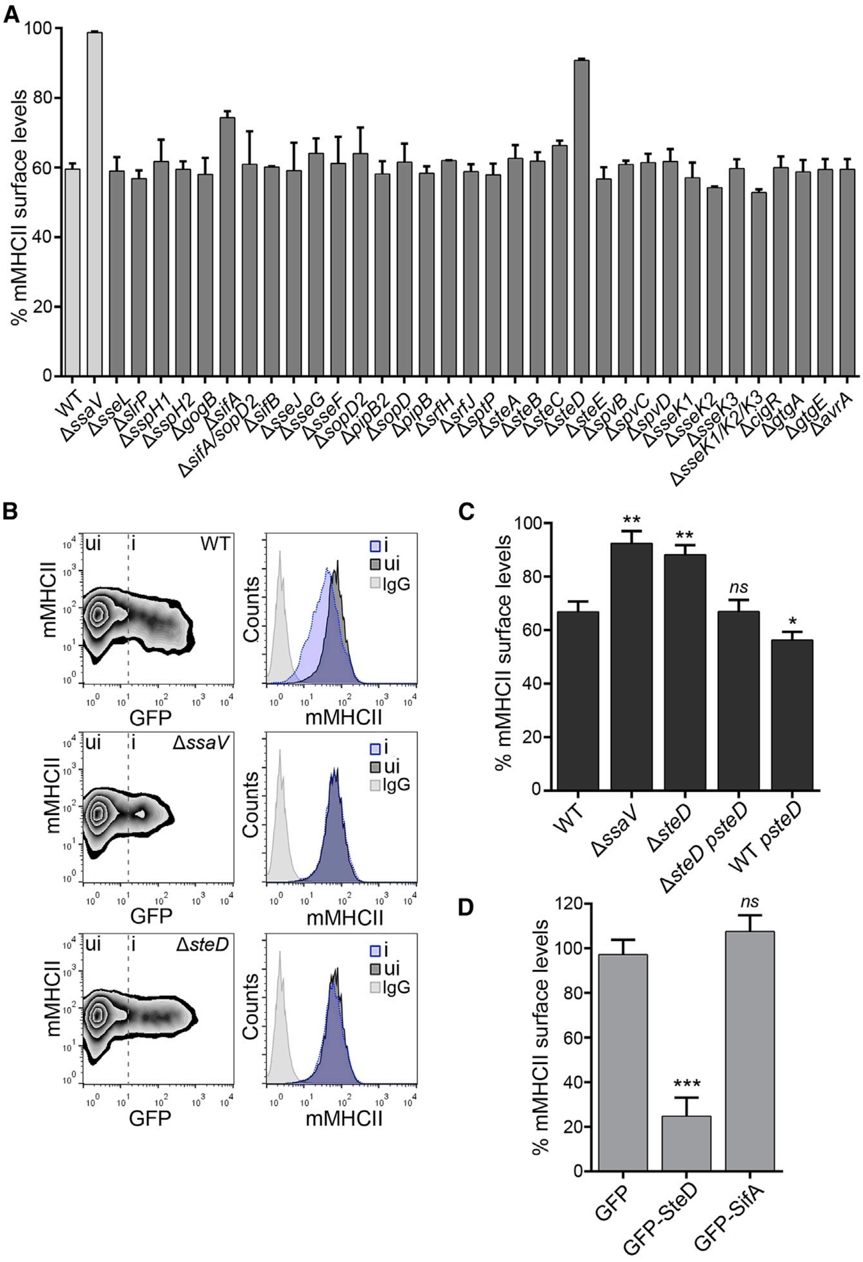

Figure 1. *Salmonella* SPI-2 T3SS Effector SteD Reduces Surface Levels of Mature MHCII Molecules

(A) Mel Juso cells were infected with WT or mutant *Salmonella* strains for 16 hr and surface levels of mMHCII were measured by flow cytometry using mAb L243 (that specifically recognizes mature HLA-DR). The error bars represent SD of the geometric mean fluorescence of two independent experiments performed in duplicate.

(B) Representative FACS plots showing surface levels of mMHCII in infected cells (i) compared to uninfected cells (ui). The histograms show surface levels of mMHCII in infected (i, blue) and uninfected (ui, dark gray) cells. The cells labeled with isotype control antibody are shown in light gray.

(C) Mel Juso cells were infected with Δ*steD* strain carrying pWSK29, expressing SteD-2HA regulated by its endogenous promoter (*psteD*). The infected and uninfected cells were discriminated using anti-*Salmonella* CSA-1 antibody after fixation. The data represent surface levels of mMHCII in infected cells as a percentage of those in uninfected cells from the same sample.

(D) Mel Juso cells were transfected with vectors encoding GFP-tagged effectors, and mMHCII was analyzed by flow cytometry. The data represent surface levels of mMHCII in transfected cells as a percentage of those in untransfected cells from the same sample.

(C and D) Error bars represent SD of the geometric mean fluorescence of three experiments done in duplicate and were analyzed through comparison with WT *Salmonella* (C) or GFP-only transfected cells (D) by one-way ANOVA followed by Dunnett's multiple comparison test. ***$p < 0.001$, **$p < 0.01$, *$p < 0.05$, and not significant, *ns*.

is an integral membrane protein that interacts with the E3 ubiquitin ligase MARCH8 and mMHCII. SteD stimulates MARCH8-mediated ubiquitination of the β chain of mMHCII and accounts for the ability of *Salmonella* to inhibit T cell responses.

RESULTS

SteD Reduces Surface Levels of mMHCII

To identify *Salmonella* SPI-2 T3SS effector(s) involved in the removal of mMHCII molecules from the surface of infected cells, we used a collection of mCherry-expressing mutant strains lacking individual SPI-2 T3SS effectors to infect human Mel Juso cells. This cell line is widely used to study MHC class II trafficking and presentation. Three human MHCII isotypes exist: HLA-DR, HLA-DQ, and HLA-DP. mMHCII surface levels were measured by flow cytometry using mAb L243, which recognizes mature HLA-DR (Bijlmakers et al., 1994). Of the panel of 33 single mutants, a double, and a triple mutant, all strains reduced surface mMHCII to approximately the same degree as the wild-type (WT) strain, with the exception of Δ*ssaV*, Δ*sifA*, and Δ*steD* strains (Figure 1A). SsaV is an essential component of the SPI-2 secretion apparatus, and its absence prevents bacteria from translocating all T3SS effectors. Vacuoles harboring Δ*sifA* bacteria are unstable, whereas the majority of vacuoles containing Δ*sifA*/Δ*sopD2* bacteria remain intact (Schroeder et al., 2010). The surface levels of mMHCII in cells infected with the Δ*sifA*/Δ*sopD2* mutant were similar to those caused by the WT strain, suggesting that the effect of the Δ*sifA* mutant is likely to be indirect, resulting from loss of the vacuolar membrane. We created a second *steD* deletion mutant expressing GFP and tested its effect on surface levels of mMHCII in infected Mel Juso cells. There was a reduction of mMHCII in cells infected with GFP-expressing WT bacteria (Figure 1B, i) compared to uninfected cells (Figure 1B, ui), but

no difference was detected in Δ*ssaV* or Δ*steD* infected cells (Figure 1B, i) compared to uninfected cells in the same sample (Figure 1B, ui). To establish if the lack of effect of Δ*steD* on mMHCII was due to the absence of *steD* and not to an adventitious mutation or polar effect, the mutant strain was transformed with a low copy number plasmid (pWSK29) encoding SteD-2HA under the control of its endogenous promoter. This strain (Δ*steD psteD*) removed surface mMHCII to the same extent as the WT strain (Figures 1C and S5A). The WT strain carrying *psteD-2HA* further reduced mMHCII surface levels (Figure 1C). The similar phenotypes of the Δ*steD* and Δ*ssaV* mutants suggest that SteD accounts for all of the SPI-2 T3SS-mediated effect. Furthermore, ectopic expression of GFP-tagged SteD or SifA in Mel Juso cells showed that SteD specifically reduced mMHCII from the cell surface in the absence of other SPI-2 effectors (Figures 1D and S5B). From these experiments, we conclude that SteD is required and sufficient for the reduction of surface levels of mMHCII in Mel Juso cells.

Localization and Topology of SteD

SPI-2 T3SS-dependent translocation of SteD was shown using CyaA reporter fusions (Niemann et al., 2011). *steD* is present in the genome of several serovars of *S. enterica*, including *S.* Typhimurium, *S.* Enteritidis, *S.* Gallinarum, *S.* Paratyphi, and *S.* Typhi (Figure S1A). No similarity of predicted amino acid sequences to proteins of any other bacterial genera was found by BLAST analysis.

To determine the subcellular localization of SteD after its translocation or ectopic expression, we infected Mel Juso cells with *Salmonella* Δ*steD psteD-2HA* or transfected cells with a plasmid encoding GFP-SteD. Immunofluorescence microscopy revealed that SteD accumulated in the region of the Golgi network, as shown by co-localization with the *trans*-Golgi network (TGN) protein TGN46 (Figure S1B). Translocation of SteD-2HA from bacteria depended on a functional SPI-2 T3SS (Figure S1C). To test if SteD localizes to Golgi membranes, Mel Juso cells were exposed to Brefeldin A (BFA) to disassemble the Golgi network, and the localization of SteD was examined by immunofluorescence microscopy together with *cis*- and TGN proteins GM130 and TGN46, respectively (Figure S1D). Along with TGN46 and GM130, SteD dispersed after exposure of cells to BFA. Furthermore, SteD relocalized to a reformed Golgi network 90 min after BFA washout (Figure S1D). There was significantly greater co-localization between SteD and TGN46 than with GM130 (Figure S1B, right), indicating that it accumulates predominantly in the TGN. Live cell imaging analysis of Mel Juso cells expressing GFP-SteD revealed that the majority of effector remained clustered and relatively immobile (consistent with TGN localization). However, a second pool of SteD was also observed in tubular/vesicular structures that were highly dynamic, moving extensively throughout cells in anterograde and retrograde directions (Movie S1).

SteD is a small protein comprising 111 amino acids (~12 kDa) and is predicted by TMHMM v2.0 software (Krogh et al., 2001) to be an integral membrane protein (Figure 2A). To test this experimentally, Mel Juso cells were infected with Δ*steD psteD-2HA* *Salmonella* and subjected to membrane fractionation. Using calreticulin (CALR) and Golgin 97 as controls for peripheral membrane proteins and TGN46 as an integral membrane protein, western blot analysis revealed that SteD was highly enriched in the fraction comprising transmembrane proteins (Figure 2B). To establish the topology of SteD, we used C-terminal HA-tagged effector translocated by the bacteria (Δ*steD psteD-2HA*) and N-terminal FLAG-tagged effector (FLAG-SteD) ectopically expressed in Mel Juso cells in membrane permeabilization experiments. Both N- and C-terminal epitope tags were detected after selective permeabilization of the plasma membrane with digitonin (Figure 2C), showing that SteD has two transmembrane regions and that both the N- and C-termini are exposed to the host cell cytosol (Figure 2D).

SteD Reduces Surface Levels of mMHCII by Increasing Ubiquitination of Residue K225 on the Cytoplasmic Tail of the β Chain

To determine if SteD affects the internalization and recycling process of surface-localized mMHCII, Mel Juso cells were infected with WT or Δ*steD* mutant bacteria and then incubated with unconjugated mAb L243 on ice for 30 min to label surface mMHCII. Subsequently, cells were washed and transferred to 37°C to reinitiate internalization/recycling and the amount of mMHCII remaining on the surface at different time points was analyzed by incubating cells with an Alexa Fluor-conjugated secondary antibody to mAb L243 and flow cytometry. In agreement with previous results (Lapaque et al., 2009a), WT *Salmonella*-infected cells underwent a greater rate of loss of surface mMHCII compared to uninfected cells; however, this difference was not observed when cells were infected with the Δ*steD* strain (Figure 3A). Confocal immunofluorescence microscopy of cells at the 4 hr time point revealed intracellular mMHCII in cells infected by WT *Salmonella* and Δ*steD psteD* strains, but not in cells infected by the Δ*steD* mutant (Figure 3B, Y-Z planes). These results show that SteD affects cell surface internalization and/or recycling of mMHCII.

Salmonella induces ubiquitination of a lysine residue (K225) in the cytosolic tail of the DRβ chain, and K225 is required for reduction of surface levels of mMHCII by *Salmonella* (Lapaque et al., 2009a). To investigate the involvement of SteD in this process, ubiquitination of mMHCII in infected cells and in cells stably expressing GFP or GFP-SteD was examined by immunoprecipitation of mMHCII and immunoblotting. Immunoprecipitates from cells infected by WT or Δ*steD psteD* strains contained ubiquitinated protein(s) whose levels were reduced or absent in cells infected by Δ*ssaV* and Δ*steD* strains (Figure 3C). Stable expression of GFP-SteD in Mel Juso cells also led to ubiquitination of immunoprecipitated protein(s) (Figure 3D). An anti-HA antibody was then employed to immunoprecipitate HA-tagged DRβ (either wild-type [HA-DRβ] or a mutant in which K225 was substituted with arginine [HA-DRβ K225R]) from Mel Juso cells stably expressing these constructs. There was an increase in ubiquitination of immunoprecipitated protein(s) in cells expressing GFP-SteD and HA-DRβ (DRβ/SteD) compared to cells lacking SteD (DRβ) (Figure 3E). The size of the most prominent band corresponds to di-ubiquitinated DRβ. No such SteD-dependent increase in ubiquitinated proteins was detected in immunoprecipitates from cells expressing HA-DRβ K225R, indicating that K225 is the target of SteD-induced ubiquitination (Figure 3E). The total surface content of mMHCII in these cells could comprise both endogenous and HA-tagged proteins. There

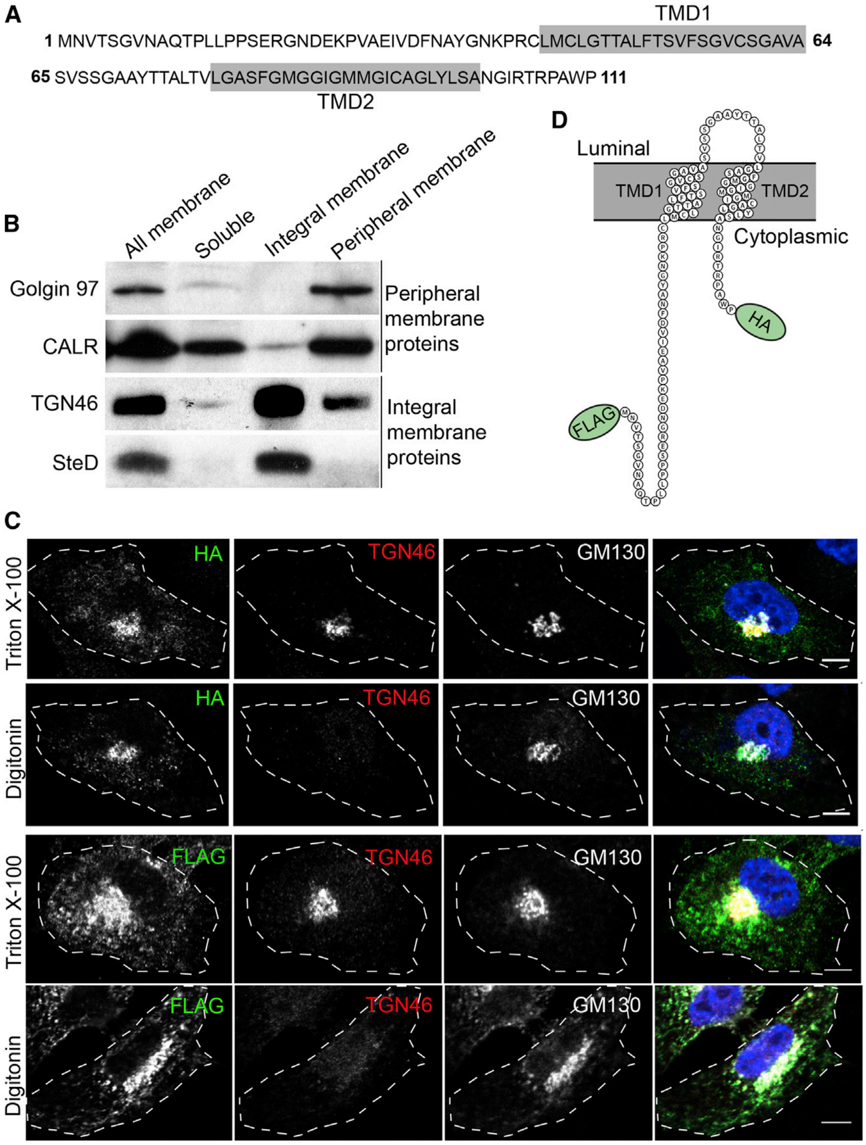

Figure 2. SteD Is an Integral Membrane Protein and Both the N- and C-termini Are Exposed to the Host Cell Cytosol

(A) Amino acid sequence of SteD showing transmembrane domains (TMD) predicted by TMHMM 2.0 software shaded in gray.

(B) Membrane fractionation of Mel Juso cells infected for 20 hr with Δ*steD psteD-2HA*. The soluble proteins were separated from total membrane proteins, which were later treated with 2.5 M urea to discriminate between the integral membrane and membrane-associated proteins by ultracentrifugation. Calreticulin (CALR) and Golgin 97 are membrane-associated proteins and TGN46 is an integral Golgi membrane protein.

(C) Mel Juso cells were infected with Δ*steD psteD-2HA* (top two panels) or transfected with vector encoding SteD fused at its N terminus to FLAG epitope (FLAG-SteD) (bottom two panels). The cells were semi- or completely permeabilized with digitonin or Triton X-100 to discriminate between cytoplasmic and Golgi luminal antigens, respectively. The antibodies recognizing the luminal portion of TGN46 or cytoplasmic GM130 were used as controls. The scale bar represents 5 μm.

(D) Schematic representation of SteD topology in the membrane (Protter 1.0 software; Omasits et al., 2014).

was no detectable difference in surface mMHCII (using mAb L243) between cells expressing endogenous proteins together with HA-DRβ or HA-DRβ K225R in the absence of SteD (Figure 3F), presumably reflecting a low turnover in these cells. SteD reduced surface mMHCII levels by approximately 90% in cells expressing HA-DRβ, but only by approximately 70% in cells expressing HA-DRβ K225R (Figures 3F and S5C). This shows that HA-DRβ K225R is at least partially resistant to SteD-dependent modification and suggests that the reduction of surface mMHCII in HA-DRβ K225R-expressing cells is due to the action of SteD on endogenous mMHCII.

SteD Interacts with MARCH8 and mMHCII

The amino acid sequence of SteD does not suggest an enzymatic activity or other function that would explain the ubiquitination of mMHCII. Therefore, we hypothesized that the enzyme causing SteD-dependent mMHCII ubiquitination was likely to be derived from the host cell. Two highly related members of the MARCH family of E3 integral membrane ubiquitin ligases (MARCH1 and MARCH8, which share 79.4% similarity; Fujita et al., 2013) reduce surface levels of mMHCII when overexpressed in Mel Juso cells (Lapaque et al., 2009b). MARCH1 is expressed in immature DCs and its expression is downregulated upon DC activation, leading to stabilization of mMHCII on the cell surface (De Gassart et al., 2008). In contrast, MARCH8 is expressed in a broad range of cell types, including Mel Juso cells (van de Kooij et al., 2013). However, Mel Juso cells do not express MARCH1 (Lapaque et al., 2009a). Therefore, we used small interfering (si)RNA to deplete MARCH8 and analyzed the ability of *Salmonella* to reduce surface levels of mMHCII in these cells (Figures 4A and S5D). Depletion of MARCH8, but not MARCH9 (Figure S2A), impaired the capacity of WT *Salmonella* to reduce surface levels of mMHCII (Figure 4A). In addition, infection of Mel Juso cells in which MARCH8 was depleted by small hairpin (sh)RNA (Figure S2B) revealed that SteD-dependent mMHCII ubiquitination requires MARCH8 (Figure 4B).

To investigate if SteD interacts with MARCH8, HEK293T cells (which do not express mMHCII) were transfected with plasmids encoding MARCH8-FLAG and GFP-SteD, then subjected to immunoprecipitation with GFP-Trap beads. MARCH8-FLAG co-immunoprecipitated with GFP-SteD, but not with GFP alone (Figure 4C). Immunofluorescence microscopy of transfected Mel Juso cells revealed that MARCH8-FLAG frequently co-localized with mMHCII in distinct vesicles (Figure 4D). These vesicles also

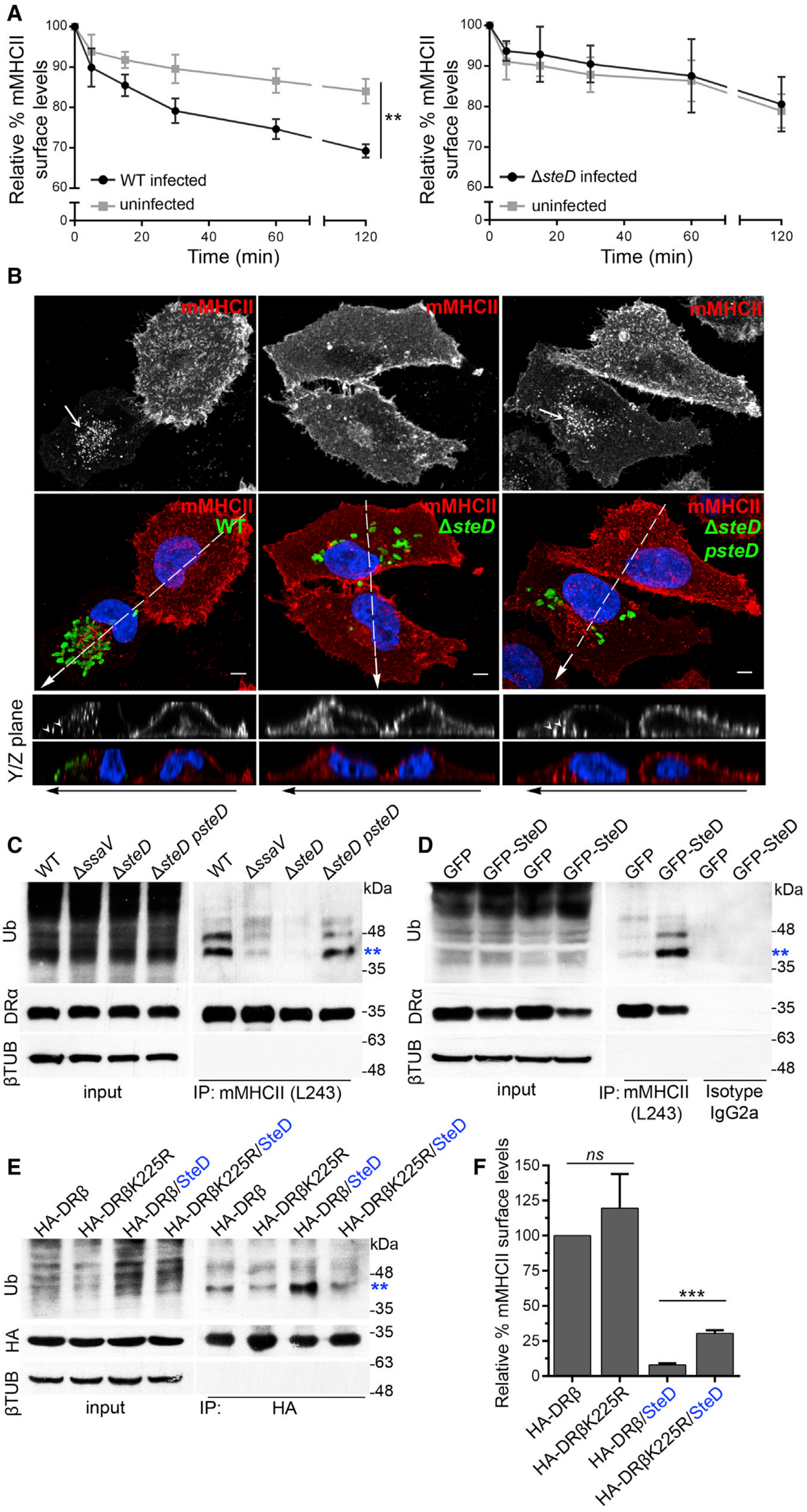

Figure 3. SteD Depletes Surface Levels of mMHCII and Increases Its Ubiquitination

(A) Flow cytometry analysis showing surface mMHCII in Mel Juso cells infected with WT-GFP or Δ*steD*-GFP strains compared to uninfected cells. The cells were infected for 16 hr and then labeled with mAb L243 on ice for 30 min to block internalization. The cells were exposed to 37°C to enable resumption of internalization. The surface levels of mMHCII at various time points were normalized to those at the time of transfer to 37°C (0 min), which represents 100%.

(B) Cells at 16 hr post invasion were labeled with mAb L243 on ice and incubated for another 4 hr in complete medium at 37°C. The cells were fixed and processed for immunofluorescence microscopy with DAPI nuclear stain (blue), anti-*Salmonella* CSA-1 (green), and anti-mouse secondary antibody against L243 (red). The images are maximum intensity Z projections showing the difference in mMHCII surface levels between cells infected with WT or Δ*steD psteD* and its intracellular accumulation (arrows). The scale bar represents 5 μm. The regions indicated by white dashed lines of Z projections are shown below in the YZ plane. The internalized mMHCII in cells infected with WT or Δ*steD psteD* strains are indicated by arrowheads.

(C) Mel Juso cells were infected at an MOI of 300:1 and at 16 hr post invasion lysed for immunoprecipitation with mAb L243 followed by western blot analysis with anti-ubiquitin (P4D1-HRP), anti-DRα, and anti-β tubulin antibodies.

(D) Stable Mel Juso cell lines expressing GFP or GFP-SteD were lysed and mAb L243 or IgG2a isotype control was used for immunoprecipitation. The immunoprecipitates were analyzed by western blot with the same antibodies as in (C).

(E) HA-tagged DRβ constructs (either wild-type [HA-DRβ] or with an arginine substitution of lysine 225 on DRβ cytoplasmic tail [HA-DRβ K225R]) were used to transduce Mel Juso cells or stable cells expressing GFP-SteD. The cells were lysed and immunoprecipitated with anti-HA antibody coupled to agarose beads followed by western blot analysis with anti-HA, anti-ubiquitin (P4D1-HRP), and anti-β tubulin antibodies. The blot shown is representative of three separate experiments in which band intensities, normalized to corresponding HA bands, are 1.00 ± 0.02 (DRβ), 0.59 ± 0.06 (DRβK225), 1.42 ± 0.44 (DRβ/SteD), and 0.50 ± 0.13 (DRβK225/SteD) (Student's t test $p < 0.03$, comparison between DRβ/SteD and DRβK225/SteD). Double asterisks in (C)–(E) denote di-ubiquitinated DRβ.

(F) Surface levels of mMHCII on stable cells described in (E), measured by mAb L243 labeling and flow cytometry. The data shown are surface levels of mMHCII relative to that in cells expressing HA-DRβ. ***$p < 0.001$, **$p < 0.01$, and not significant, *ns* (Student's t test). The data shown are surface levels of mMHCII relative to that in cells expressing HA-DRβ.

CellPress

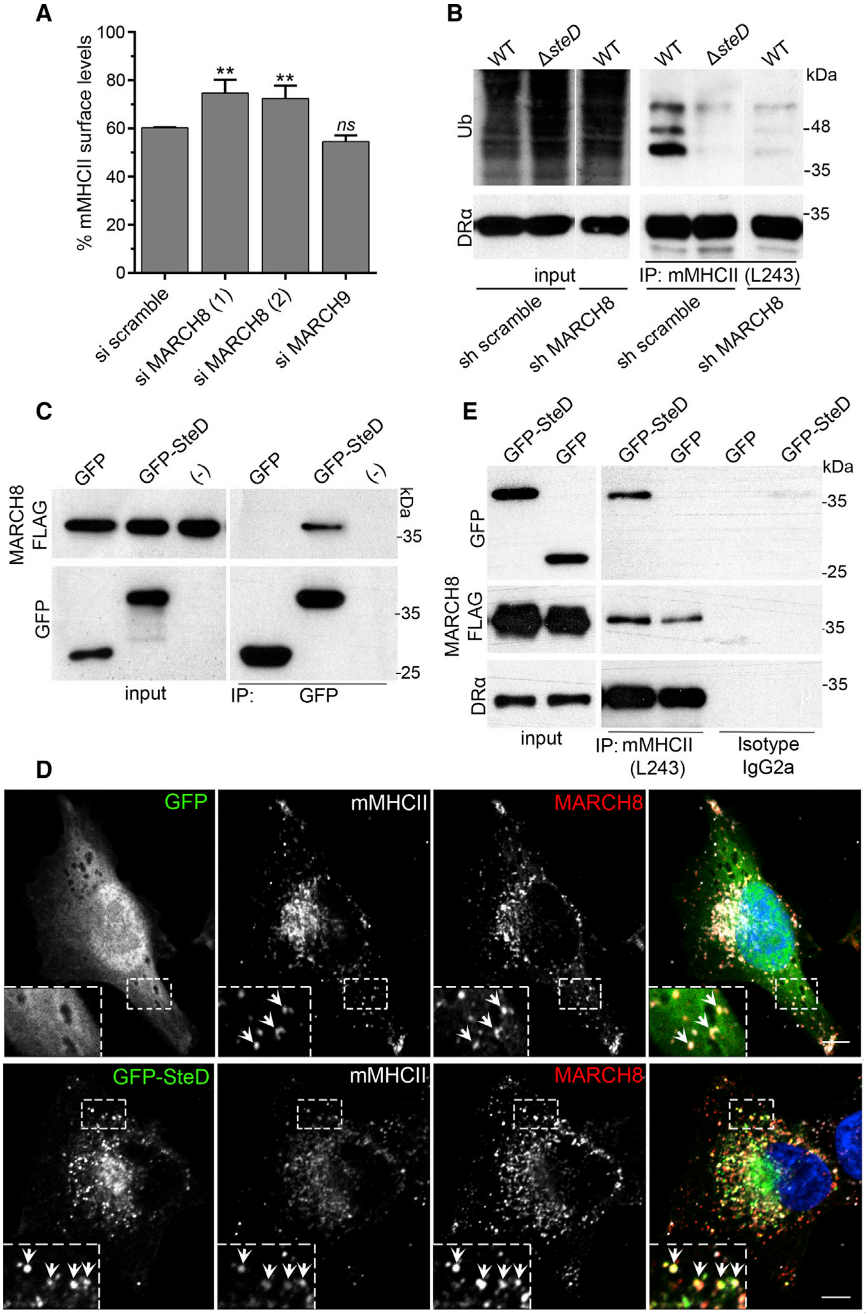

Figure 4. Interactions between SteD, MARCH8, and mMHCII

(A) Flow cytometry analysis of Mel Juso cells that were treated with siRNA prior to infection with WT-GFP *Salmonella*. The data represent surface levels of mMHCII in infected cells as a percentage of those in uninfected cells from the same sample and are the means ± SD from three independent experiments.

(B) Mel Juso cells transduced with shRNA to knockdown MARCH8 or a scramble control were infected at an MOI of 300:1 and at 16 hr post invasion lysed for immunoprecipitation with mAb L243 followed by western blot analysis with anti-ubiquitin (P4D1-HRP) and anti-DRα. sh MARCH8 and sh scramble images after immunoprecipitation (IP) are from the same blot and exposure.

(C) HEK293T cells transfected with vectors expressing MARCH8-FLAG and GFP-SteD or GFP alone were used for immunoprecipitation with GFP-Trap beads. (–) indicates MARCH8-FLAG alone. The immunoprecipitates were analyzed by western blot using anti-GFP and anti-FLAG antibodies.

(D) Stable Mel Juso cells expressing GFP-SteD and MARCH8-FLAG were analyzed by immunofluorescence with anti-FLAG (red) and mAb L243 against mMHCII (gray), the arrows indicate vesicles in which the three proteins co-localized. The scale bar represents 5 μm.

(E) Stable Mel Juso cell lines expressing both MARCH8-FLAG and GFP-SteD or MARCH8-FLAG and GFP alone were used for immunoprecipitation with mAb L243 (for mMHCII) or isotype control. The immunoprecipitates were analyzed by western blot using anti-GFP, anti-FLAG, and anti-DRα antibodies. The samples in (A) were compared to siRNA scramble by one-way ANOVA followed by Dunnett's multiple comparison test. **p < 0.01 and not significant, *ns*.

contained GFP-SteD, but not the GFP control (Figure 4D). To investigate physical interactions between SteD, MARCH8, and mMHCII in these cells, mMHCII was immunoprecipitated with mAb L243. mMHCII interacted with GFP-SteD, but not with GFP (Figure 4E). It also interacted with MARCH8-FLAG in the presence or absence of SteD (Figure 4E). Live cell imaging analysis of Mel Juso cells expressing mCherry-MARCH8 and GFP-SteD revealed that both proteins were often present in the same vesicles, moving in both anterograde and retrograde directions (Movie S2; Figures S2C and S2D).

To identify regions of SteD that are important for interacting with mMHCII and MARCH8, we first constructed truncated versions lacking either the N-terminal or C-terminal cytoplasmic domains. The amount of the N-terminal SteD mutant fused to GFP was much lower than that of the wild-type fusion protein, preventing analysis of its properties. However, a version lacking the C-terminal 11 amino acids (GFP-SteD^{Ct}) was expressed normally. GFP-SteD^{Ct} also integrated into host cell membranes (Figure S3A), interacted with MARCH8-FLAG (Figure 5A), but had reduced binding to mMHCII (Figure 5B). In infected cells, SteD^{Ct} induced weak ubiquitination of mMHCII (Figure 5C), and after transfection it caused significantly less reduction of mMHCII surface levels (Figure 5D). A more detailed mutational analysis was carried out by making a series of 20 small alanine substitutions (SteD^{1}–SteD^{20}) in blocks of five or six amino acids, to cover the entire sequence of SteD (Figure 5E). These variants were tagged with GFP and analyzed after transfection to circumvent any defects in their translocation from bacteria. There were two mutants (SteD^{9} and SteD^{13}) that were unstable and did not localize at the Golgi network (Figures S4A and

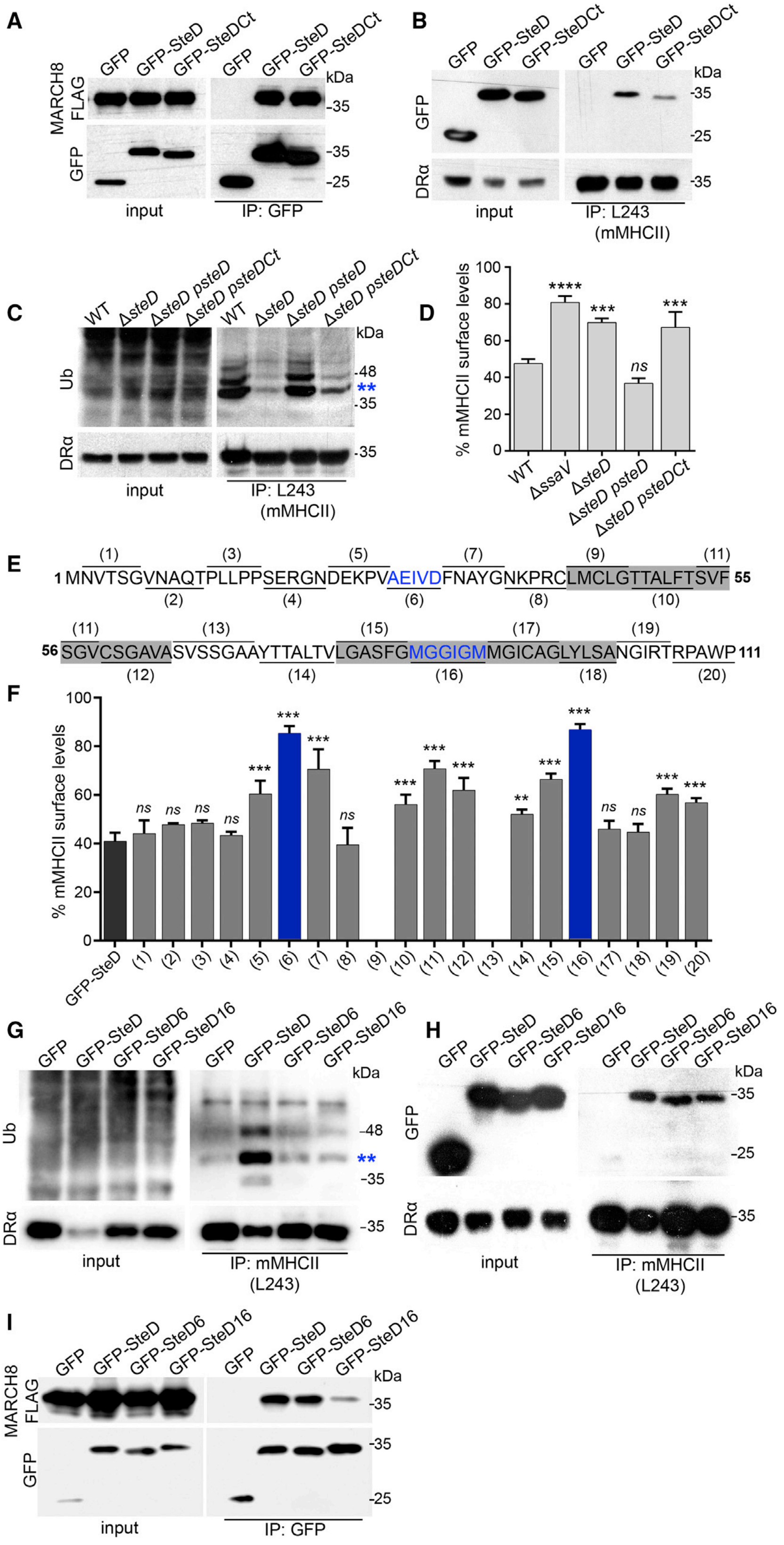

Figure 5. Mutational Analysis of SteD

(A) HEK293T cells transfected with vectors expressing MARCH8-FLAG and GFP, GFP-SteD, or GFP-SteD^{Ct} were used for immunoprecipitation with GFP-Trap beads and analyzed by western blot with anti-GFP and anti-FLAG antibodies.

(B) Stable Mel Juso cell lines expressing GFP, GFP-SteD, and GFP-SteD^{Ct} were used for immunoprecipitation with mAb L243 (for mMHCII). The immunoprecipitates were analyzed by western blot with anti-GFP and anti-DRα antibodies. The blot shown is representative of three separate experiments in which the mean intensity of GFP-SteD^{Ct} compared to GFP-SteD after immunoprecipitation was 0.21 ± 0.14 ($p < 0.02$).

(C) Mel Juso cells were infected at an MOI of 300:1 and at 16 hr post invasion lysed for immunoprecipitation with mAb L243 followed by western blot analysis with anti-ubiquitin (P4D1-HRP) and anti-DRα antibodies.

(D) Mel Juso cells were transfected with vectors encoding GFP, GFP-SteD, and GFP-SteD^{Ct}, and mMHCII surface levels were analyzed by flow cytometry. The data represent surface levels of mMHCII in transfected cells as a percentage of those in untransfected cells from the same sample and are the means ± SD from three independent experiments.

(E) Schematic representation of SteD amino acids substituted with alanine. The transmembrane regions are shaded in gray.

(F) Mel Juso cells were transfected with vectors encoding mutated versions of SteD fused to GFP, and mMHCII surface levels were analyzed by flow cytometry. The data were analyzed as for (D) above.

(G) Stable Mel Juso cell lines expressing GFP, GFP-SteD, GFP-SteD^{6}, or GFP-SteD^{16} were lysed and mAb L243 was used for immunoprecipitation and analysis as in (C).

(H) The stable cells used in (G) were used for immunoprecipitation with mAb L243 (for mMHCII) and analyzed as in (B).

(I) HEK293T cells transfected with vectors expressing MARCH8-FLAG and GFP, GFP-SteD, GFP-SteD^{6}, or GFP-SteD^{16} were used for immunoprecipitation with GFP-Trap beads and analyzed as in (A). The blot shown is representative of three separate experiments in which the mean intensity of GFP-SteD^{16} compared to GFP-SteD after immunoprecipitation was 0.12 ± 0.07 ($p < 0.02$). The data in (D) and (F) were compared to GFP-SteD by one-way ANOVA followed by Dunnett's multiple comparison test. ****$p < 0.0001$, ***$p < 0.001$, **$p < 0.01$, and not significant, *ns*.

S4B). Expression and localization of the remaining 18 GFP-SteD mutants were similar to wild-type SteD (Figures S4A and S4B). Measurement of mMHCII surface levels in cells producing these mutants revealed several regions that are important for SteD function (Figure 5F). The two mutants having the weakest effect on mMHCII surface levels were SteD^{6} and SteD^{16}; in both cases this was accompanied by reduced ubiquitination of mMHCII (Figure 5G). SteD^{6} and SteD^{16} integrated into host cell membranes (Figures S3B and S3C) and were not impaired in their ability to interact with mMHCII (Figure 5H). By contrast, in HEK293T cells, SteD^{16}, but not SteD^{6}, failed to interact efficiently with MARCH8-FLAG (Figure 5I). Together, the mutational analysis of SteD shows that the C-terminal 11 amino acids and second transmembrane domain are important for interactions with mMHCII and MARCH8, respectively. Furthermore, although SteD^{6} interacted with both mMHCII and MARCH8, it failed to induce ubiquitination and removal of surface mMHCII, which suggests that SteD has additional function(s) in regulating MARCH8-dependent ubiquitination of mMHCII.

SteD-Mediated Reduction of Surface mMHCII Affects T Cell Proliferation

Efficient MHCII-dependent T cell activation requires recognition of both peptide-loaded mMHCII and the co-stimulatory molecule CD86/B7.2, whose surface level is also regulated by MARCH8/1 ubiquitination (Bartee et al., 2004; Goto et al., 2003). We confirmed that WT *Salmonella* reduced overall mouse MHCII (I-A/I-E haplotypes) surface levels in bone marrow-derived DCs (BMDCs) and showed that the reduction did not occur in BMDCs infected with Δ*ssaV* or Δ*steD* mutants (Figure 6A). BMDCs infected with WT *Salmonella* also displayed reduced surface levels of B7.2, and this effect was lost following infection with the Δ*ssaV* or Δ*steD* mutants (Figure 6B). To assess the effect of SteD on T cell proliferation, infected BMDCs were incubated with Ovalbumin (OVA)-peptide at 16 hr post uptake, co-cultured for 3 days with T cells expressing a T cell receptor specific for OVA, and labeled with carboxyfluorescein diacetate succinimidyl ester (CFSE) to determine T cell proliferation by flow cytometry. WT *Salmonella* induced a strong SteD-dependent inhibition of T cell proliferation (Figures 6C and 6D), consistent with its effect on both mMHCII and CD86/B7.2. To determine if SteD influences MHCII surface levels during infection of mice, DCs were isolated at 48 hr post oral inoculation from mesenteric lymph nodes. Quantification of total surface MHCII (I-A/I-E) by flow cytometry revealed that DCs infected by the WT-GFP strain had significantly reduced levels of MHCII compared to DCs infected with the Δ*steD*-GFP strain (Figures 6E and S5E). Finally, to assess the effect of SteD on T cell activation in vivo, mice were infected for 17 days with WT or *steD* mutant *Salmonella*, then spleen cells were recovered and T cells analyzed for activation markers by flow cytometry. Analysis of CD4^{+} T cells from mice with similar bacterial loads revealed that there were significantly more activated T cells in spleens carrying the *steD* mutant compared to spleens from mice infected with WT bacteria (Figures 6F and S5F).

DISCUSSION

Numerous bacterial effectors have been characterized that interfere with distinct aspects of the mammalian innate immune system (McGuire and Arthur, 2015), but much less is known about effectors that inhibit the development of adaptive immunity. Several groups have shown that *Salmonella* inhibits surface presentation of MHCII (Cheminay et al., 2005; Halici et al., 2008; Jackson et al., 2013; Lapaque et al., 2009a; Mitchell et al., 2004). This was attributed to the SPI-2 T3SS (Cheminay et al., 2005; Mitchell et al., 2004) and the actions of several effectors that interfere with vesicular trafficking (Cheminay et al., 2005; Halici et al., 2008). Lapaque et al. (2009a) showed that *Salmonella* specifically reduces the surface levels of mMHCII through its ubiquitination. Several possible mechanisms were proposed to account for this, including delivery of a *Salmonella* ubiquitinating enzyme and the relocalization of mMHCII or host ubiquitin enzymes to facilitate their interactions (Lapaque et al., 2009a). The results presented here, showing that SteD interacts directly or indirectly with both MARCH8 and mMHCII, are consistent with the latter proposal. These interactions might occur in a binary manner, with some SteD molecules interacting with mMHCII and others with MARCH8. However, mutational analysis revealed two distinct regions of SteD that are important for suppressing surface levels of mMHCII: the cytoplasmic C-terminal 11 amino acids are involved in binding mMHCII and part of the second transmembrane domain is important for binding to MARCH8. Both regions are also required for ubiquitination and reduced surface load of mMHCII. This suggests that SteD functions as an adaptor, facilitating interactions between enzyme and substrate. The finding that a transmembrane region of SteD is required for efficient binding to MARCH8 is consistent with other work showing transmembrane domain-dependent recognition of proteins by MARCH1 and MARCH8 (Fujita et al., 2013; Goto et al., 2003) and, more generally, the existence of several integral membrane ubiquitin ligase adaptors that regulate E3 ligase/substrate interactions (Léon and Haguenauer-Tsapis, 2009).

Two results indicate that SteD has additional function(s). First, in Mel Juso cells, similar amounts of MARCH8 interacted with mMHCII in the presence or absence of SteD. Second, the SteD^{6} mutant was defective in ubiquitinating and reducing surface levels of mMHCII, yet retained binding to MARCH8 and mMHCII. This indicates that SteD might also activate MARCH8, which is interesting in light of other evidence suggesting the existence of an endogenous cofactor for MARCH8 (Fujita et al., 2013). Thus, SteD might mimic the activity of an as yet unidentified mammalian membrane adaptor/cofactor that functions with MARCH1/8 to provide substrate specificity and regulate E3 ligase activity.

The finding that the majority of SteD locates to the TGN raises interesting questions regarding its transport from the SCV, Golgi membrane insertion, and the significance of Golgi localization. A substantial amount of MARCH8 co-localized with non-Golgi associated SteD, and vesicles containing both proteins were observed moving throughout the host cell (Figures S2C and S2D). Since mMHCII does not traffic to the Golgi network (Neefjes et al., 2011), the simplest hypothesis is that SteD interacts with MARCH8 in Golgi membranes or Golgi-derived vesicles, and the proteins are then transported to endosomes and possibly to the plasma membrane to interact with mMHCII. Cho et al. (2015) showed that in immature mouse DCs, MARCH1 does not affect the rate of mMHCII endocytosis, but instead

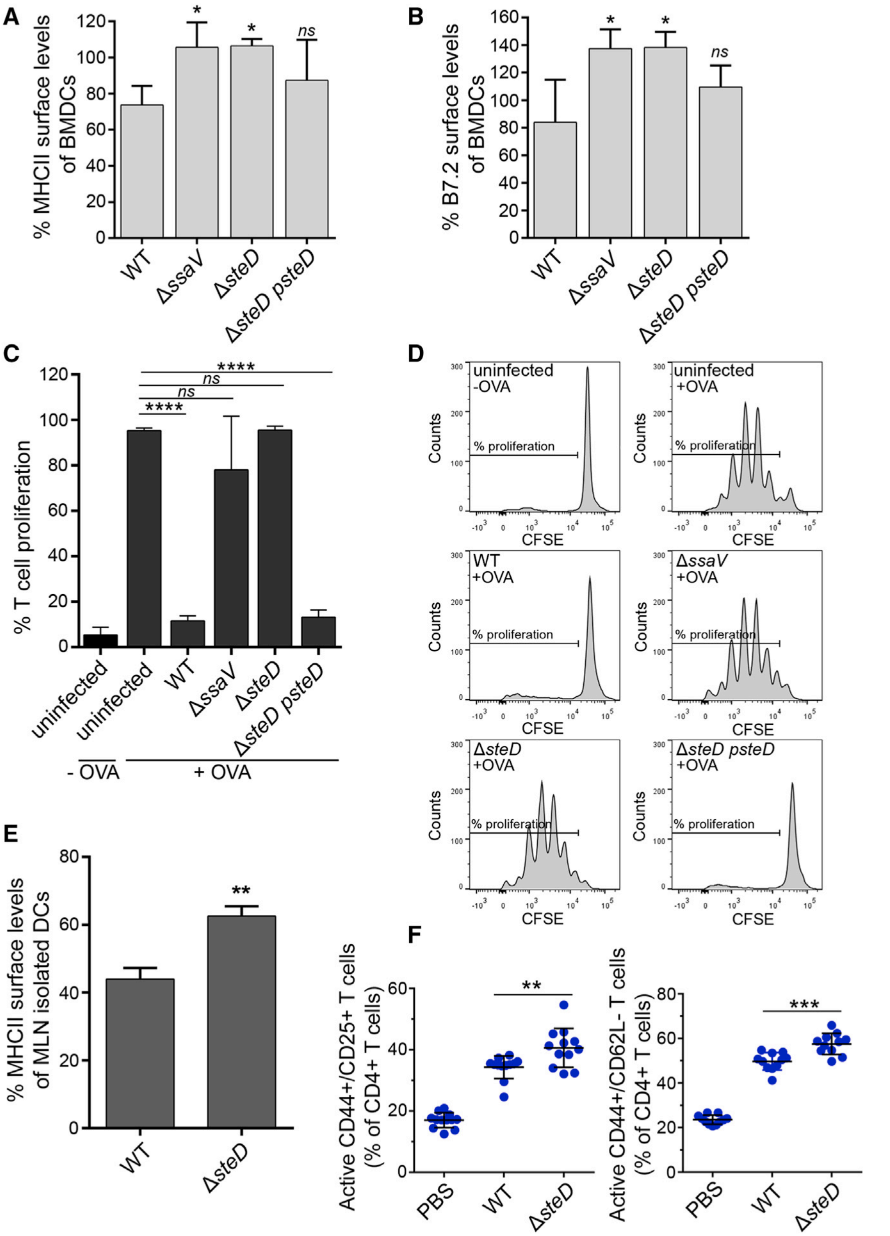

Figure 6. SteD Suppresses T Cell Proliferation

(A) Mouse BMDCs were infected with the indicated bacterial strains and total MHCII surface levels (I-A/I-E haplotypes) were quantified by flow cytometry at 20 hr post uptake.

(B) BMDCs were infected as in (A), and B7.2 surface levels were quantified by flow cytometry at 20 hr post uptake.

(A and B) Surface levels of proteins in infected cells are represented as a percentage of those in uninfected cells from the same sample and are the means ± SD from four independent experiments.

(C) Infected BMDCs were incubated with OVA peptide and co-cultured with T cells labeled with CFSE for 3 days. T cell proliferation was analyzed by flow cytometry after labeling cells with anti-CD3, anti-V alpha2, and anti-CD4 antibodies. The uninfected BMDCs incubated or not with OVA-peptide were used as controls. The results shown represent the % of T cells that proliferated and are the means ± SD from quadruplicate samples in three independent experiments.

(D) Representative FACS histograms showing T cell proliferation measured by CFSE levels in different conditions.

(E) Dendritic cells from mesenteric lymph nodes of mice infected with WT-GFP or Δ*steD*-GFP *Salmonella* by oral gavage were isolated at 48 hr post inoculation and total MHCII surface levels were measured by flow cytometry. The data represent surface levels of MHCII in infected BMDCs as a percentage of those in uninfected cells from the same sample and are the means ± SD from three independent experiments.

(F) Activated T cells (CD25+CD44+ and CD62L− CD44+) as a percentage of total $CD4^+$ T cells isolated from the spleens of mice infected by *Salmonella* WT or Δ*steD* strains at day 17 post inoculation. The data in (A) and (B) were analyzed by comparison with WT and in (C) by comparison with uninfected +OVA by one-way ANOVA followed by Dunnett's multiple comparison test. The data in (E) were analyzed by Student's t test. The data in (F) were analyzed by two-tailed pairwise t test using pairs shown in Table S2. ****$p < 0.0001$, **$p < 0.01$, *$p < 0.02$, and not significant, *ns*.

prevents its recycling to the plasma membrane from intracellular vesicles. Ubiquitination of mMHCII by MARCH1 in early endosomes promotes its sorting into multivesicular bodies and lysosomal degradation (Cho et al., 2015; Furuta et al., 2013). The presence of SteD and MARCH8 on intracellular vesicles suggests that its effect on mMHCII is to prevent recycling to the plasma membrane, but further work is needed to establish if SteD also acts on the plasma membrane pool of mMHCII.

Whereas the effect of SteD on DC surface MHCII (Figure 6A) appears relatively modest, the inhibition of T cell proliferation was very dramatic (Figure 6C). However, the antibody used to detect surface MHCII on DCs does not discriminate between mMHCII and Ii-MHCII forms and therefore is likely to underestimate the effect of SteD on mMHCII in this assay. Furthermore, we found that SteD also reduces surface levels of the co-stimulatory molecule CD86/B7.2, which is also regulated by MARCH1 (Bartee et al., 2004) and required for full T cell activation. Whether this results from a similar mechanism to that of SteD on mMHCII remains to be determined, but the combined effect of SteD on both mMHCII and CD86/B7.2 provides an explanation for its potent effect on T cell activation (Figure 6C).

MARCH1 expression was reported to be restricted to secondary lymphoid tissues, while MARCH8 is ubiquitously expressed (Bartee et al., 2004). In immature DCs, the expression of MARCH1 is relatively high, enabling it to prevent mMHCII from recycling to the cell surface, but its expression is reduced by over 90% following DC activation (Cho et al., 2015; De Gassart et al., 2008). Conversely, MARCH8 expression remains unchanged during the maturation process (De Gassart et al., 2008) and functions in transferrin receptor recycling (Fujita

et al., 2013), but does not seem to contribute to recycling of mMHCII. It is possible that SteD diverts MARCH8 from its normal function or activity to increase ubiquitination of mMHCII in mature DCs after *Salmonella* infection. However, we did not detect a difference in overall surface levels of transferrin receptor in DCs infected with WT or *steD* mutant bacteria (Figure S5G), so the identity of the MARCH ligase targeted by SteD in DCs remains to be established.

SteD accounted for potent inhibition of T cell proliferation by *Salmonella* in vitro. In vivo, DCs from mouse mesenteric lymph nodes infected with the WT bacteria displayed less cell surface MHCII than DCs infected with the *steD* mutant. Furthermore, we detected a significant SteD-dependent suppression of T cell activation in spleens of mice infected with *Salmonella.* Therefore, the in vitro effects of SteD on mMHCII and T cells are likely to be physiologically important. Several bacterial pathogens, including *Salmonella*, encode ubiquitin ligases that target host cell proteins (Ashida et al., 2014). We show here the existence of a distinct mechanism, by which a bacterial effector binds and possibly activates a host E3 ligase to force the inappropriate ubiquitination of a substrate. SteD appears to be the first example of a bacterial protein that targets mMHCII to suppress adaptive immune responses. In view of the critical role of MHCII molecules in the development of adaptive immunity to many bacteria, it is possible that other pathogens might use similar mechanisms to promote their virulence.

EXPERIMENTAL PROCEDURES

Bacterial Strains, Plasmids, and Antibodies

All bacterial strains, plasmids, and antibodies used in this study are described in Table S1. Isogenic mutants of *S. enterica* serovar Typhimurium were constructed using one-step chromosomal inactivation system (Datsenko and Wanner, 2000). Wild-type and mutant strains were transformed with plasmids pFPV25.1 and pFCcGi for GFP and mCherry expression, respectively, when required.

Cell Culture and Infection

Mel Juso and HEK293T cells were maintained in DMEM containing 10% fetal calf serum (FCS) at 37°C in 5% CO_2. L243-producing hybridoma cells were maintained in DMEM containing 10% FCS and reduced to 5% FCS when cell density was high to harvest supernatant containing antibodies. Primary BMDCs were extracted from C57BL/6 mice (Charles River). Cells recovered from tibias and femurs were grown at 37°C in 5% CO_2 in RPMI-1640 supplemented with 10% FCS, 2 mM glutamine, 1 mM sodium pyruvate, 10 mM HEPES, 0.05 M β-mercaptoethanol, and 20 ng/mL granulocyte-macrophage colony-stimulating factor (GM-CSF) (PeproTech). After 3 days of culture, fresh complete medium was added to the growing cells. On day 6, medium was replaced by fresh complete medium, and cells were harvested on day 8 and seeded 6 hr prior to infection. Bacteria were grown in Luria Bertani (LB) medium with shaking at 37°C, supplemented with carbenicillin or kanamycin as required (50 μg/mL). Mel Juso cells were infected for 30 min at MOI of 100:1 with late log-phase *Salmonella*. BMDCs were infected for 30 min at MOI 10:1 with stationary-phase *Salmonella* opsonized in 20% mouse serum. Cells were washed with PBS twice and incubated in fresh medium containing gentamicin (100 μg/mL) for 1 hr to kill extracellular bacteria. After 1–2 hr, the antibiotic concentration was reduced to 20 μg/mL, and the cells were processed 16–20 hr post uptake. Approximately 70% of Mel Juso cells and BMDCs became infected using these conditions.

siRNA and DNA Transfection and Retroviral Transduction

Details of siRNA and shRNA transfection are in the Supplemental Information. Knock down of MARCH8 and MARCH9 mRNA was assessed by qPCR using SYBR GREEN and conditions described previously (Lapaque et al., 2009a). DNA transfection procedures were carried out using Lipofectamine 2000 according to the manufacturer's protocol (Life Technologies). Samples were prepared for analysis 20 hr after transfection. For retrovirus production, HEK293T cells were transfected with proviral plasmid M5P together with helper plasmids using Lipofectamine 2000 as described previously (Randow and Sale, 2006). Mel Juso cells were transduced with virus in culture supernatant containing 8 μg/mL polybrene (Sigma-Aldrich) by centrifugation at 650 *g* for 1 hr at 37°C. Transduced cells were selected in 2 μg/mL puromycin or GFP-positive cells were sorted by fluorescence-activated cell sorting (FACS) as required.

Flow Cytometry

To measure surface levels of mMHCII, Mel Juso cells that had been either transfected or infected with *Salmonella* were collected using Cell Dissociation Buffer (Sigma-Aldrich) and incubated first with mAb L243 and then anti-mouse secondary antibody diluted in FACS buffer (5% FCS and 2 mM EDTA in PBS) at 4°C for 30 min. After washings with cold PBS, cells were fixed in 3% paraformaldehyde and analyzed using a FACS Calibur or Fortessa flow cytometer (BD Biosciences) and FlowJo v10 software. mMHCII surface levels were calculated as geometric mean fluorescence of infected cells (GFP-positive)/geometric mean fluorescence of uninfected cells (GFP-negative) × 100. For complementation analysis of bacterial mutants, Mel Juso cells were infected with non-fluorescent bacterial strains and, after labeling of surface mMHCII, cells were fixed and incubated with anti-*Salmonella* CSA-1 antibody diluted in 10% FCS and 0.1% saponin in PBS for 1 hr at room temperature, followed by anti-goat secondary antibody labeling under the same conditions. Surface levels of MHCII or B7.2 in BMDCs were calculated as median fluorescence of infected cells (CSA1-positive)/median fluorescence of uninfected cells (CSA1-negative) × 100. For the internalization assay, Mel Juso cells were infected with WT-GFP or Δ*steD*-GFP *Salmonella* strains and harvested 16 hr post invasion. The surface mMHCII molecules were labeled with mAb L243 for 30 min at 4°C. Cells were washed in cold medium, resuspended, and split into 1.5 mL tubes containing pre-warmed medium in a water bath at 37°C. Aliquots were removed at various time points, diluted in ice-cold FACS buffer, and kept on ice. At the last time point, cells were centrifuged at 4°C and resuspended in FACS buffer containing Alexa 647-conjugated goat anti-mouse antibody. After 30 min at 4°C, cells were washed, fixed in 3% paraformaldehyde, washed, and analyzed by flow cytometry to quantify mMHCII/L243 complexes remaining at the cell surface. GFP-positive cells were considered infected and GFP-negative uninfected. mMHCII surface levels were normalized to those detected at the beginning of the experiment (100%).

Immunofluorescence Microscopy

Cells were fixed and immunolabeled with antibodies listed in Table S1. Samples were mounted and analyzed using a confocal laser-scanning microscope LSM 710 (Zeiss). Co-localization analysis was done with Zeiss Zen 710 software, with thresholds set using individual controls lacking each analyzed primary antibody. Further details are in Supplemental Information.

Membrane Fractionation

Approximately 5 × 10^7 Mel Juso cells were infected as described above. At 20 hr post invasion, cells were collected and lysed by mechanical disruption using a Dounce homogenizer in 600 μL of homogenization buffer containing 250 mM sucrose, 3 mM imidazole (pH 7.4), and 1 mM PMSF. Cell extracts were centrifuged at 1,800 *g* for 15 min and the post nuclear supernatant was centrifuged at 100,000 *g* for 1 hr at 4°C, giving rise to a pellet containing total membrane proteins and supernatant containing soluble proteins. The pellet was resuspended in 600 μL of 2.5 M urea and incubated for 15 min on ice. This suspension was centrifuged again at 100,000 *g* for 1 hr at 4°C, resulting in a second pellet containing integral membrane proteins and a supernatant containing membrane-associated proteins. The volume of all fractions was made to 600 μL with homogenization buffer, and proteins were analyzed by western blot.

Immunoprecipitation and Western Blot

Analysis of mMHCII ubiquitination was done as described previously with minor modifications (Lapaque et al., 2009a). Further details are in Supplemental Information.

Live Imaging

For live cell imaging, Mel Juso cells stably expressing GFP-SteD were seeded at a density of approximately 5×10^4 cells per dish onto 35 mm glass-bottom culture dishes (MatTek). Cells were transfected for 20 hr with a vector expressing mCherry-MARCH8 using Lipofectamine 2000 (Life Technologies) following manufacturer's instructions. Before imaging, cells were washed and incubated in imaging medium (Opti-MEM containing 10% FCS and 25 mM HEPES pH 7.2). Culture dishes were sealed with vaseline and analyzed using a LSM 710 microscope (Zeiss) at 37°C. Samples were imaged with a 63 × oil objective.

T Cell Proliferation Assay

BMDCs were prepared from C57BL/6 mice as described above. The CD11c-positive cell population was enriched using MAC sorting (Miltenyi Biotec) to a purity of 95%. Cells were infected in 15 mL tubes at an MOI 10:1 for 30 min in RPMI 10% FCS, washed, and treated with gentamicin as described above. After 16 hr of infection, cells were harvested with cold PBS and incubated with OVA peptide (ISQAVHAAHAEINEAGR) at 5 μM in RPMI containing 10% FCS for 1 hr. Cells were washed, counted, and incubated with T cells in 96-well plates. T cells expressing OVA-specific T cell receptor (TCR) were isolated from cell suspensions of spleens and lymph nodes of OT-II mice by magnetic sorting of $CD4^+$ cells (Miltenyi Biotec) and labeled with CFSE as described previously (Quah and Parish, 2010). CD4 T cells were incubated with DCs at a ratio of 3:1 in a final volume of 200 μL of medium containing 20 μg/mL gentamicin. At 3 days later, cells were centrifuged and resuspended in 150 μL of FACS buffer containing anti-CD3, anti-V alpha2, and anti-CD4 antibody and incubated for 30 min on ice. Cells were washed and resuspended in 150 μL of FACS buffer containing 6 μm blank calibration particles (BD Biosciences) as an internal control for normalization of T cell numbers.

Mouse Infection and Isolation of DCs

Female C57BL/6 mice, 6–8 weeks old, were infected by oral gavage with 1×10^{10} colony forming units (CFU) of late log-phase GFP-*Salmonella* in 200 μL of PBS containing 3% $NaHCO_3$. Mesenteric lymph nodes were isolated 48 hr after inoculation and cells were collected through a 70 μm cell strainer following tissue disruption. DCs were purified from single-cell suspensions using anti-CD11c antibody-coupled magnetic beads (Miltenyi Biotec) according to the manufacturer's instructions. Purified DCs were labeled on ice with anti-I-A/I-E antibody (recognizing mouse MHC class II molecules), diluted in FACS buffer for 20 min on ice. Purity was assessed by anti-CD11c antibody labeling. Discrimination between infected and uninfected cells was based on GFP fluorescence and MHCII geometric mean fluorescence calculated as described above. For analysis of T cell activation, mice were inoculated intraperitoneally with 5×10^5 CFU of virulence-attenuated *S*. Typhimurium strain SL3261 or SL3261 *ΔsteD* that had been grown in LB broth to late exponential phase. Spleens were harvested 17 days later and homogenized in Hanks balanced salt solution (HBSS) supplemented with 10 mM HEPES and 2% FCS. A portion of spleen homogenate was plated to enumerate bacterial CFU and spleens with similar bacterial loads (Table S2) were analyzed further. Erythrocytes were lysed using ACK buffer (150 mM NH_4Cl, 10 mM $KHCO_3$, and 0.1 mM EDTA). After blocking surface Fc receptors using FcR Blocking Reagent (Miltenyi Biotec), the remaining splenocytes were labeled using anti-CD3ε, anti-CD4, anti-CD25, anti-CD44, and anti-CD62L antibodies. Activation of gated $CD4^+CD3\varepsilon^+$ cells was determined on the basis of surface-localized CD25, CD62L, and CD44.

Ethics Statement

Mice experiments were conducted in accordance to European Directive 2010/63/EU regulations with approval from Imperial College, London Animal Welfare and Ethical Review Body (ICL AWERB) under the Personal Project license of David Holden.

SUPPLEMENTAL INFORMATION

Supplemental Information includes Supplemental Experimental Procedures, five figures, two tables, and two movies and can be found with this article online at http://dx.doi.org/10.1016/j.chom.2016.10.007.

AUTHOR CONTRIBUTIONS

E.B.-S., C.H.D., A.K., E.A., O.C., L.A.R., E.J., M.L., and A.S.R. performed research; E.B.-S., C.H.D., A.K., E.A., O.C., and D.W.H. analyzed data; N.L. and S.H.E.K. contributed with discussions; and E.B.-S. and D.W.H. wrote the paper.

ACKNOWLEDGMENTS

We thank members of the Holden laboratory and Dr. Sophie Helaine for helpful suggestions. We thank Felix Randow and Teresa Thurston for plasmids. We are grateful to Stéphane Méresse for providing a L243 hybridoma cell line and Jacques Neefjes for providing Mel Juso cells. This work was supported by grants from the Medical Research Council (MR/K027077/1) and Wellcome Trust (095484/Z/11/Z) to D.W.H. and a CAPES Fellowship (#9236/13-9) awarded to E.B.-S.

Received: July 5, 2016
Revised: September 9, 2016
Accepted: October 11, 2016
Published: November 9, 2016

REFERENCES

Ashida, H., Kim, M., and Sasakawa, C. (2014). Exploitation of the host ubiquitin system by human bacterial pathogens. Nat. Rev. Microbiol. *12*, 399–413.

Bartee, E., Mansouri, M., Hovey Nerenberg, B.T., Gouveia, K., and Früh, K. (2004). Downregulation of major histocompatibility complex class I by human ubiquitin ligases related to viral immune evasion proteins. J. Virol. *78*, 1109–1120.

Bijlmakers, M.J., Benaroch, P., and Ploegh, H.L. (1994). Assembly of HLA DR1 molecules translated *in vitro*: binding of peptide in the endoplasmic reticulum precludes association with invariant chain. EMBO J. *13*, 2699–2707.

Cheminay, C., Möhlenbrink, A., and Hensel, M. (2005). Intracellular *Salmonella* inhibit antigen presentation by dendritic cells. J. Immunol. *174*, 2892–2899.

Cho, K.J., Walseng, E., Ishido, S., and Roche, P.A. (2015). Ubiquitination by March-I prevents MHC class II recycling and promotes MHC class II turnover in antigen-presenting cells. Proc. Natl. Acad. Sci. USA *112*, 10449–10454.

Datsenko, K.A., and Wanner, B.L. (2000). One-step inactivation of chromosomal genes in *Escherichia coli* K-12 using PCR products. Proc. Natl. Acad. Sci. USA *97*, 6640–6645.

De Gassart, A., Camosseto, V., Thibodeau, J., Ceppi, M., Catalan, N., Pierre, P., and Gatti, E. (2008). MHC class II stabilization at the surface of human dendritic cells is the result of maturation-dependent MARCH I down-regulation. Proc. Natl. Acad. Sci. USA *105*, 3491–3496.

Fujita, H., Iwabu, Y., Tokunaga, K., and Tanaka, Y. (2013). Membrane-associated RING-CH (MARCH) 8 mediates the ubiquitination and lysosomal degradation of the transferrin receptor. J. Cell Sci. *126*, 2798–2809.

Furuta, K., Walseng, E., and Roche, P.A. (2013). Internalizing MHC class II-peptide complexes are ubiquitinated in early endosomes and targeted for lysosomal degradation. Proc. Natl. Acad. Sci. USA *110*, 20188–20193.

Goto, E., Ishido, S., Sato, Y., Ohgimoto, S., Ohgimoto, K., Nagano-Fujii, M., and Hotta, H. (2003). c-MIR, a human E3 ubiquitin ligase, is a functional homolog of herpesvirus proteins MIR1 and MIR2 and has similar activity. J. Biol. Chem. *278*, 14657–14668.

Halici, S., Zenk, S.F., Jantsch, J., and Hensel, M. (2008). Functional analysis of the *Salmonella* pathogenicity island 2-mediated inhibition of antigen presentation in dendritic cells. Infect. Immun. *76*, 4924–4933.

Jackson, N.P., Kang, Y.H., Lapaque, N., Janssen, H., Trowsdale, J., and Kelly, A.P. (2013). *Salmonella* polarises peptide-MHC-II presentation towards an unconventional Type B CD4+ T-cell response. Eur. J. Immunol. *43*, 897–906.

Jantsch, J., Cheminay, C., Chakravortty, D., Lindig, T., Hein, J., and Hensel, M. (2003). Intracellular activities of *Salmonella enterica* in murine dendritic cells. Cell. Microbiol. *5*, 933–945.

Krogh, A., Larsson, B., von Heijne, G., and Sonnhammer, E.L. (2001). Predicting transmembrane protein topology with a hidden Markov model: application to complete genomes. J. Mol. Biol. *305*, 567–580.

Lapaque, N., Hutchinson, J.L., Jones, D.C., Méresse, S., Holden, D.W., Trowsdale, J., and Kelly, A.P. (2009a). *Salmonella* regulates polyubiquitination and surface expression of MHC class II antigens. Proc. Natl. Acad. Sci. USA *106*, 14052–14057.

Lapaque, N., Jahnke, M., Trowsdale, J., and Kelly, A.P. (2009b). The HLA-DRalpha chain is modified by polyubiquitination. J. Biol. Chem. *284*, 7007–7016.

Léon, S., and Haguenauer-Tsapis, R. (2009). Ubiquitin ligase adaptors: regulators of ubiquitylation and endocytosis of plasma membrane proteins. Exp. Cell Res. *315*, 1574–1583.

McGuire, V.A., and Arthur, J.S. (2015). Subverting toll-like receptor signaling by bacterial pathogens. Front. Immunol. *6*, 607.

Mitchell, E.K., Mastroeni, P., Kelly, A.P., and Trowsdale, J. (2004). Inhibition of cell surface MHC class II expression by *Salmonella*. Eur. J. Immunol. *34*, 2559–2567.

Neefjes, J., Jongsma, M.L., Paul, P., and Bakke, O. (2011). Towards a systems understanding of MHC class I and MHC class II antigen presentation. Nat. Rev. Immunol. *11*, 823–836.

Niemann, G.S., Brown, R.N., Gustin, J.K., Stufkens, A., Shaikh-Kidwai, A.S., Li, J., McDermott, J.E., Brewer, H.M., Schepmoes, A., Smith, R.D., et al. (2011). Discovery of novel secreted virulence factors from *Salmonella enterica* serovar Typhimurium by proteomic analysis of culture supernatants. Infect. Immun. *79*, 33–43.

Omasits, U., Ahrens, C.H., Müller, S., and Wollscheid, B. (2014). Protter: interactive protein feature visualization and integration with experimental proteomic data. Bioinformatics *30*, 884–886.

Quah, B.J., and Parish, C.R. (2010). The use of carboxyfluorescein diacetate succinimidyl ester (CFSE) to monitor lymphocyte proliferation. J. Vis. Exp. http://dx.doi.org/10.3791/2259.

Randow, F., and Sale, J.E. (2006). Retroviral transduction of DT40. Subcell. Biochem. *40*, 383–386.

Roche, P.A., and Furuta, K. (2015). The ins and outs of MHC class II-mediated antigen processing and presentation. Nat. Rev. Immunol. *15*, 203–216.

Schroeder, N., Henry, T., de Chastellier, C., Zhao, W., Guilhon, A.A., Gorvel, J.P., and Méresse, S. (2010). The virulence protein SopD2 regulates membrane dynamics of *Salmonella*-containing vacuoles. PLoS Pathog. *6*, e1001002.

Tam, M.A., Rydström, A., Sundquist, M., and Wick, M.J. (2008). Early cellular responses to *Salmonella* infection: dendritic cells, monocytes, and more. Immunol. Rev. *225*, 140–162.

Tobar, J.A., González, P.A., and Kalergis, A.M. (2004). *Salmonella* escape from antigen presentation can be overcome by targeting bacteria to Fc gamma receptors on dendritic cells. J. Immunol. *173*, 4058–4065.

Tobar, J.A., Carreño, L.J., Bueno, S.M., González, P.A., Mora, J.E., Quezada, S.A., and Kalergis, A.M. (2006). Virulent *Salmonella enterica* serovar typhimurium evades adaptive immunity by preventing dendritic cells from activating T cells. Infect. Immun. *74*, 6438–6448.

van de Kooij, B., Verbrugge, I., de Vries, E., Gijsen, M., Montserrat, V., Maas, C., Neefjes, J., and Borst, J. (2013). Ubiquitination by the membrane-associated RING-CH-8 (MARCH-8) ligase controls steady-state cell surface expression of tumor necrosis factor-related apoptosis inducing ligand (TRAIL) receptor 1. J. Biol. Chem. *288*, 6617–6628.

Cell Host & Microbe, Volume *20*

Supplemental Information

The *Salmonella* Effector SteD Mediates MARCH8-Dependent Ubiquitination of MHC II Molecules and Inhibits T Cell Activation

Ethel Bayer-Santos, Charlotte H. Durkin, Luciano A. Rigano, Andreas Kupz, Eric Alix, Ondrej Cerny, Elliott Jennings, Mei Liu, Aindrias S. Ryan, Nicolas Lapaque, Stefan H.E. Kaufmann, and David W. Holden

Figure S1, related to Figure 2

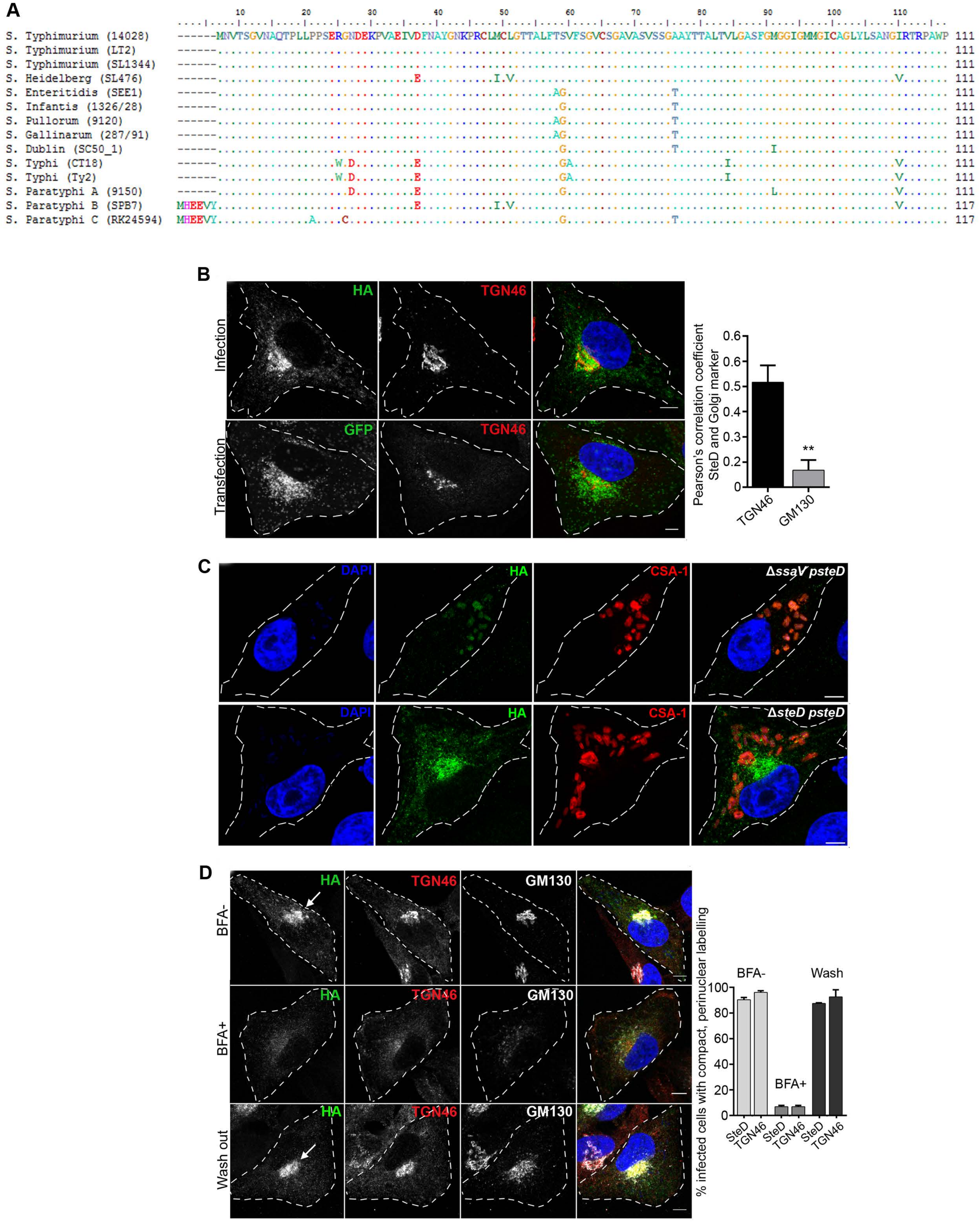

Figure S2, related to Figure 4

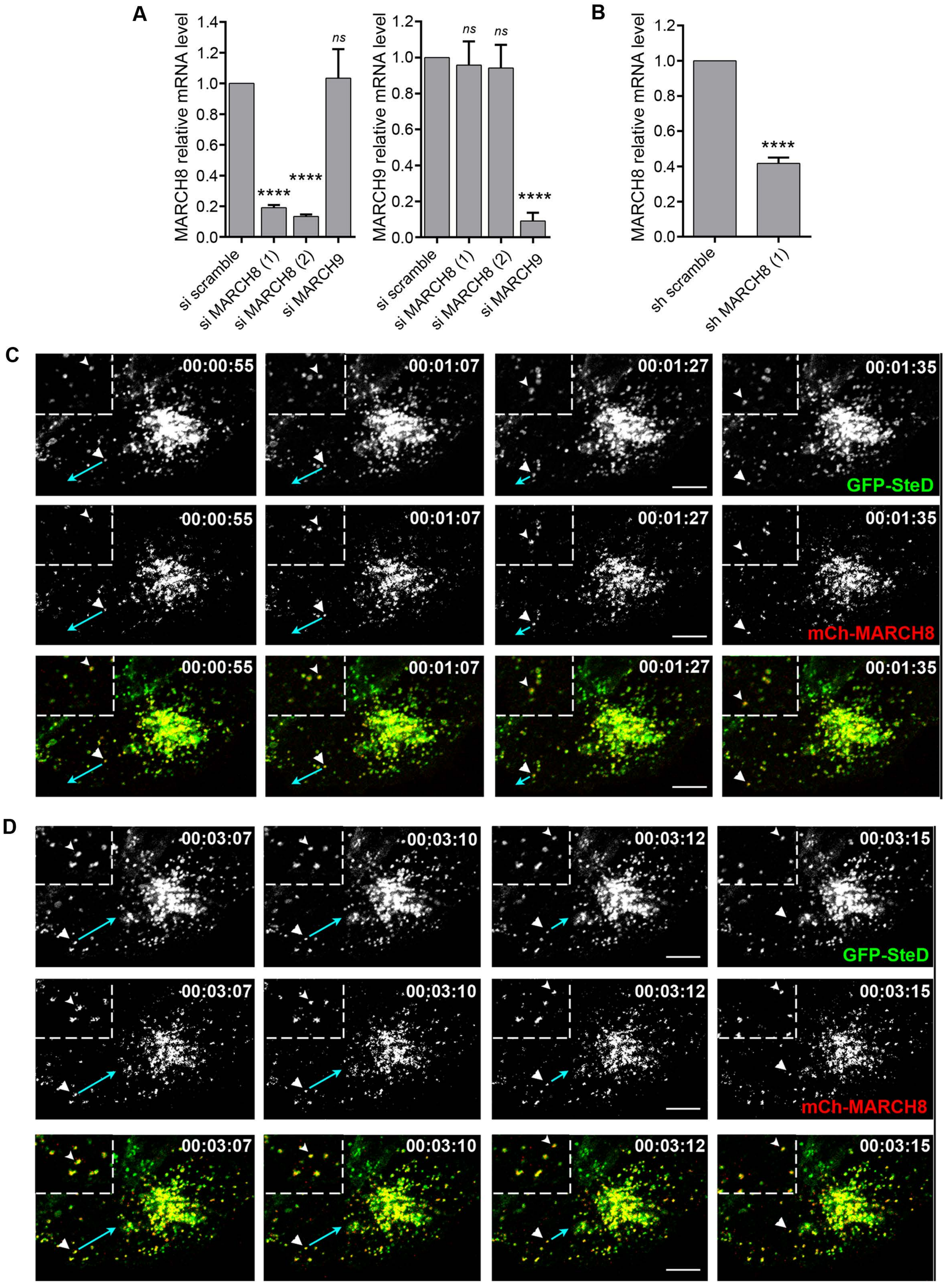

Figure S3, related to Figure 5

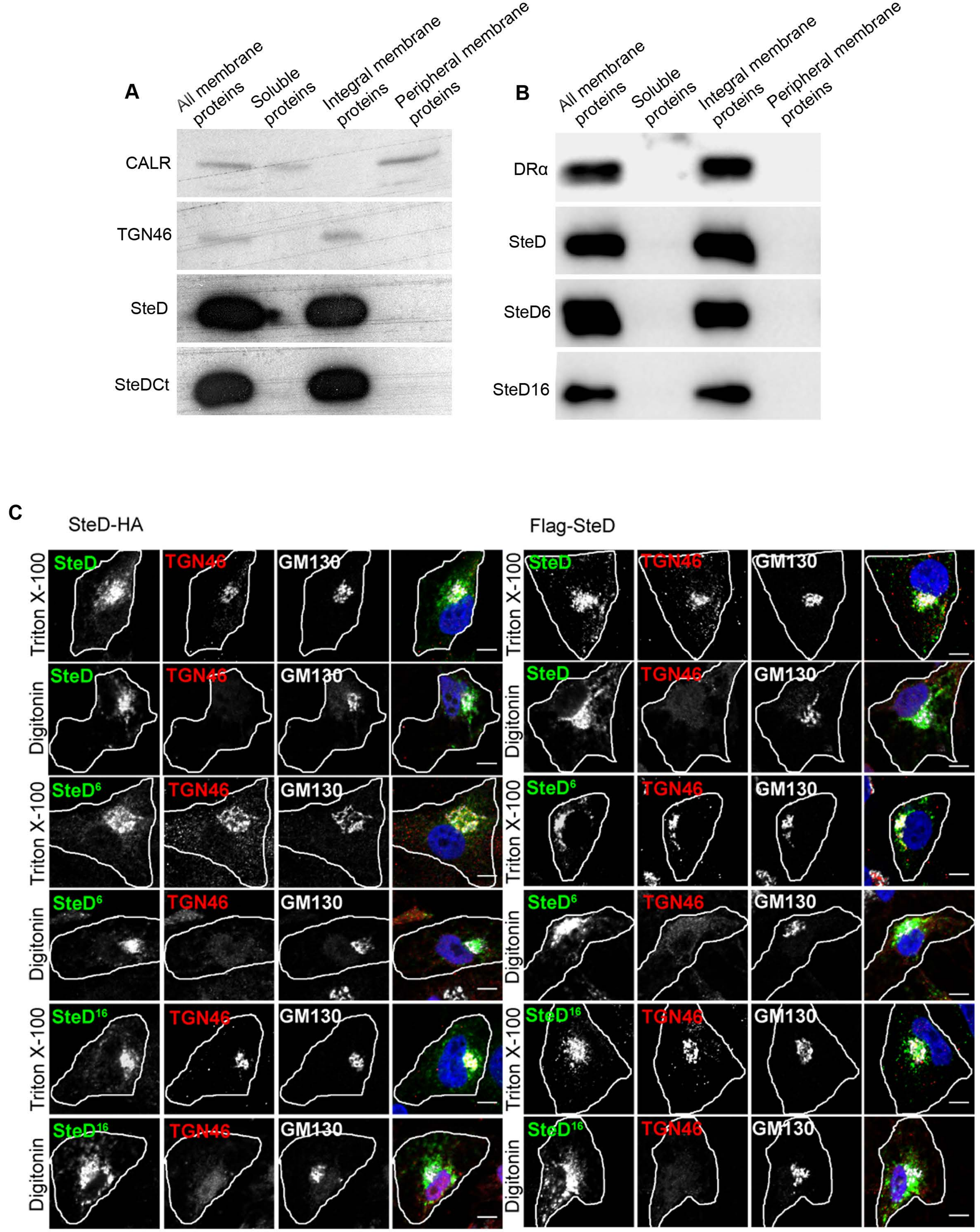

Figure S4, related to Figure 5

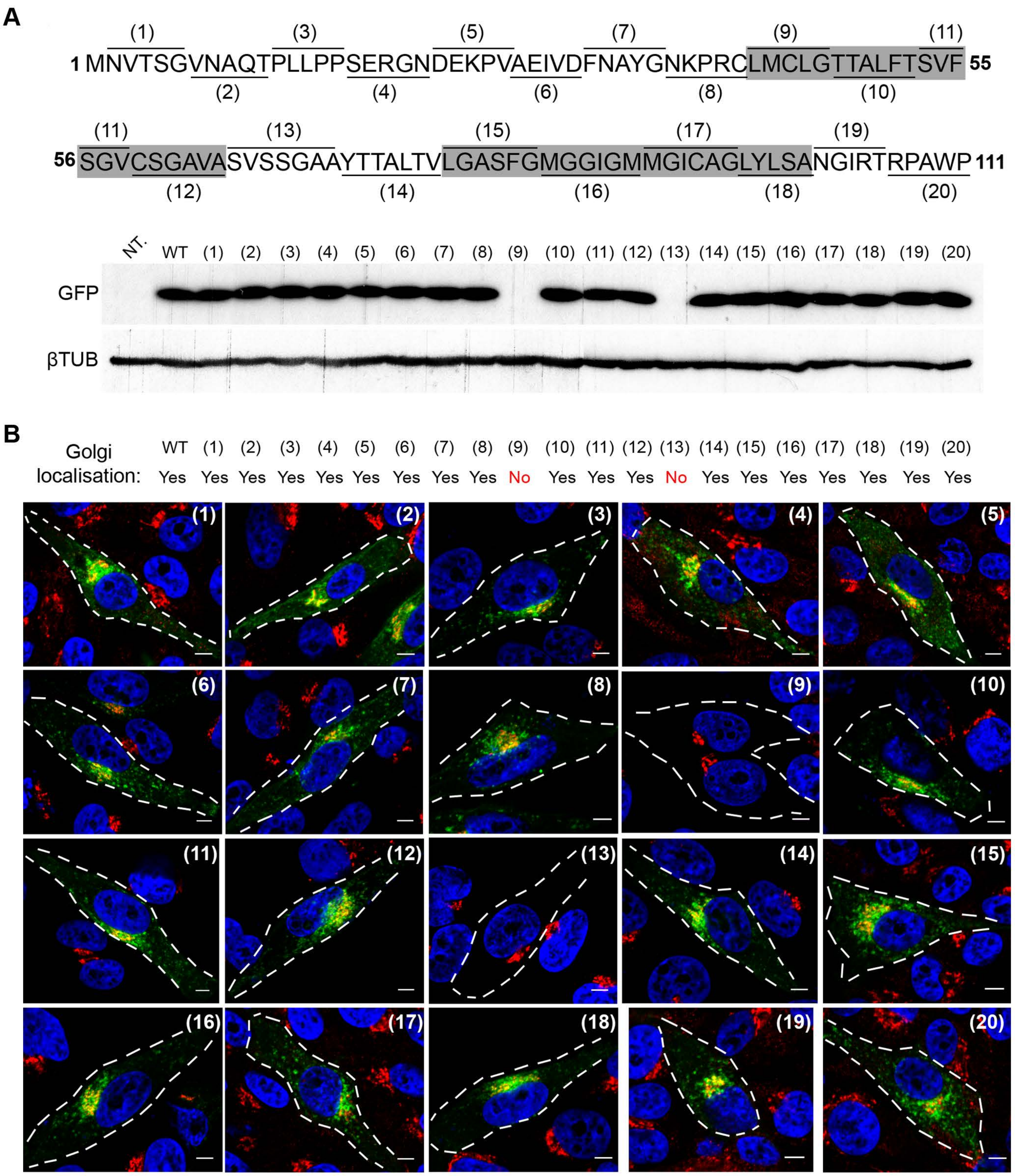

Figure S5, related to Figures 1, 3, 4 and 6

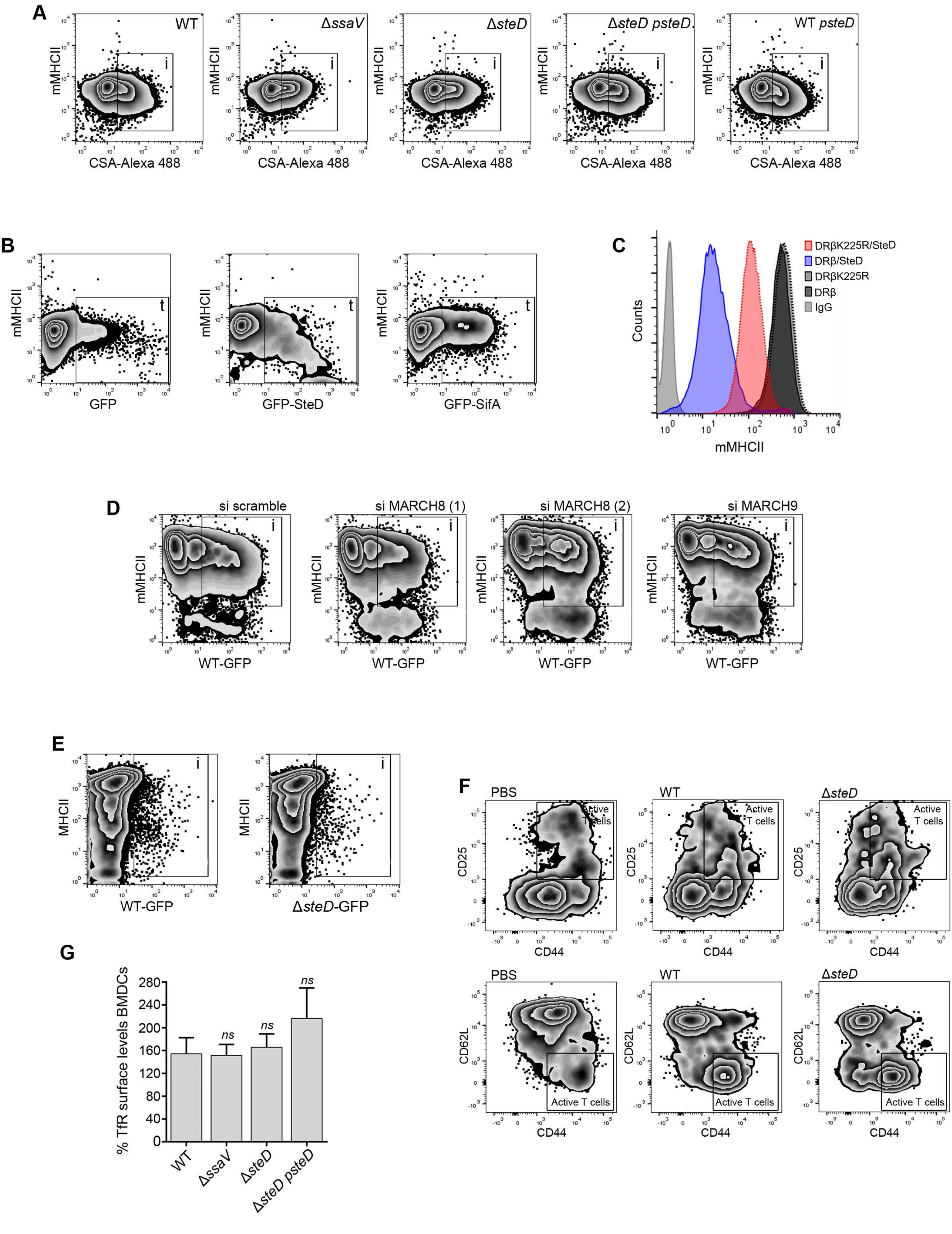

Legends to Supplemental Figures

Figure S1, related to Figure 2: *(A)* ClustalW alignment of amino acid sequence of SteD from different *Salmonella enterica* strains and serovars. Colours differentiate between amino acids with the same properties. *(B)* Confocal immunofluorescence microscopy of Mel JuSo cells at 20 h post-invasion with Δ*steD psteD* or post-transfection with M4P plasmid expressing GFP-SteD. Effector is shown in green (anti-HA or GFP) and TGN46 *trans*-Golgi marker is in red. Pearson's correlation coefficient of the Golgi region is shown on the right. **P<0.01 (Student's *t* test). *(C)* Confocal immunofluorescence microscopy of Mel JuSo cells at 20 h post-invasion by Δ*ssaV psteD-HA* or Δ*steD psteD-HA* strains of *S*. Typhimurium. Effector is shown in green (anti-HA) and *Salmonella* in red (anti-CSA-1). (D) Infected cells at 20 h post-invasion were incubated with Brefeldin A (BFA) for 90 min. Then, BFA was washed out and cells were incubated for another 90 min. Cells were fixed at different times and analyzed by immunofluorescence microscopy with anti-HA, anti-TGN46 and anti-GM130 antibodies. Scale bar - 5 µm. Quantification of the percentage of infected cells with a compact, perinuclear accumulation of SteD-2HA and TGN46 after exposure to BFA and wash out is shown on the right.

Figure S2, related to Figure 4: *(A)* Quantitative real-time PCR analysis showing mRNA levels of MARCH8 and MARCH9 in Mel JuSo cells after exposure to a scramble siRNA oligo, two oligos specific to MARCH8, or one oligo specific to MARCH9. *(B)* Quantitative real-time PCR analysis showing mRNA levels of MARCH8 in Mel JuSo cells after transduction with scramble or MARCH8 shRNAs. *(C,D)* Stable Mel JuSo cell lines expressing GFP-SteD were transfected with a vector expressing mCherry-MARCH8. After 20 h, cells were subjected to live imaging analysis by time lapse microscopy. Time points were extracted from Movie S2 showing vesicles positive for both GFP-SteD and mCherry-MARCH8 (arrowheads) displaying anterograde *(C)* and retrograde movement *(D)*. Light blue arrows indicate the direction of movement. Scale bar - 5 µm. Data in *(A)* were compared to si scramble by one-way ANOVA followed by Dunnett's multiple comparison test. ****P<0.0001, *ns* (not significant). Data in *(B)* were analysed by Student's *t* test. ****P<0.0001.

Figure S3, related to Figure 5: *(A)* Membrane fractionation of Mel JuSo cells infected for 20 h with Δ*steD psteD*^{Ct} or *(B)* stable cell lines expressing GFP-SteD^{6} or GFP-SteD^{16}. Soluble proteins were separated from total membrane proteins, which were later treated with 2.5 M urea to discriminate between integral membrane and peripheral membrane proteins by ultracentrifugation. Calreticulin (CALR) is a peripheral membrane protein, TGN46 is an integral *trans*-Golgi membrane protein and DRα is an integral membrane protein. *(C)* Mel JuSo cells were transfected with vector encoding SteD fused at its N-terminus to FLAG epitope (FLAG-SteD) (right panel) or to HA-tag at its C-terminus (left panel). Cells were semi- or completely permeabilised with digitonin or Triton X-100 to discriminate between cytoplasmic and Golgi luminal antigens, respectively. Antibodies recognising the luminal portion of TGN46 or cytoplasmic GM130 were used as controls. Scale bar - 5 µm.

Figure S4, related to Figure 5: *(A)* Schematic representation of blocks of SteD amino acids (over and underlined) that were substituted with alanines (top) and western blot analysis of Mel JuSo cells transfected with vectors expressing GFP-tagged mutated versions of SteD (bottom). WT, wild-type SteD. NT, non-transfected. Anti-GFP and anti-β tubulin antibodies were used. *(B)* Immunofluorescence microscopy of Mel JuSo cells 20 h after transfection with vectors expressing mutant versions of SteD. GFP-fusion proteins are in green and TGN46 labelling is in red. Nucleus is stained by DAPI in blue. Scale bar - 5 µm.

Figure S5, related to Figures 1, 3, 4 and 6: Representative flow cytometry zebra plots and histogram corresponding to fig. 1C. *(A)*, fig. 1D *(B)*, fig. 3F *(C)*, fig. 4A *(D)*, fig. 6E *(E)* and fig. 6F *(F)*. Boxed areas: i – infected; t – transfected. Mouse bone marrow-derived dendritic cells (BMDCs) were infected with the indicated bacterial strains and transferrin receptor (TfR) surface levels were quantified by flow cytometry at 20 h post-uptake. Surface levels of TfR in infected cells are represented as a percentage of those in uninfected cells from the same sample and are the means ± SD from three independent experiments. Data were compared to WT by one-way ANOVA followed by Dunnett's multiple comparison test, *ns* (not significant).

Movie S1, related to Figure 2: Stable Mel JuSo cell line expressing GFP-SteD.

Movie S2, related to Figure 4: Stable Mel JuSo cell line expressing GFP-SteD was transfected with vector expressing mCherry-MARCH8 and movie was acquired 20 h after transfection.

Table S1, related to Figures 1, 2, 3, 4, 5 and 6. Strains, plasmids and antibodies used in this study.

***S.* Typhimurium strains**

Name	Description	Reference
wild-type	12023 *S.* Typhimurium wild-type	NTCC
Δ*ssaV*	Δ*ssaV* ::km	Beuzon et al., 1999
Δ*sseL*	Δ*sseL* ::km	Mesquita et al., 2012
Δ*slrP*	Δ*slrP* ::km	Andreas Bäumler
Δ*sspH1*	Δ*sspH1* ::km	Figueira et al., 2013
Δ*sspH2*	Δ*sspH2* ::km	Figueira et al., 2013
Δ*gogB*	Δ*gogB* ::km	Figueira et al., 2013
Δ*sifA*	Δ*sifA* ::km	Beuzon et al., 2002
Δ*sifA* /Δ*sopD2*	Δ*sifA* ::km/Δ*sopD2*	Stéphane Méresse
Δ*sifB*	Δ*sifB* ::km	Figueira et al., 2013
Δ*sseJ*	Δ*sseJ* ::cm	Lossi et al., 2008
Δ*sseF*	Δ*sseF* ::km	Brumell et al., 2003
Δ*sseG*	Δ*sseG* ::km	Brumell et al., 2003
Δ*sopD2*	Δ*sopD2* ::cm	Figueira et al., 2013
Δ*pipB*	Δ*pipB* ::km	Figueira et al., 2013
Δ*pipB2*	Δ*pipB2* ::km	Figueira et al., 2013
Δ*sopD*	Δ*sopD* ::km	Figueira et al., 2013
Δ*srfJ*	Δ*srfJ* ::km	Figueira et al., 2013
Δ*srfH*	Δ*srfH* ::km	Figueira et al., 2013
Δ*sptP*	Δ*sptP* ::km	Figueira et al., 2013
Δ*steA*	Δ*steA* ::km	Figueira et al., 2013
Δ*steB*	Δ*steB* ::km	Figueira et al., 2013
Δ*steC*	Δ*steC* ::km	Mesquita et al., 2012
Δ*steD*	Δ*steD* ::km	This study
Δ*steE*	Δ*steE* ::km	This study
Δ*spvB*	Δ*spvB* ::km	Figueira et al., 2013
Δ*spvC*	Δ*spvC*	Figueira et al., 2013
Δ*spvD*	Δ*spvD* ::km	Figueira et al., 2013
Δ*sseK1*	Δ*sseK1* ::km	Figueira et al., 2013
Δ*sseK2*	Δ*sseK2* ::km	Figueira et al., 2013
Δ*sseK3*	Δ*sseK3* ::km	Figueira et al., 2013
Δ*sseK1* /Δ*sseK2* /Δ*sseK3*	Δ*sseK1* /Δ*sseK2* /Δ*sseK3* ::km	Figueira et al., 2013
Δ*cigR*	Δ*cigR* ::km	Figueira et al., 2013
Δ*gtgA*	Δ*gtgA* ::km	Figueira et al., 2013
Δ*gtgE*	Δ*gtgE* ::km	Figueira et al., 2013
Δ*avrA*	Δ*avrA* ::km	Figueira et al., 2013
wild-type SL3261	SL3261 (*aroA*)	Hoiseth et al., 1981
Δ*steD* SL3261	SL3261 (*aroA* /Δ*steD*)	This study.

Plasmids

Name	Description	Reference
pKD4	PCR template plasmid with Km resistance cassette (KmR)	Datsenko et al., 2000
pKD46	Plasmid encoding arabinose-inducible λ-Red recombinase system (CarbR)	Datsenko et al., 2000
pCP20	Plasmid encoding arabinose-inducible FLP recombinase (CarbR)	Datsenko et al., 2000
pFCcGi	rpsM::mCherry and PBAD::gfpmut3a promoter fusions in pFPV25.1 (CarbR)	Figueira et al., 2013
pFPV25.1	rpsM::gfpmut3a promoter fusion in pFPV25	Valdivia et al., 1996

pMD.GAGPOL	Retroviral helper plasmid encoding gag/pol proteins	Randow and Sale, 2006
pMD.VSVG	Retroviral helper plasmid encoding vesicular stomatitis virus glycoproteins	Randow and Sale, 2006
psteD	pWSK29 containing C-terminus 2HA-tagged steD-promoter and open reading frame (CarbR)	This study
GFP	M5P retroviral vector containing GFP open reading frame (CarbR/PuroR)	Teresa Thurston
GFP-SteD	M5P retroviral vector containing N-terminus GFP-tagged steD open reading frame (CarbR)	This study
GFP-SifA	M5P retroviral vector containing N-terminus GFP-tagged sifA open reading frame (CarbR)	McGourty et al., 2013
GFP-SseF	M5P retroviral vector containing N-terminus GFP-tagged sseF open reading frame (CarbR)	Teresa Thurston
GFP-SseG	M5P retroviral vector containing N-terminus GFP-tagged sseG open reading frame (CarbR)	Teresa Thurston
HA-DRβ	M5P retroviral vector containing N-terminus HA-tagged HLA-DR beta chain (CarbR/PuroR)	This study
HA-DRβ-K225R	M5P retroviral vector containing N-terminus HA-tagged HLA-DR beta chain with K225 mutated (CarbR/PuroR)	This study
MARCH8-FLAG	M5P retroviral vector containing C-terminus FLAG-tagged MARCH8 (CarbR/PuroR)	This study

Antibodies

Specificity	Clones	Use	Source
HLA-DR	L243	FACS/IP	Sigma-Aldrich
HLA-DRα	TAL.1B5	WB	Dako
HLA-DRβ	DA2	WB	Abcam
HA	3F10	IF	Roche
FLAG	M2	IF	Sigma-Aldrich
TGN46	Rabbit Polyclonal	IF/WB	LifeSpan Biosciences
GM130	35	IF	BD Biosciences
Golgin 97	CDF4	WB	Invitrogen
Calreticulin	Rabbit Polyclonal	WB	Thermo Scientific
CSA-1	Goat polyclonal	IF	KPL
Ubiquitin-HRP	P4D1	WB	Santa Cruz
Tubulin β	Rabbit monoclonal	WB	Abcam
HA	Rabbit Polyclonal	WB	Sigma-Aldrich
FLAG	Rabbit Polyclonal	WB	Sigma-Aldrich
GFP	Rabbit monoclonal	WB	Thermo Scientific
CD11c	N418	FACS	MiltenyiBiotec
TfR	H68.4	FACS	Zymed Laboratories
CD3ε	145-2C11	FACS	MiltenyiBiotec
CD4	RM4-5	FACS	BD Pharmingen
CD25	7D4	FACS	MiltenyiBiotec
CD44	IM7.8.1	FACS	MiltenyiBiotec
CD62L	MEL-14	FACS	MiltenyiBiotec
B7.2	PO3.3	FACS	MiltenyiBiotec
I-A/I-E	M5/114.15.2	FACS	Thermo Scientific

WB: western blot; FACS: flow cytometer; IP: immunoprecipitation; IF: immunofluorescence.

Table S2, related to Figure 6. Bacterial load (c.f.u.) per spleen of infected mice.

Group compared	WT	Δ*steD*
1	112000	124000
2	98000	96250
3	87500	94750
4	86000	84500
5	68750	70000
6	67300	64550
7	57000	60450
8	54275	54900
9	53150	54566
10	44700	45475
11	44825	47800
12	39000	36733

Supplemental Experimental Procedures

siRNA and shRNA transfection

Mel JuSo cells were seeded in 6-well plates and transfected on the following day with siRNA oligonucleotides using Lipofectamine RNAiMAX^{TM} (Life technologies) according to the manufacturer's instructions with a final concentration of 50 nM. Cells were reseeded 24 h after the first transfection and 24 h later a second round of transfection was done. Cells were infected 24 h after the second transfection and analysed 16-20 h post-invasion. siRNA oligonucleotides for MARCH8 (5'-TCCAGCGGGATTGACACTCAA, CTGCTAGAGTCTACAGAAGTA-3') and MARCH9 (5'-CAGGTTGGATGCCGTTGCAGA, CAGCACTCCGAGGTATCTAAA-3') were from Qiagen. A scrambled sequence (5'-AAACTTGTCGACGAGAAGCAA-3') was included in all experiments as a negative control. For shRNA construction, siRNA sequences for MARCH8 (5'-TCCAGCGGGATTGACACTCAA) and scramble (5'-AAACTTGTCGACGAGAAGCAA-3') were cloned into pSUPER vector.

Immunofluorescence microscopy

Cells were seeded onto coverslips and infected as described in the Experimental Procedures. For general procedures, cells were collected at 20 h post-invasion, washed with PBS and fixed in 3% paraformaldehyde in PBS for 10 min at room temperature. Cells were incubated with 50 mM NH_4Cl for 10 min, washed and labelled with appropriate antibodies diluted in 10% FCS and 0.1% saponin in PBS. Antibodies used for IF are listed in Table S1. For Brefeldin A (BFA) treatment, at 20 h post-invasion new medium containing 5 µg/ml of BFA (Sigma-Aldrich) was added to infected cells and incubated for 90 min. The washout was done by washing cells 5 times in PBS and incubating them in 10% DMEM for another 90 min prior to final wash and fixation for labelling. For selective permealisation, digitonin treatment was done using live cells. At 20 h post-invasion, cells were placed on ice, washed with KHM buffer (110 mM KOAc, 20 mM HEPES, 2 mM $MgCl_2$, pH 7.3) and incubated for 5 min with 40 µg/ml digitonin diluted in KHM. Cells were washed and incubated with primary antibodies diluted in 10% FCS in PBS for 30 min on ice. After washes, cells were fixed and incubated with secondary antibodies at room temperature under standard procedures. After fixation, cells were incubated with 0.1% Triton X-100 for 5 min at room temperature prior to labelling for 1 h with primary and then secondary antibodies diluted in 10% FCS and 0.1% saponin in PBS. For visualization of internalised mMHCII complexes, cells were placed on ice and incubated with mAb L243

diluted in DMEM containing 10% FCS for 30 min. Cells were washed, incubated with warm medium and returned to the incubator at 37 °C for another 4 h. After incubation, cells were washed, fixed and labelled with secondary antibody diluted in 10% FCS and 0.1% saponin in PBS.

Immunoprecipitation and immunoblots

For immunoprecipitation and immunoblotting, approximately 1 x 10^7 cells were harvested using Cell Dissociation Buffer (Sigma) and lysed in buffer containing 1% Nonidet P-40, 50 mM Tris-Cl (pH 7.4), 5 mM EDTA, 150 mM NaCl, protease inhibitors (Roche) and 10 mM iodoacetamide (IAA) for 30 min at 4°C. Lysate was centrifuged at 16,000 *g* for 15 min and post-nuclear supernatant was incubated with 30 µl of slurry solution containing CNBr sepharose-coupled mAb L243 or isotype control for 2 h at 4°C. Immunoprecipitates were washed with lysis buffer and eluted with 100 mM glycine (pH 3.0). Eluates were precipitated with four volumes of cold acetone, resuspended in SDS-PAGE loading buffer and analysed by western blot. For infected cells, the MOI was increased to 300:1 and infection time to 45 min. For co-immunoprecipitations, cells were lysed with 50 mM Tris-Cl (pH 7.4), 150 mM NaCl, 5 mM EDTA, 5% glycerol, 10 mM IAA and 0.5% Triton X-100 containing protease inhibitors for 15 min at 4°C. Lysate was centrifuged at 16,000 *g* for 15 min and post-nuclear supernatant was incubated with GFP-Trap (ChromoTek) or CNBr sepharose-coupled mAb L243 or isotype control for 2 h at 4°C. Immunoprecipitates were washed with lysis buffer and eluted with glycine as described above. Protein samples were separated by SDS-PAGE and transferred to Immobilon-P™ membrane (Millipore). Membranes were blocked in 5% skimmed milk and 0.1% Tween-20 in PBS and incubated with primary and secondary antibodies in the same solution, which were detected using ECL Plus™ Western Blotting Detection Reagents (Thermo Fisher). Band intensities were calculated using Image J software.

Supplemental References

Beuzon, C.R., Meresse, S., Unsworth, K.E., Ruiz-Albert, J., Garvis, S., Waterman, S.R., Ryder, T.A., Boucrot, E., and Holden, D.W. (2000). *Salmonella* maintains the integrity of its intracellular vacuole through the action of SifA. The EMBO journal *19*, 3235-3249.

Brumell, J.H., Kujat-Choy, S., Brown, N.F., Vallance, B.A., Knodler, L.A., and Finlay, B.B. (2003). SopD2 is a novel type III secreted effector of *Salmonella typhimurium* that targets late endocytic compartments upon delivery into host cells. Traffic *4*, 36-48.

Figueira, R., Watson, K.G., Holden, D.W., and Helaine, S. (2013). Identification of salmonella pathogenicity island-2 type III secretion system effectors involved in intramacrophage replication of *S. enterica* serovar typhimurium: implications for rational vaccine design. mBio *4*, e00065.

Hoiseth, S.K. and Stocker, B.A. (1981). Aromatic-dependent *Salmonella typhimurium* are non-virulent and effective as live vaccines. Nature 291, 238-239.

Lossi, N.S., Rolhion, N., Magee, A.I., Boyle, C., and Holden, D.W. (2008). The *Salmonella* SPI-2 effector SseJ exhibits eukaryotic activator-dependent phospholipase A and glycerophospholipid: cholesterol acyltransferase activity. Microbiology *154*, 2680-2688.

McGourty, K., Thurston, T.L., Matthews, S.A., Pinaud, L., Mota, L.J., and Holden, D.W. (2012). *Salmonella* inhibits retrograde trafficking of mannose-6-phosphate receptors and lysosome function. Science *338*, 963-967.

Mesquita, F.S., Thomas, M., Sachse, M., Santos, A.J., Figueira, R., and Holden, D.W. (2012). The *Salmonella* deubiquitinase SseL inhibits selective autophagy of cytosolic aggregates. PLoS pathogens *8*, e1002743.

Valdivia, R.H., and Falkow, S. (1996). Bacterial genetics by flow cytometry: rapid isolation of *Salmonella typhimurium* acid-inducible promoters by differential fluorescence induction. Molecular microbiology *22*, 367-378.
